# Artificial intelligence-assisted phenotyping of drug-resistant bacteria using a monosaccharide-based fluorescent sensor array

**DOI:** 10.1039/d6sc00084c

**Published:** 2026-05-19

**Authors:** Zhuo-Fan Zhang, Wen-Zhen Gui, Yi-Fan Tang, Hui-Qi Gan, Xi-Le Hu, Tony D. James, Shengbing Yang, Qian Liu, Xiao-Peng He

**Affiliations:** a Key Laboratory for Advanced Materials and Joint International Research Laboratory of Precision Chemistry and Molecular Engineering, Feringa Nobel Prize Scientist Joint Research Centre, School of Chemistry and Molecular Engineering. East China University of Science & Technology Shanghai 200237 China xlhu@ecust.edu.cn xphe@ecust.edu.cn; b National Center for Liver Cancer, The International Cooperation Laboratory on Signal Transduction. Eastern Hepatobiliary Surgery Hospital Shanghai 200438 China; c Academy of Pharmacy Xi'an-Jiaotong Liverpool University Suzhou 215123 China; d Department of Laboratory Medicine, Ren Ji Hospital 160 Pujian Road Shanghai 200127 China liuqian_rj@shsmu.edu.cn; e Shanghai Key Laboratory of Orthopedic Implants, Department of Orthopedic Surgery, Shanghai Ninth People's Hospital, Shanghai Jiao Tong University School of Medicine Shanghai 200011 China shengbingyang@shusmu.edu.cn; f Department of Chemistry, University of Bath Bath BA2 7AY UK t.d.james@bath.ac.uk; g School of Chemistry and Chemical Engineering, Henan Normal University Xinxiang 453007 China

## Abstract

Chemical tools capable of effectively phenotyping drug-resistant bacteria can help improve therapeutic efficacy toward bacterial infections. While conventional techniques rely on labor-intensive procedures for the determination of bacterial susceptibility to antibiotics, here we developed a sensor array based on fluorogen-labelled monosaccharides to accurately phenotype drug-resistant bacteria with the assistance of artificial intelligence (AI). d-Glucose, d-galactose, l-fucose and d-mannose, which are common monomeric building blocks of natural glycans, were labelled with a “conformationally-adaptive” fluorophore (DPAC) with two different linkers, giving rise to a sensor array that consists of eight fluorescent glycoprobes. Using homogeneous high-throughput screening, we found that all the glycoprobes exhibited sensitive ratiometric fluorescence changes in the presence of *Pseudomonas aeruginosa* (*P. aeruginosa*) expressing bacterial lectins (LecA and LecB) selective for d-galactose, l-fucose and d-mannose. However, minimal fluorescence changes were seen when the glycoprobes were incubated with other bacterial strains lacking lectin expression. The use of ensemble learning to process the acquired sensing signals further enabled the accurate discrimination of clinically isolated, drug-resistant *P. aeruginosa* from drug-sensitive strains. Interestingly, using AI-assisted array sensing, we also achieved the phenotyping of *P. aeruginosa* after long-term exposure to mechanistically different antibiotics, thus highlighting the effectiveness of this approach for precision medicine.

## Abstract 2

A monosaccharide-based fluorescent sensor array utilizing vibration-induced emission achieves the accurate, machine learning-assisted phenotyping of drug-resistant *Pseudomonas aeruginosa*.

## Graphical abstract

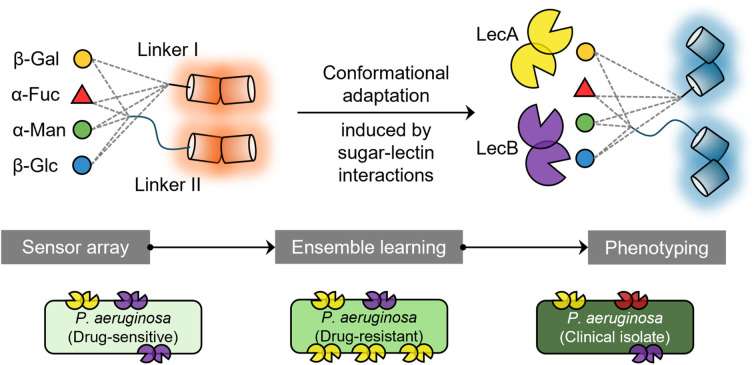

## Introduction

Bacterial infections are a global challenge for public health, with multidrug-resistant (MDR) pathogens posing serious threats.^1^ A representative example is *Pseudomonas aeruginosa* (*P. aeruginosa*), a Gram-negative, opportunistic pathogen designated by the World Health Organization (WHO)^2^ as a “critical priority” MDR bacterium due to its diverse drug-resistant mechanisms and rapid adaptive evolution. Infection with *P. aeruginosa* can lead to many clinical complications including pneumonia,^3^ bacteremia,^4^ burn-wound sepsis,^5^ and refractory chronic pulmonary colonization in cystic fibrosis patients.^6^ Notably, *P. aeruginosa* develops extensive drug resistance (XDR) or pandrug resistance (PDR) through mechanisms such as biofilm formation, efflux pump overexpression, and acquired β-lactamases, with a global carbapenem resistance rate of >30%.^1,7,8^ These emerging resistance patterns underscore the critical need for effective diagnostic approaches to accurately identify drug resistance.^9^

Current gold-standard methods-including bacterial culture, microscopic examination, biochemical assays, serological tests, nucleic acid hybridization, and PCR-face substantial limitations.^10^ They are often time-consuming (24–72 hours of culturing), exhibit suboptimal sensitivity/specificity (particularly for polymicrobial infections), require technical expertise, and cannot differentiate viable from non-viable bacteria.^11^ Such constraints compromise timely clinical interventions, especially in managing MDR infections where early appropriate antimicrobial therapy significantly impacts outcomes.^12,13^

In recent years, fluorescent sensor arrays have emerged as a promising analytical tool for the discrimination of environmental and biological samples.^14,15^ Sensor arrays offer significant advantages, such as high sensitivity, strong anti-interference ability, and reliable visual detection results, compensating for the shortcomings of traditional diagnostic methods. A variety of fluorescent sensor arrays based on different principles have been developed. One popular approach is the construction of an array of polymeric or small-molecule fluorescent probes that exhibit moderate affinities to multiple binding targets. The subsequent use of dimension-reduction analyses to process the generated data allows for the differentiation of analytes. For example, dual-fluorescence sensor arrays based on poly(arylene ethynylene) and fluorophores characteristic of aggregation-induced emission (AIE),^16^ fluorescent probes based on 3-hydroxyflavone derivatives,^17^ and nitroreductase-based “turn-on” fluorescent probes^18^ have been successfully used to establish sensor arrays that enable the classification of Gram-positive and Gram-negative bacteria. As such, we constructed a sensor array based on fluorogens that exhibit dual-fluorescence emission, for the phenotyping of methicillin-resistant *Staphylococcus aureus* (MRSA).^19^ Additionally, sensor arrays based on nanomaterials have been applied for bacterial sensing. For instance, Phillips and co-workers^20^ used gold nanoparticles and poly(phenylene ethynylene) to establish a sensor array capable of classifying twelve types of bacteria. Wang and co-workers^21^ developed a paper-based sensor array using functionalized carbon dots to achieve rapid identification of five types of bacteria with the aid of machine learning.

Despite the rapid progress in sensors for bacterial classification, the binding interaction between the developed probes and bacteria are mainly based on electrostatic attraction, which could easily be interfered with by electrolytes present in the test solutions. Therefore, the exploitation of more specific biological features of bacteria as sensing targets might improve diagnostic accuracy. As a proof-of-concept, here we developed a fluorescent sensor array that consists of monosaccharides that target the virulence-associated lectins of *P. aeruginosa*, LecA (PA-IL, galactose-selective) and LecB (PA-IIL, fucose- and mannose-selective).^22,23^ These lectins have been shown to modulate pathoadaptive resistance of *P. aeruginosa* by facilitating biofilm formation and immune evasion, and can coordinate with resistance mechanisms such as efflux pumps (*e.g.*, MexAB-OprM) and carbapenemases, particularly in high-risk clones (*e.g.*, ST235/ST111).^8^ Clinically, these adaptations lead to the generation of MDR, XDR or even PDR phenotypes, underscoring the need for rapid and accurate diagnostics to guide antibiotic selection, thus overcoming treatment failures in critical care.^24–27^

**Fig. 1:**
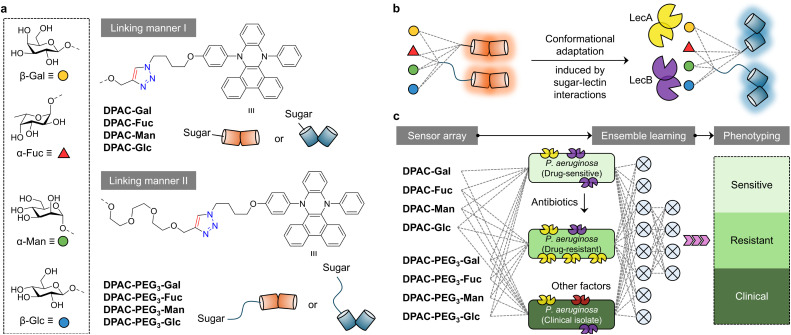
(a) Structure of the DPAC-based fluorescent glycoprobes with different linkers between sugars and the fluorogen. Schematic illustration of (b) the conformational adaptation of the glycoprobes upon sugar–lectin interactions to produce ratiometric fluorescence changes, and (c) the construction of a homogenous sensor array for the ensemble learning-assisted phenotyping of *P. aeruginosa* variants (drug-sensitive, drug-resistant and clinically isolated) with varying expression levels of LecA and LecB.

To the best of our knowledge, this study reports the first fluorescent sensor array that targets bacterial lectin-mediated resistance signatures, offering a useful tool for the early intervention of bacterial infections as well as outbreak containment. We used previously developed DPAC (*N*,*N*′-disubstituted-dihydrodibenzo[*a*,*c*]phenazines)-based fluorogen characteristic of “conformational adaptivity”^28,29^ to label four common monosaccharides, d-glucose, d-galactose, l-fucose and d-mannose, using two different linkers and click chemistry (Fig. 1a). These glycoprobes exhibited ratiometric fluorescence changes upon lectin binding (Fig. 1b). Subsequently, a sensor array that consists of the eight glycoprobes was subsequently established for bacterial classification. We determined that targeting their endogenous LecA and LecB expression, as well as the up or downregulation of both lectins under different antibiotic stress gave rise to the accurate phenotyping of drug-sensitive, drug-resistant and clinically isolated *P. aeruginosa* strains with the assistance of artificial intelligence (AI) (Fig. 1c).

## Results and discussion

The sensor array constructed in this study consists of eight monosaccharide-based glycoprobes (DPAC-Gal, DPAC-PEG_3_-Gal, DPAC-Fuc, DPAC-PEG_3_-Fuc, DPAC-Man, DPAC-PEG_3_-Man, DPAC-Glc, DPAC-PEG_3_-Glc) (Fig. 1a). Given that the sugar-binding properties of bacteria can differ largely, we anticipated that these glycoprobes would generate distinct sensing signals for different bacterial species, thus facilitating classification (Fig. 1b). The probes were synthesized by conjugating d-glucose, d-galactose, d-mannose and l-fucose through linkers of varying lengths with DPAC using the Cu(i)-catalyzed azide–alkyne cycloaddition click reaction. As shown in Schemes S1 and S2, the four monosaccharides were first glycosylated with propargyl alcohol or an *O*-alkynyl trimeric polyethylene glycol (PEG_3_) under rigorously anhydrous and anaerobic conditions using boron trifluoride diethyl ether (BF_3_·Et_2_O) for 6 hours. The resulting eight intermediates (G1–G8) were then treated with an azide-modified DPAC derivative (DPAC-N_3_) in the presence of Cu_2_SO_4_·H_2_O and sodium ascorbate at room temperature for 6 hours to afford G9–G16 in 85–95% yields, respectively. Finally, deacetylation of G9–G16 gave the desired glycoprobes in almost quantitative yields. Besides the glycoprobes, two control fluorogens without sugar modification (DPAC-OH and DPAC-PEG_3_-OH) were also synthesized as negative controls for subsequent binding assays (Schemes S1 and S2).

With the probes in hand, we first evaluated their photophysical properties using UV-vis and fluorescence spectroscopy in phosphate buffered saline (PBS) with 0.02% Tween 20 solution. We found that the glycoprobes exhibited UV-vis absorption maximum over a range of 354–365 nm (Fig. S1a–e), characteristic of DPAC.^30^ Two fluorescence emission bands centered at 452–493 nm and 590–616 nm, which are assigned to a constrained and planar conformation of DPAC,^30^ respectively, were detected in their representative fluorescence spectra (Fig. S1f–j). The observation of dual fluorescence emission suggests that the amphiphilic DPAC-based glycoprobes, with the presence of the hydrophilic sugar groups, maintain equilibrium between the constrained and planar conformation of DPAC. Notably, the red (*I*_601–616_)/blue (*I*_452–493_) fluorescence intensity ratios of all probes were determined to be similar irrespective of variations in sugar structure and linker chain length. This permits a minimal analytical bias in the following sensing assays that might be caused by variations in the initial conformational state (*i.e.*, large differences in fluorescence intensity ratios) of the probes. The control probes (DPAC-PEG_3_-OH and DPAC-OH) exhibited similar UV-vis absorption and dual-fluorescence emission profiles to those of the glycoporbes (Fig. S1e and j). This observation confirms that the conjugation of monosaccharide moieties does not alter the inherent photophysical properties and the initial conformational equilibrium of the DPAC core.

**Fig. 2:**
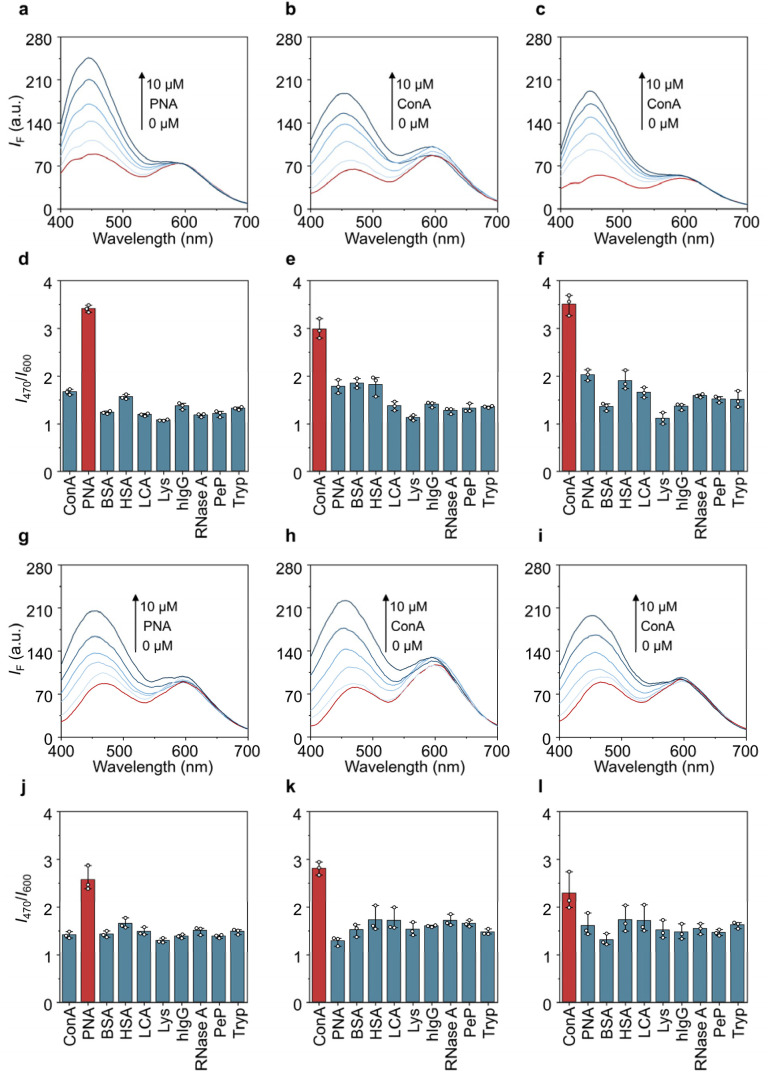
Fluorescence emission spectra of (a) DPAC-PEG_3_-Gal with increasing concentrations of PNA, (b) DPAC-PEG_3_-Glc with increasing concentrations of ConA, (c) DPAC-PEG_3_-Man with increasing concentrations of ConA, (g) DPAC-Gal with increasing concentrations of PNA, (h) DPAC-Glc with increasing concentrations of ConA, and (i) DPAC-Man with increasing concentrations of ConA. Changes in fluorescence intensity ratio (*I*_470_/*I*_600_) of (d) DPAC-PEG_3_-Gal, (e) DPAC-PEG_3_-Glc, (f) DPAC-PEG_3_-Man, (j) DPAC-Gal, (k) DPAC-Glc, and (l) DPAC-Man in the presence of different proteins (10 µM). All measurements were done in PBS (0.01 M, pH 7.4, containing 0.02% (v/v) Tween 20) at a probe concentration of 10 µM and an excitation at 360 nm.

Next, we evaluated the sensing properties of the glycoprobes for plant lectins that selectively bind monosaccharides. Concanavalin A (ConA – glucose and mannose selective), peanut agglutinin (PNA – galactose selective) and lentil agglutinin (LCA – core-fucose and high-mannose selective) were selected. After the addition of increasing concentrations (1–10 µM) of lectins to the glycoprobes, a concentration-dependent increase in their blue fluorescence emission intensity (465–487 nm) was observed when a selective lectin was mixed with the corresponding glycoprobe (*i.e.*, ConA with DPAC-Man and DPAC-PEG_3_-Man, DPAC-Glc and DPAC-PEG_3_-Glc; PNA with DPAC-Gal and DPAC-PEG_3_-Gal) (Fig. 2). In contrast, the red emission intensities (600–620 nm) of the probes remained relatively unchanged (Fig. 2). These results suggest that the selective recognition between monosaccharides and lectins draws the adjacent DPAC core into a peripheral domain of the protein. Supramolecular interactions (*e.g.*, hydrophobic and electrostatic forces) with the surrounding amino acid residues restrict the intramolecular vibrations of DPAC, thus stabilizing its bent, constrained conformation. This binding-induced conformational restriction effectively triggers the ratiometric red-to-blue fluorescence shift, a mechanism that is consistent with our previous Small-Angle X-ray Scattering (SAXS) investigations on DPAC-protein interactions^19,31^

Furthermore, a range of other physiological proteins, including human serum albumin (HSA), bovine serum albumin (BSA), lysozyme (Lys), human immunoglobulin G (hIgG), ribonuclease A (RNase A), pepsin (PeP), and trypsin (Tryp), were used as negative controls. When treating the glycoprobes with an unselective lectin and other control proteins, their red/blue emission ratios were only slightly changed, underscoring that the spectral changes seen arose mainly from selective lectin–monosaccharide interactions (Fig. S2–S9). This specificity was further supported by using the control probes (DPAC-OH and DPAC-PEG_3_-OH), which exhibited negligible ratiometric changes upon incubation with the same lectins (Fig. S10–S12), precluding the interference of non-specific hydrophobic interactions.

Having established the ratiometric fluorescence responses of the DPAC-based glycoprobes upon lectin recognition, we proceeded to construct a fluorescent sensor array for differential sensing of bacterial species. This design adopts the sensor-array concept pioneered by Anslyn and co-workers,^32^ and employs a panel of receptors exhibiting moderate cross-reactivity toward multiple analytes. The resulting response patterns were then processed *via* multivariate statistical analyses (*e.g.*, PCA) to generate “fingerprints” for individual analytes^33^-an approach having been successfully implemented for differentiating biologically relevant species.^34^ We therefore envisioned that the differential changes in the blue and red emission intensities of the glycoprobes upon bacterial binding could enable classification.

The bacterial strains used include *P. aeruginosa* (ATCC 27853 and clinically-isolated PA 3887 and PA A258), *Klebsiella oxytoca* (*K. oxytoca*, ATCC 13883), *Acinetobacter baumannii* (*A. baumannii*, ATCC 19606), *Escherichia coli* (*E. coli*, ATCC 25922 and ATCC 35218), *Staphylococcus aureus* (*S. aureus*, ATCC 29213 and ATCC 43300), *Staphylococcus epidermidis* (*S. epidermidis*, CMCC 26069) and *Salmonella typhi* (*S. Typhi*, CMCC 32206). Of note are *P. aeruginosa* and *A. baumannii*, which are known to express bacterial lectins. *P. aeruginosa* expresses LecA (galactose-selective) and LecB (fucose and mannose-selective), two virulent lectins that mediate biofilm formation and host infection. *A. baumannii* expresses the Acinetobacter trimeric autotransporter adhesin (Ata, galactose-selective) that functions as a virulence factor mediating bacterial adhesion to host cells and facilitating biofilm formation.^35^ These two bacteria are thus considered to be ideal targets for our monosaccharide-based glycoprobes. Other test bacteria that lack lectin expression were used as controls. To initiate the sensing, the eight glycoprobes were added to a 96-well microplate, followed by an orthogonal incubation with the bacteria. Then, the fluorescence intensity changes of the resulting mixtures at 470 nm and 600 nm were recorded in a high-throughput manner.

We first found that the ratiometric changes (*I*_470_/*I*_600_) of all glycoprobes changed most significantly in the presence of *P. aeruginosa* strains over time, with the standard ATCC 27853 strain inducing the strongest signal variations. Generally, probes incorporating a shorter linker elicited higher signal responses than the PEG-modified equivalents, which suggests that the shorter linker favors binding between glycoprobes and bacterial lectins. Among the LecB-targeting glycoprobes, the fucosylated variant outperformed the mannosylated one, which agrees with a previous study suggesting a higher affinity of fucosides than mannosides to LecB.^36^ An unexpected observation was the ratiometric changes of glucose-modified probes (DPAC-PEG_3_-Glc and DPAC-Glc) with ATCC 27853. This may be attributed to the structural similarity between glucose and mannose (C2 epimer to each other), which results in unselective binding with bacterial lectins.

To further corroborate the binding, a fluorescence imaging experiment was carried out using confocal laser-scanning microscopy with Syto-9 co-staining. We found that the galactose-based glycoprobes (DPAC-Gal and DPAC-PEG_3_-Gal) effectively adhered to *P. aeruginosa* (ATCC 27853) as evidenced by the good colocalization of the blue/red dual emission of DPAC with the green fluorescence of Syto-9. Furthermore, these glycoprobes visibly induced the aggregation of the bacteria. In sharp contrast, the control probes (DPAC-OH and DPAC-PEG_3_-OH) exhibited minimal fluorescence on the bacteria and failed to induce bacterial clustering (Fig. S13). This agrees with earlier reports that glycosylated materials trigger bacterial clustering.^37^

To further verify that the fluorescence responses on the bacterial surface were exclusively mediated by specific sugar–lectin interactions, a competitive binding assay was performed. *P. aeruginosa* (ATCC 27853) was pre-incubated with varying concentrations (1, 5, and 10 mM) of free monosaccharides (d-galactose, l-fucose, d-mannose, and d-glucose) for 1 h prior to adding the corresponding glycoprobes (DPAC-Gal, DPAC-Fuc, DPAC-Man, and DPAC-Glc). As depicted in Fig. S14, the fluorescence enhancements in the blue emission channel were significantly attenuated in a concentration-dependent manner, confirming that the free sugars competitively occupied the lectin binding sites on the bacteria.

Similarly, time-dependent ratiometric changes were seen for the glycoprobes with *A. baumannii* (ATCC 19606), which expresses the galactose-selective Ata protein and mannose-selective chaperone-usher pilus. However, the signal variations of the probes for this bacterium appeared to be weaker than those for *P. aeruginosa*. Finally, we found that strains lacking bacterial lectins showed minimal responses over time for the PEG-containing probes, and the probes with the shorter linker exhibited a gradual signal decline (Fig. S15). These data collectively constitute a characteristic “fingerprint” for each bacterial strain.

Next, we used a series of different techniques including Principal Component Analysis (PCA), Linear Discriminant Analysis (LDA), Multidimensional Scaling (MDS) and Factor Analysis (FA) to process the acquired fluorescence-based data for bacterial classification. Both *I*_470_/*I*_600_ ratios and independent changes in *I*_470_ and *I*_600_ were processed. The results showed a better classification outcome from analyzing independent fluorescence intensity changes of the glycoprobes (Fig. S16 and S17), probably because of the inclusion of more variables. However, the overall classification result appeared to be suboptimal as different *P. aeruginosa* strains failed to be sufficiently differentiated. This prompted us to resort to AI to better differentiate those strains.

**Fig. 3:**
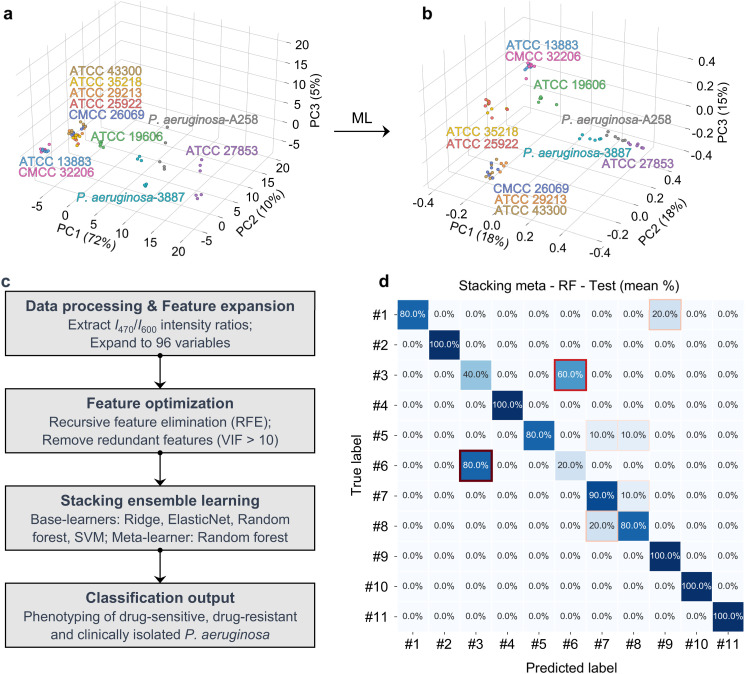
(a) Initial 3D PCA score plot for the discrimination of different bacterial strains. (b) Enhanced 3D PCA plot demonstrating improved cluster separation following machine learning (ML) optimization. (c) Schematic illustration of the optimized machine-learning workflow for classification models. (d) Confusion matrix of the stacking ensemble model (meta-learner: random forest (RF)) evaluating the final classification accuracy. Labels #1–#11 correspond to ATCC 13883, ATCC 19606, ATCC 25922, ATCC 27853, ATCC 29213, ATCC 35218, ATCC 43300, CMCC 26069, CMCC 32206, PA 3887, and PA A258, respectively.

Initially, we observed that the traditional PCA-only workflow resulted in heavily overlapping principal-component clouds, which failed to achieve effective discrimination among the different bacterial strains (Fig. 3a). In contrast, the 3D score plot generated from the stacking-based ensemble learning model demonstrated clear separation among *P. aeruginosa* strains (ATCC 27853, PA A258 and PA 3887), *A. baumannii* ATCC 19606, and the lectin-negative panel (ATCC 13883, ATCC 25922, ATCC 29213, ATCC 35218, ATCC 43300, CMCC 26069 and CMCC 32206) (Fig. 3b). This analysis further enabled differentiation between the drug-sensitive ATCC 27853 strain and the other two clinically isolated *P. aeruginosa* strains (PA A258 and PA 3887). The confusion matrix derived from this ML processing indicated nearly 100% accuracy, suggesting high model reliability (Fig. 3c).

In the method development phase, we first screened a set of eight different machine-learning (ML) algorithms—namely, K-Nearest Neighbors (KNN), Support Vector Machine (SVM), Ridge Regression, Lasso Regression, XGBoost, ElasticNet, LightGBM, and Random Forest—to process the dual fluorescence emission intensities produced by the glycoprobes in the presence of different bacterial strains (Fig. S18). These algorithms, grounded in distinct mathematical principles and intrinsic logic (distance-based, margin-maximising, regularised-linear, ensemble-boosting and bagging), generated a spectrum of classification outcomes for bacterial classification. Subsequently, we applied a three-level data fusion strategy, which significantly enhanced the performance of the typing model. Through a comprehensive comparison of macro F1 scores and classification accuracy across both training and test sets, we identified a stacking-based ensemble learning method as the optimal approach for differentiating all 11 bacterial strains. This ensemble framework employed Random Forest (RF) as the meta-learner, trained on the probability outputs of four base learners—Ridge, ElasticNet, RF and SVM—each of which was first optimized using feature sets selected *via* Recursive Feature Elimination (RFE) combined with Variance Inflation Factor (VIF) analysis. The overall workflow is summarized in Fig. 3d, and the confusion matrices used for ensemble selection are provided in Fig. S19.

To further validate that the discriminatory power of our sensor array inherently originates from selective monosaccharide-lectin interactions rather than non-specific background signals, the sensing performance of the control probes (DPAC-OH and DPAC-PEG_3_-OH) towards the four lectin-expressing strains (ATCC 27853, PA A258, PA 3887 and ATCC 19606) was evaluated. As expected, these non-targeting control probes exhibited negligible time-dependent fluorescence responses. In addition, when these non-specific signals were processed using the same optimized ensemble learning workflow, the model completely failed to differentiate the bacterial strains, yielding severely overlapping clusters and poor classification accuracies in both training and test sets (Fig. S20). This experiment suggests that the sugar moieties are indispensable for the AI-assisted phenotyping to be successful.

**Fig. 4:**
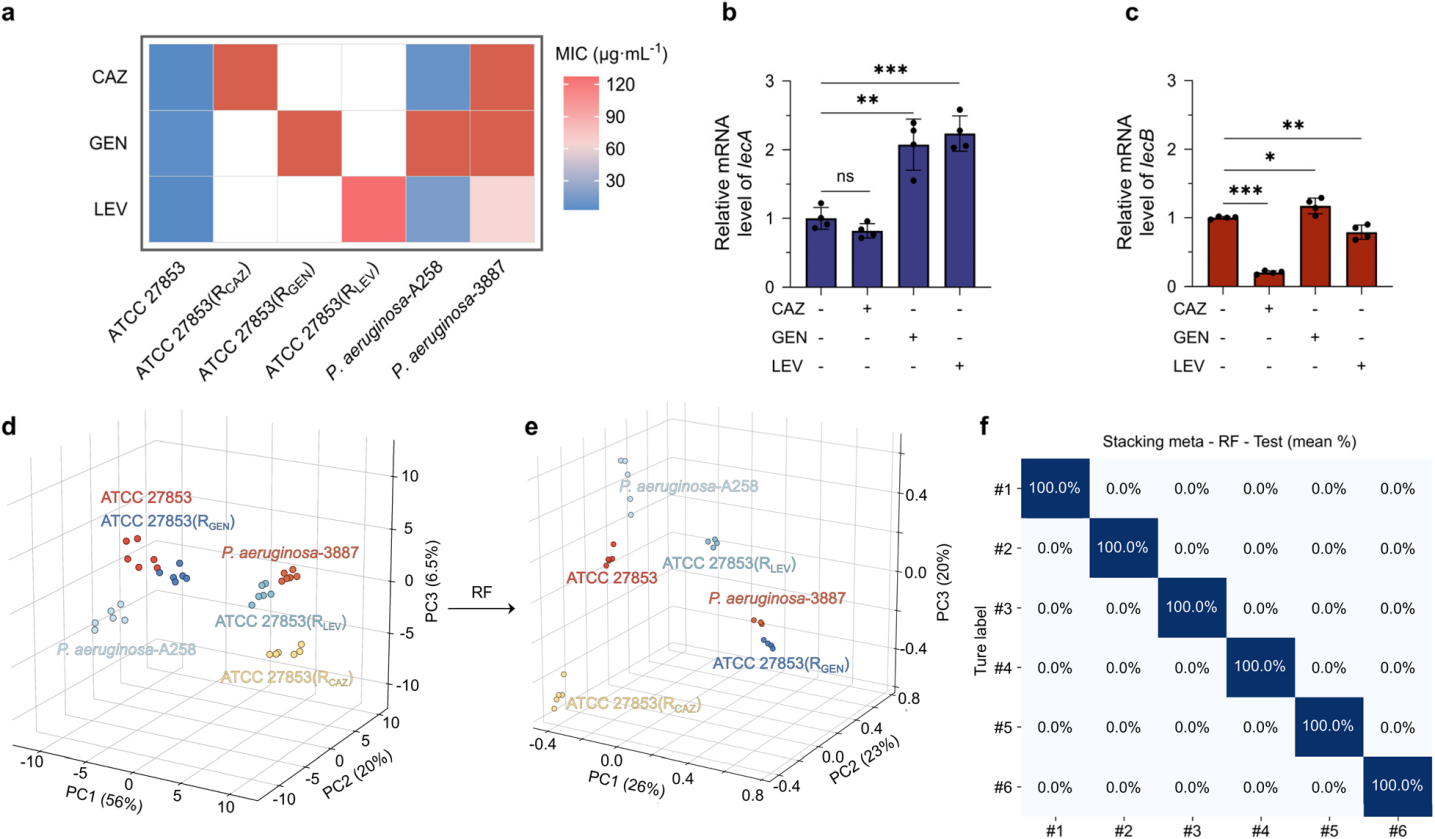
(a) Drug-resistance profiles of the laboratory-induced mutants and two clinical isolates. Relative mRNA levels of (b) lecA and (c) lecB across the different resistant strains. (d) Initial 3D PCA plot of the sensitive, induced-resistant, and clinical strains. (e) Enhanced 3D PCA classification performance based on the optimized RF algorithm. (f) Confusion matrix of the RF algorithm classification (Labels #1–#6 correspond to ATCC 27853, ATCC 27853(R_CAZ_), ATCC 27853(R_GEN_), ATCC 27853(R_LEV_), PA 3887, and PA A258, respectively).

Given that the method is capable of differentiating drug-sensitive and drug-resistant *P. aeruginosa*, we sought its utility for monitoring the generation of drug resistance of bacteria. For this purpose, we treated ATCC 27853 with Gentamicin (GEN), Levofloxacin (LEV) and Ceftazidime (CAZ) to induce drug resistance. We determined that the resulting variants ATCC 27853(R_CAZ_), ATCC 27853(R_GEN_) and ATCC 27853(R_LEV_) were resistant against the corresponding antibiotics with a minimum inhibitory concentration (MIC) of >128 µg mL^−1^. MIC tests were also done for PA A258 and PA 3887, and the result showed that PA A258 was mainly GEN-resistant and PA 3887 was insensitive to all three antibiotics tested (Fig. 4a). A subsequent measurement by real-time quantitative polymerase chain reaction (RT-qPCR) indicated changes in lectin expression levels in these strains (Fig. 4b and c). While a downregulation in LecB expression was seen for ATCC 27853(R_CAZ_), an upregulated LecA expression was consistently found for ATCC 27853(R_GEN_) and ATCC 27853(R_LEV_), with respect to ATCC 27853. These results agree with previous reports revealing the close relationship between antibiotic resistance and lectin expression.^38^

The variations in lectin expression levels under antibiotic stress can be attributed to the bacterium's adaptive evolution strategies. Continuous exposure to antibiotics drives *P. aeruginosa* to enhance biofilm formation as a protective barrier, leading to the compensatory upregulation of structural Extracellular Matrix (ECM) components like LecA. Furthermore, the prolonged antimicrobial pressure often triggers the rewiring of the global quorum sensing (QS) networks, resulting in profound fluctuations in the expression of downstream effectors such as LecB. Such variations also reflect the metabolic fitness trade-offs as the bacteria prioritize critical resistance mechanisms over non-essential protein synthesis.^39^

**Fig. 5:**
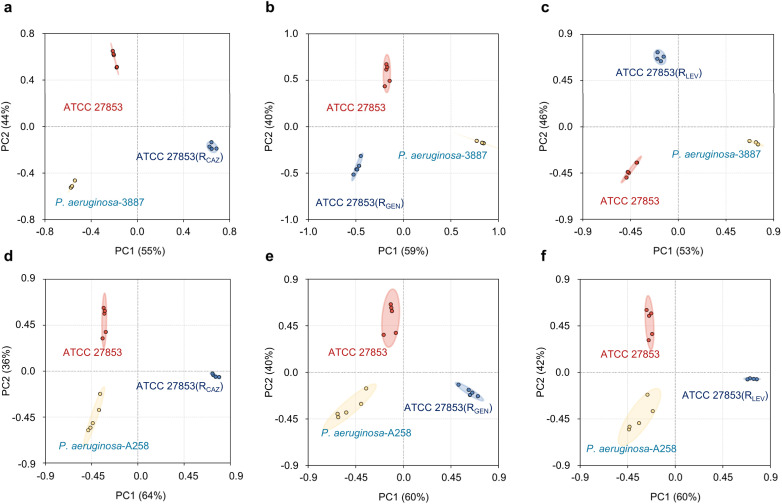
2D PCA clustering based on optimized RF model analysis for discriminating drug-resistant from drug-sensitive *P. aeruginosa* strains. Panels (a–f) are different training-testing set combinations: (a) ATCC27853, *P. aeruginosa* 3887 and ATCC 27853(R_CAZ_), (b) ATCC27853, PA 3887 and ATCC 27853(R_GEN_), (c) ATCC27853, PA 3887 and ATCC 27853(R_LEV_), (d) ATCC27853, PA A258 and ATCC 27853(R_CAZ_), (e) ATCC27853, PA A258 and ATCC 27853(R_GEN_), and (f) ATCC27853, PA A258 and ATCC 27853(R_LEV_).

We then incubated these strains with the glycoprobes, and obtained their “fingerprints” as shown in Fig. S18. Processing the obtained data with the RF algorithm led to differentiation of drug-resistant strains from drug-sensitive ones. Compared with clustering based on PCA (Fig. 4d), the RF algorithm produced apparently well-separated clusters for the distinct resistant subtypes (Fig. 4e). The corresponding confusion matrix indicated a classification accuracy of 100% (Fig. 4f), suggesting reliability. Generally, each ATCC 27853 variant could be well-differentiated from the drug-sensitive ATCC 27853 as well as a clinical strain, suggesting their different monosaccharide-binding profiles. This differentiation is likely a result of the differential lectin expression of the bacteria, a consequence of divergent resistance mechanisms. The distinct fingerprint patterns between the resistance-induced strains and clinical isolates suggest that the drug-resistance nature of the latter is more complicated. Furthermore, resistance may not only involve changes in surface lectin expression but also other structural and biochemical features, which collectively contribute to the divergent detection outcomes. Notably, after preprocessing with the RF algorithm, all six strains could be fully discriminated from one another in a two-dimensional PCA projection, further validating the effectiveness of the selected feature set (Fig. 5). To distil the most relevant features from this complex data, we employed RF for feature selection. This allowed us to identify a minimal discriminant feature set, achieving clear differentiation between the ATCC 27853 strain and its resistant variants using as few as 16 features (Fig. S22 and S23). These results suggest our monosaccharide-based sensor array, combined with ML, holds promise for differentiating drug-resistant subpopulations. The requirement of only 16 features makes this approach particularly suitable for the development of inexpensive and easy-to-use analytical kits.

While the current monosaccharide-based fluorescent sensor array, coupled with ensemble learning, demonstrates remarkable accuracy in phenotyping drug-resistant *P. aeruginosa* isolates, we acknowledge that directly analyzing complex clinical samples (*e.g.*, sputum or blood samples) remains a challenge. The intrinsic binding affinity between a single monosaccharide and a bacterial lectin is relatively low. To facilitate future clinical translation, we aim to construct multivalent glycoclusters on biocompatible materials to exponentially amplify the binding affinity through the ‘multivalent effect’. Furthermore, optimizing the AI algorithms to decouple specific lectin-binding signals from background interference in polymicrobial environments will be a key focus of our future work.

## Conclusions

We have developed a sensor array that consists of a series of eight glycoprobes. These glycoprobes, which use DPAC as the fluorescence reporter, exhibited ratiometric fluorescence changes upon selective lectin binding due to conformational adaptability of the fluorogen. The array also generated characteristic signals for different bacteria, and the resulting signals were used for ML-based analyses, giving rise to bacterial classification. Notably, we demonstrated that bacterial strains that express lectins including *P. aeruginosa* and *A. baumannii* could be better differentiated by this technique. In addition, *P. aeruginosa* variants with induced antibiotic resistance could also be differentiated from the drug-sensitive strain using array sensing, and the differentiation was relevant to the lectin expression of these strains. As such, this study indicates that targeting bacterial lectins is a viable strategy by which to design sensor arrays for bacterial classification. The developed AI-assisted differentiation protocol also offers a complementary tool to nucleic acid-sequencing techniques that require tedious laboratory efforts, facilitating point-of-care decisions for precision antimicrobial therapy against multidrug-resistant infections.

## Author contributions

Z.-F. Zhang performed experiments, analyzed data, and drafted the original manuscript. W.-Z. Gui performed experiments and analyzed data. Y.-F. Tang performed machine learning data analysis and optimized learning methods. H.-Q. Gan analyzed data. X.-L. Hu, T. D. James, S. Yang, Q. Liu and X.-P. He secured funding, supervised the research, and wrote and edited the manuscript. All authors have approved the final version of the manuscript.

## Conflicts of interest

The authors declare no competing financial interest.

## Supplementary Material

SC-OLF-D6SC00084C-s001

## Data Availability

The data supporting this article have been included as part of the supplementary information (SI). Supplementary information: the experimental section, synthetic procedures and characterizations (NMR and HRMS) of new compounds, photophysical properties (UV-Vis and fluorescence spectra), and details in machine learning performance metrics. See DOI: https://doi.org/10.1039/d6sc00084c.
