# Supplementary material for: Artificial intelligence-assisted phenotyping of drug-resistant bacteria using a monosaccharide-based fluorescent sensor array
Source: Chem Sci. 2026 May 19;17(25):12324–33. doi: 10.1039/d6sc00084c (PMC13186398; doi:10.1039/d6sc00084c)
Supplement: SC-017-D6SC00084C-s001 [file SC-017-D6SC00084C-s001.pdf]

Supplementary Information (SI) for Chemical Science.
This journal is © The Royal Society of Chemistry 2026

Supporting Information

Artificial intelligence-assisted phenotyping of drug-resistant bacteria using a monosaccharide-based fluorescent sensor array

Zhuo-Fan Zhang,+[a] Wen-Zhen Gui,+[a] Yi-Fan Tang,+[c] Hui-Qi Gan,[a] Xi-Le Hu,*[a] Tony D James,*[ef] Shengbing Yang,*[e] Qian Liu*[d] and Xiao-Peng He*[a][b]

[a] Key Laboratory for Advanced Materials and Joint International Research Laboratory of Precision Chemistry and Molecular Engineering, Feringa Nobel Prize Scientist Joint Research Centre, School of Chemistry and Molecular Engineering, East China University of Science & Technology, Shanghai 200237, China
E-mail: xlhu@ecust.edu.cn (X.-L. Hu), xphe@ecust.edu.cn (X.-P. He)

[b] National Center for Liver Cancer, the International Cooperation Laboratory on Signal Transduction, Eastern Hepatobiliary Surgery Hospital, Shanghai 200438, China

[c] Academy of Pharmacy, Xi'an-Jiaotong Liverpool University, Suzhou, 215123, China

[d] Department of Laboratory Medicine, Ren Ji Hospital, 160 Pujian Road, Shanghai 200127, China. E-mail: liuqian_rj@shsmu.edu.cn

[e] Shanghai Key Laboratory of Orthopedic Implants, Department of Orthopedic Surgery, Shanghai Ninth People’s Hospital, Shanghai Jiao Tong University School of Medicine, Shanghai 200011, China. shengbingyang@shusmu.edu.cn

[f] Department of Chemistry, University of Bath, Bath BA2 7AY, UK. E-mail: t.d.james@bath.ac.uk

[g] School of Chemistry and Chemical Engineering, Henan Normal University, Xinxiang 453007, China.

[+] These authors contributed equally

Content list

S1. Experimental section

S2. Additional figures

S3. Original spectra of new compounds

S4. Additional references

S1. Experimental section

General remarks. All chemicals and reagents were purchased with analytical grade unless otherwise noted. ^{1}H NMR spectra were recorded on a Bruker AM 400 MHz spectrometer using tetramethylsilane (TMS) as the internal reference. ^{13}C NMR spectra were acquired on either a Bruker AM 400 MHz or a 600 MHz spectrometer, with TMS as the internal reference, depending on sample availability and instrument access. UV-Vis absorption spectra were measured on a Varian Cary 500 UV-Vis spectrophotometer. Fluorescence emission spectra were measured on a Cary Eclipse Fluorescence spectrophotometer. Fluorescence imaging was performed using a Leica TCS-SP8 STED 3x microscope operated in confocal mode. The fluorescence of glycoprobes incorporated in the sensor array was measured using a multifunctional microplate reader (Thermo Fisher).

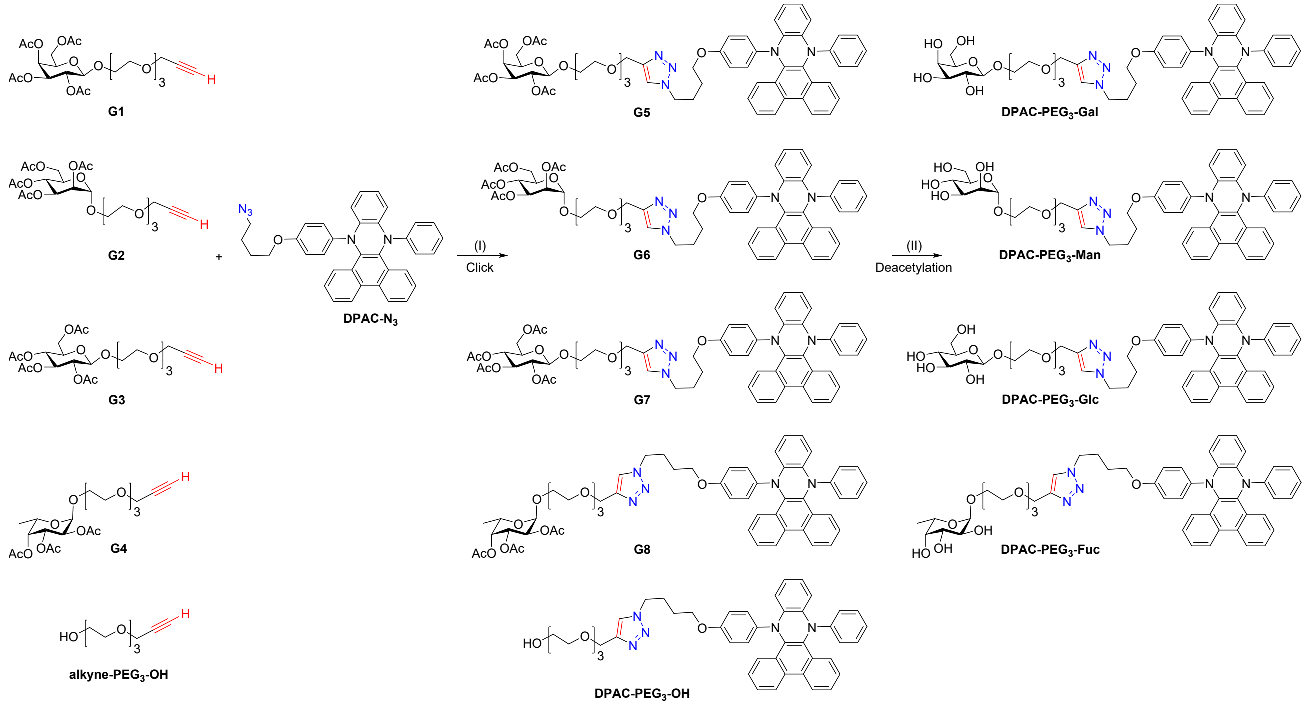

Scheme S1. Reagents and conditions: (I) $CuSO_4 \cdot 5H_2O$ and sodium ascorbate (VcNa) in MeOH at room temperature (r.t.); (II) CH_3OH/CH_3ONa in CH_3OH.

General procedure for click chemistry. To a mixture of alkyne substrates (**G1-G4**, propargyl alcohol, or alkyne-PEG$_3$-OH) and **DPAC-N$_3$** in dichloromethane (DCM)/MeOH (1:2, v/v), VcNa and $CuSO_4 \cdot 5H_2O$ were added sequentially. The resulting mixture was stirred at r.t. under an argon atmosphere for 6 h. Then, the mixture was diluted with DCM and washed with brine. The combined organic layer was dried over Na_2SO_4, filtered, concentrated in vacuum and purified by column chromatography on silica gel to afford **G5-G8**, **DPAC-OH**, and **DPAC-PEG$_3$-OH**, respectively.

G5: From **G1** (92 mg, 0.18 mmol), **DPAC-N$_3$** (88 mg, 0.16 mmol), VcNa (126 mg, 0.64 mmol) and $CuSO_4 \cdot 5H_2O$ (80 mg, 0.32 mmol), column chromatography over silica gel (DCM/MeOH = 20:1, v/v) afforded **G5** as a yellow solid (147 mg, 86%). R_f 0.50 (DCM/MeOH = 10:1, v/v). ^{1}H NMR (400 MHz, $CDCl_3$) δ 8.70 (d, J = 8.3 Hz, 2H),

8.14 (d, J = 7.5 Hz, 1H), 8.08 (d, J = 7.8 Hz, 1H), 7.73 – 7.65 (m, 2H), 7.60 (dt, J = 9.5, 4.0 Hz, 2H), 7.56 – 7.52 (m, 2H), 7.48 (t, J = 7.4 Hz, 1H), 7.29 (dt, J = 4.9, 3.7 Hz, 2H), 7.06 – 6.98 (m, 4H), 6.94 (d, J = 8.0 Hz, 2H), 6.79 (t, J = 7.2 Hz, 1H), 6.56 (d, J = 9.0 Hz, 2H), 5.39 – 5.37 (m, 1H), 5.28 (s, 1H), 5.20 (dd, J = 10.5, 8.0 Hz, 1H), 5.01 (dd, J = 10.5, 3.4 Hz, 1H), 4.65 (s, 2H), 4.57 (d, J = 8.0 Hz, 1H), 4.35 (t, J = 7.1 Hz, 2H), 4.19 – 4.09 (m, 2H), 3.97 – 3.89 (m, 2H), 3.78 (t, J = 6.0 Hz, 2H), 3.76 – 3.70 (m, 1H), 3.69 – 3.59 (m, 10H), 2.13 (s, 3H), 2.04 (s, 3H), 2.03 (s, 3H), 1.99 (d, J = 11.5 Hz, 5H), 1.71 – 1.63 (m, 2H); ^{13}C NMR (151 MHz, $CDCl_3$) δ 170.5, 170.3, 170.2, 169.6, 154.3, 148.5, 145.8, 145.1, 143.6, 141.8, 138.4, 136.8, 130.1, 129.6, 129.6, 129.3, 128.8, 127.6, 127.1, 126.8, 126.4, 126.3, 125.8, 125.7, 124.8, 124.6, 124.5, 123.1, 123.0, 122.6, 121.2, 120.7, 116.2, 114.8, 101.4, 71.0, 70.7, 70.7, 70.6, 70.6, 70.4, 69.7, 69.1, 68.9, 67.2, 67.1, 64.7, 61.4, 50.0, 27.2, 26.2, 20.8, 20.7(2), 20.7. HRMS (ESI, *m/z*): [M + Na]$^+$ calcd for $C_{59}H_{63}N_5O_{14}Na^+$ 1088.4269; found 1088.4258.

G6: From **G2** (92 mg, 0.18 mmol), **DPAC-N$_3$** (88 mg, 0.16 mmol), VcNa (126 mg, 0.64 mmol), and $CuSO_4·5H_2O$ (80 mg, 0.32 mmol), column chromatography over silica gel (DCM/MeOH = 20:1, v/v) afforded **G6** as a yellow solid (150 mg, 88%), R_f = 0.50 (DCM/MeOH = 10:1, v/v)；^{1}H NMR (400 MHz, $CDCl_3$) δ 8.64 (d, J = 8.3 Hz, 2H), 8.10 (dd, J = 8.1, 1.1 Hz, 1H), 8.04 (dd, J = 8.2, 0.9 Hz, 1H), 7.68 – 7.61 (m, 2H), 7.58 – 7.46 (m, 4H), 7.43 (t, J = 7.2 Hz, 1H), 7.27 – 7.21 (m, 2H), 7.01 – 6.95 (m, 4H), 6.91 (d, J = 7.9 Hz, 2H), 6.75 (t, J = 7.2 Hz, 1H), 6.54 – 6.49 (m, 2H), 5.34 (dd, J = 10.1, 3.3 Hz, 1H), 5.28 – 5.21 (m, 2H), 4.85 (d, J = 1.5 Hz, 1H), 4.62 (s, 2H), 4.31 – 4.24 (m, 3H), 4.06 (ddd, J = 7.7, 7.0, 2.3 Hz, 2H), 3.77 (dt, J = 9.0, 3.3 Hz, 1H), 3.69 (t, J = 5.9 Hz, 2H), 3.66 – 3.59 (m, 11H), 2.11 (s, 3H), 2.07 (s, 3H), 2.01 (s, 3H), 1.96 (s, 3H), 1.92 (dd, J = 14.9, 7.3 Hz, 2H), 1.63 – 1.54 (m, 2H). ^{13}C NMR (151 MHz, $CDCl_3$) δ 170.8, 170.1, 170.0, 169.8, 154.4, 148.5, 145.8, 145.2, 143.6, 141.8, 138.5, 136.8, 130.2, 129.7, 129.4, 128.8, 127.7, 127.1, 126.9, 126.44, 126.37, 125.8, 124.8, 124.6, 124.5, 123.1, 123.0, 122.6, 121.3, 120.7, 116.2, 114.8, 97.8, 70.8, 70.7, 70.6, 70.1, 69.8, 69.6, 69.2, 68.5, 67.5, 67.1, 66.2, 64.7, 62.5, 50.0, 27.3, 26.3, 21.0, 20.9, 20.83, 20.81. HRMS (ESI, *m/z*): [M + Na]$^+$ calcd for $C_{59}H_{63}N_5O_{14}Na^+$ 1088.4269; found, 1088.4247.

G7: From **G3** (92 mg, 0.18 mmol), **DPAC-N$_3$** (88 mg, 0.16 mmol), VcNa (126 mg, 0.64 mmol), and $CuSO_4·5H_2O$ (80 mg, 0.32 mmol), column chromatography over silica gel (DCM/MeOH = 20:1, v/v) afforded **G7** as a yellow solid (145 mg, 85%), R_f = 0.50 (DCM/MeOH = 10:1, v/v)；^{1}H NMR (400 MHz, $CDCl_3$) δ 8.66 (d, J = 8.3 Hz, 2H), 8.15 – 8.04 (m, 2H), 7.71 – 7.63 (m, 2H), 7.60 – 7.48 (m, 4H), 7.45 (t, J = 7.4 Hz, 1H), 7.29 – 7.22 (m, 2H), 7.02 (dd, J = 10.8, 5.4 Hz, 4H), 6.93 (d, J = 8.0 Hz, 2H), 6.77 (t, J = 7.2 Hz, 1H), 6.53 (t, J = 6.3 Hz, 2H), 5.21 (t, J = 9.5 Hz, 1H), 5.09 (t, J = 9.7 Hz, 1H), 4.99 (dd, J = 9.5, 8.1 Hz, 1H), 4.64 (s, 2H), 4.60 (d, J = 8.0 Hz, 1H), 4.32 – 4.22 (m, 3H), 4.12 (dd, J = 12.3, 2.2 Hz, 1H), 3.92 (dt, J = 11.0, 4.2 Hz, 1H), 3.75 – 3.58 (m, 14H), 2.06 (s, 3H), 2.03 (s, 3H), 2.01 (s, 3H), 1.99 (s, 3H), 1.94 (dd, J = 14.9, 7.3 Hz, 2H), 1.66 – 1.57 (m, 2H). ^{13}C NMR (151 MHz, $CDCl_3$) δ 170.6, 170.2, 169.4, 169.3, 154.3, 148.3, 145.6, 145.0, 143.4, 141.6, 138.3, 136.6, 130.0, 129.5, 129.2, 128.7, 127.4, 127.0, 126.7, 126.3, 126.2, 125.6, 124.7, 124.4, 124.3, 122.93, 122.88, 122.5, 121.2, 120.6, 116.1, 114.7, 100.7, 72.8, 71.7, 71.2, 70.6, 70.5, 70.4, 70.2, 69.6, 69.0, 68.3, 66.9, 64.5, 61.9, 49.8, 27.0, 26.1, 20.7, 20.62, 20.56, 20.5. HRMS (ESI, *m/z*): [M + Na]$^+$ calcd for $C_{59}H_{63}N_5O_{14}Na^+$ 1088.4269; found, 1088.4268.

G8: From **G4** (50 mg, 0.11 mmol), **DPAC-N$_3$** (60 mg, 0.11 mmol), VcNa (87 mg, 0.44 mmol), and $CuSO_4·5H_2O$ (55 mg, 0.22 mmol), The crude product was used directly in the next step without further purification.

DPAC-PEG$_3$-OH: From alkyne-PEG$_3$-OH (50 mg, 0.27 mmol), **DPAC-N$_3$** (145.5 mg, 0.27 mmol), VcNa (210.5 mg, 1.06 mmol) and $CuSO_4 \cdot 5H_2O$ (132.7 mg, 0.53 mmol), column chromatography over silica gel (DCM/MeOH = 20:1, v/v) afforded **DPAC-PEG$_3$-OH** as a yellow solid (180.6 mg, 91%). R_f = 0.32 (DCM/MeOH = 10:1, v/v); ^{1}H NMR (400 MHz, $CDCl_3$) δ 8.71 (d, J = 8.3 Hz, 2H), 8.11 (ddd, J = 25.3, 8.2, 1.4 Hz, 2H), 7.73 – 7.65 (m, 2H), 7.65 – 7.52 (m, 4H), 7.48 (ddd, J = 8.1, 6.9, 1.1 Hz, 1H), 7.32 – 7.27 (m, 2H), 7.06 – 6.98 (m, 4H), 6.94 (dq, J = 7.0, 1.4 Hz, 2H), 6.79 (tt, J = 7.1, 1.1 Hz, 1H), 6.62 – 6.53 (m, 2H), 4.66 (s, 2H), 4.36 (t, J = 7.1 Hz, 2H), 3.80 (t, J = 5.9 Hz, 2H), 3.72 – 3.60 (m, 11H), 3.60 – 3.54 (m, 2H), 2.09 – 1.95 (m, 3H), 1.70 (dt, J = 9.1, 6.0 Hz, 3H). ^{13}C NMR (101 MHz, $CDCl_3$) δ 154.29, 148.41, 145.75, 145.04, 143.53, 141.75, 138.42, 136.79, 130.11, 129.59, 129.30, 128.76, 127.56, 127.04, 126.77, 126.35, 126.26, 125.72, 125.64, 124.72, 124.52, 124.44, 122.99, 122.93, 122.60, 121.18, 120.66, 116.14, 114.79, 72.59, 70.56, 70.48, 70.23, 69.57, 67.08, 64.63, 61.66, 50.84, 49.97, 27.18, 26.19. [M + Na]$^+$ calcd for $C_{45}H_{45}O_5N_5Na^+$ 758.3313; found, 758.3316.

General procedure for deacetylation. To a solution of **G5**-**G8** in MeOH, a freshly prepared MeOH solution of sodium methoxide was added. The resulting mixture was stirred at r.t. under an argon atmosphere for 3 h. Then, Na^+ exchange resin (Amberlite IR 120 H^+) was added to the mixture for adjusting the pH to 7.0. Then, the ion exchange resin was removed by filtration. The organic solvent in the resulting mixture was evaporated to obtain the deacetylated glycoprobes.

DPAC-PEG$_3$-Gal: From **G5** (106 mg, 0.10 mmol), **MeONa**(22.4 mg, 0.4 mmol) afforded **DPAC-PEG$_3$-Gal** as a yellow solid (88 mg, 98%), R_f = 0.21 (DCM/MeOH = 10:1, v/v); ^{1}H NMR (400 MHz, DMSO-*d6*) *δ* 8.89 (d, J = 8.2 Hz, 2H), 8.06 (s, 1H), 7.99 – 7.93 (m, 2H), 7.86 – 7.78 (m, 2H), 7.66 (q, J = 7.2 Hz, 2H), 7.60 (t, J = 7.6 Hz, 1H), 7.54 (t, J = 7.6 Hz, 1H), 7.35 (dd, J = 5.5, 3.8 Hz, 2H), 7.11 – 7.06 (m, 4H), 6.94 (d, J = 7.9 Hz, 2H), 6.83 (t, J = 7.2 Hz, 1H), 6.66 (d, J = 9.1 Hz, 2H), 4.47 (s, 2H), 4.34 (t, J = 7.0 Hz, 2H), 4.07 (d, J = 7.0 Hz, 1H), 3.80 (dt, J = 10.2, 3.9 Hz, 3H), 3.62 (d, J = 2.0 Hz, 1H), 3.56 – 3.46 (m, 16H), 3.26 (td, J = 10.1, 6.2 Hz, 4H), 1.91 – 1.82 (m, 2H), 1.59 – 1.51 (m, 2H). ^{13}C NMR (151 MHz, DMSO-*d6*) *δ* 154.5, 147.9, 145.0, 143.9, 142.7, 140.9, 137.5, 135.6, 129.7, 129.2, 129.0, 128.5, 128.2, 127.3, 127.2, 127.1, 126.7, 126.6, 125.9, 125.3, 124.9, 123.9, 123.8, 123.60, 123.58, 121.7, 121.1, 116.4, 114.9, 103.0, 76.9, 76.7, 73.4, 70.0, 69.8, 69.7, 69.6, 68.9, 67.8, 66.8, 63.5, 61.1, 48.9, 26.5, 25.7. HRMS (ESI, *m/z*): [M + Na]$^+$ calcd for $C_{51}H_{55}N_5O_{10}Na^+$ 920.3847; found, 920.3859.

DPAC-PEG$_3$-Man: From **G6** (106 mg, 0.10 mmol), **MeONa**(22.4 mg, 0.4 mmol) afforded **DPAC-PEG$_3$-Man** as a yellow solid (86 mg, 96%), R_f = 0.21 (DCM/MeOH = 10:1, v/v); ^{1}H NMR (400 MHz, $CDCl_3$) *δ* 8.91 (d, J = 8.3 Hz, 2H), 8.08 (s, 1H), 7.97 (dd, J = 10.6, 8.3 Hz, 2H), 7.84 (ddd, J = 9.4, 6.4, 3.7 Hz, 2H), 7.72 – 7.60 (m, 3H), 7.55 (t, J = 7.7 Hz, 1H), 7.39 – 7.34 (m, 2H), 7.10 (dt, J = 7.4, 3.4 Hz, 4H), 6.95 (d, J = 8.1 Hz, 2H), 6.84 (t, J = 7.3 Hz, 1H), 6.68 (d, J = 9.1 Hz, 2H), 4.73 (t, J = 4.3 Hz, 2H), 4.62 (d, J = 0.7 Hz, 1H), 4.59 (d, J = 5.9 Hz, 1H), 4.49 – 4.43 (m, 3H), 4.35 (t, J = 7.0 Hz, 2H), 3.81 (t, J = 6.2 Hz, 2H), 3.68 – 3.61 (m, 2H), 3.58 (t, J = 3.6 Hz, 1H), 3.53 – 3.36 (m, 14H), 3.33 – 3.28 (m, 1H), 1.92 – 1.83 (m, 2H), 1.61 – 1.53 (m, 2H). ^{13}C NMR (151 MHz, DMSO-*d6*) *δ* 154.5, 147.9, 145.0, 143.9, 142.7, 140.9, 137.5, 135.6, 129.7, 129.2, 129.0, 128.5, 128.2, 127.3, 127.2, 127.1, 126.7, 126.6, 125.9, 125.3, 124.9, 123.9, 123.8, 123.6, 121.7, 121.1, 116.4, 114.9, 100.0, 73.9,

70.9, 70.3, 69.8, 69.73, 69.67, 69.5, 68.9, 67.0, 66.8, 65.7, 63.5, 61.3, 48.9, 26.5, 25.7. HRMS (ESI, *m/z*): $[M + Na]^+$ calcd for $C_{51}H_{55}N_5O_{10}Na^+$ 920.3847; found, 920.3837.

DPAC-PEG$_3$-Glc: From **G7** (106 mg, 0.10 mmol), **MeONa**(22.4 mg, 0.4 mmol） afforded **DPAC-PEG$_3$-Glc** as a yellow solid (89 mg, 99%) R_f = 0.21 (DCM/MeOH = 10:1, v/v)；^{1}H NMR (400 MHz, DMSO-*d6*) δ 8.89 (d, J = 8.2 Hz, 2H), 8.06 (s, 1H), 8.00 – 7.94 (m, 2H), 7.87 – 7.78 (m, 2H), 7.69 – 7.58 (m, 3H), 7.53 (dd, J = 11.3, 4.1 Hz, 1H), 7.35 (dt, J = 5.5, 4.1 Hz, 2H), 7.09 (dt, J = 8.7, 3.5 Hz, 4H), 6.97 – 6.92 (m, 2H), 6.83 (t, J = 7.2 Hz, 1H), 6.66 (t, J = 6.3 Hz, 2H), 5.00 (d, J = 4.9 Hz, 1H), 4.94 (d, J = 4.1 Hz, 1H), 4.91 (d, J = 4.5 Hz, 1H), 4.51 (t, J = 5.8 Hz, 1H), 4.47 (s, 2H), 4.34 (t, J = 7.0 Hz, 2H), 4.13 (d, J = 7.8 Hz, 1H), 3.85 (dt, J = 9.0, 3.6 Hz, 1H), 3.79 (t, J = 6.2 Hz, 2H), 3.68 – 3.63 (m, 1H), 3.58 – 3.46 (m, 12H), 3.15 – 3.01 (m, 3H), 2.94 (td, J = 8.4, 4.8 Hz, 1H), 1.91 – 1.82 (m, 2H), 1.60 – 1.51 (m, 2H). ^{13}C NMR (151 MHz, DMSO-*d6*) δ 154.5, 147.9, 145.0, 143.9, 142.7, 140.9, 137.5, 135.6, 129.7, 129.2, 129.0, 128.5, 128.2, 127.3, 127.2, 127.1, 126.7, 126.6, 125.9, 125.3, 124.9, 123.9, 123.8, 123.60, 123.58, 121.7, 121.1, 116.4, 114.9, 103.0, 76.9, 76.7, 73.4, 70.0, 69.8, 69.7, 69.6, 68.9, 67.8, 66.8, 63.5, 61.1, 48.9, 26.5, 25.7. HRMS (ESI, *m/z*): $[M + Na]^+$ calcd for $C_{51}H_{55}N_5O_{10}Na^+$ 920.3847; found, 920.3853.

DPAC-PEG$_3$-Fuc: From **G8** (101 mg, 0.10 mmol), **MeONa**(16.8 mg, 0.3 mmol） afforded **DPAC-PEG$_3$-Fuc** as a yellow solid (75 mg, 82%), R_f = 0.24 (DCM/MeOH = 10:1, v/v)^{1}H NMR (400 MHz, DMSO-d_6) δ 8.91 (d, J = 8.3 Hz, 2H), 8.07 (s, 1H), 8.03 – 7.94 (m, 2H), 7.84 (m, 2H), 7.74 – 7.60 (m, 3H), 7.56 (t, J = 7.6 Hz, 1H), 7.37 (dd, J = 6.0, 3.4 Hz, 2H), 7.11 (dd, J = 8.2, 6.0 Hz, 4H), 6.96 (d, J = 8.3 Hz, 2H), 6.84 (t, J = 7.3 Hz, 1H), 6.68 (d, J = 8.8 Hz, 2H), 5.32 (t, J = 4.9 Hz, 1H), 4.78 (d, J = 3.6 Hz, 1H), 4.62 (d, J = 4.4 Hz, 1H), 4.49 (s, 2H), 4.36 (dd, J = 8.6, 5.8 Hz, 3H), 4.07 (d, J = 6.7 Hz, 1H), 3.80 (m, 3H), 3.58 – 3.45 (m, 12H), 3.41 – 3.36 (m, 1H), 3.24 (t, J = 4.6 Hz, 2H), 1.99 (m, 2H), 1.89 (m, 2H), 1.57 (m, 2H), 1.10 (d, J = 6.4 Hz, 3H), 0.85 (t, J = 6.5 Hz, 2H). ^{13}C NMR (101 MHz, DMSO-d_6) δ 154.9, 148.4, 145.5, 144.3, 143.1, 141.4, 138.0, 136.0, 130.2, 129.6, 129.4, 129.0, 128.7, 127.8, 127.6, 127.5, 127.2, 127.1, 126.4, 125.8, 125.4, 124.6, 124.3, 124.1, 122.2, 121.6, 116.8, 115.3, 99.8, 72.1, 70.3, 70.3, 70.2, 70.1, 70.0, 69.4, 68.5, 67.3, 67.1, 66.3, 64.0, 49.4, 49.1, 31.8, 30.8, 30.3, 29.5, 29.2, 27.0, 26.1, 22.6, 17.0, 14.41.HRMS (ESI, *m/z*): $[M + Na]^+$ calcd for $C_{51}H_{55}O_9N_5Na^+$ 904.3892; found, 904.3902.

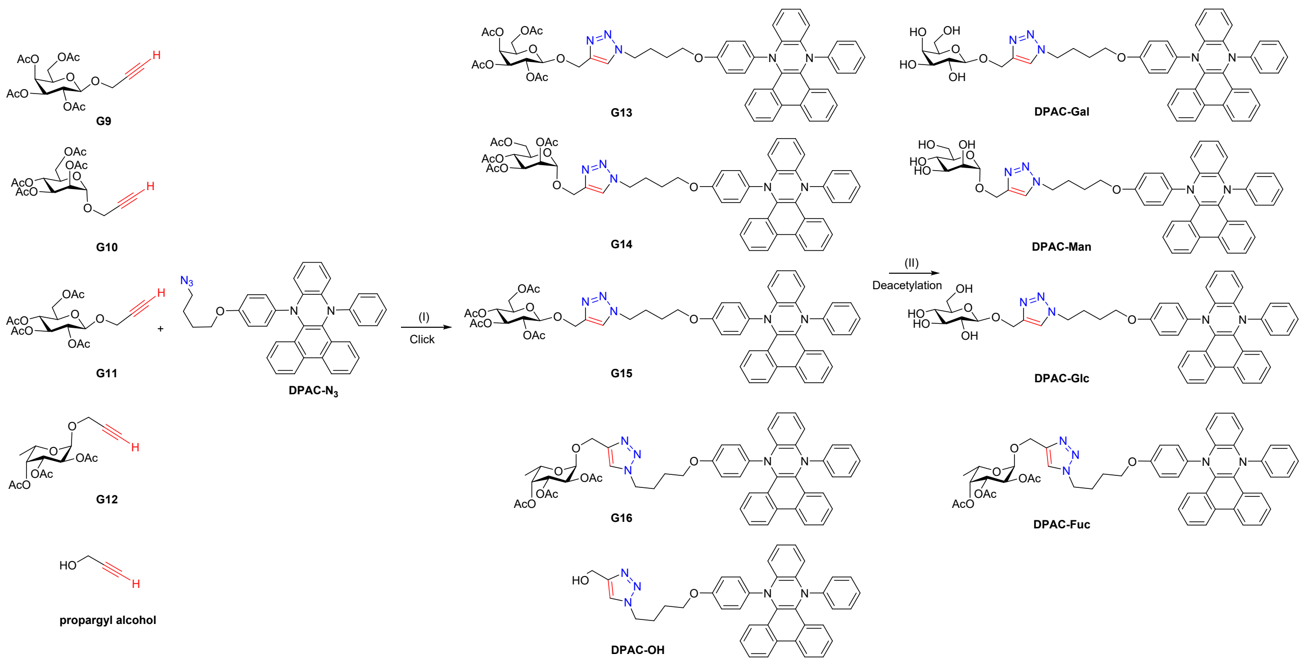

Scheme S2. Reagents and conditions: (I) $CuSO_4·5H_2O$ and VcNa in MeOH at r.t.; (II) CH_3OH/CH_3ONa in CH_3OH.

General procedure for click chemistry. To a mixture of **G9-G12** and **DPAC-N$_3$** in DCM/MeOH (1:2, v/v), VcNa and $CuSO_4·5H_2O$ were added sequentially. The resulting mixture was stirred at r.t. under an argon atmosphere for 6 h. Then, the mixture was diluted with DCM and washed with brine. The combined organic layer was dried over Na_2SO_4, filtered, concentrated in vacuum and purified by column chromatography on silica gel to afford **G13-G16**.

G13: From **G9** (77 mg, 0.20 mmol), **DPAC-N$_3$** (100 mg, 0.18 mmol), VcNa (143 mg, 0.72 mmol), and $CuSO_4·5H_2O$ (90 mg, 0.36 mmol), column chromatography over silica gel (DCM/MeOH = 20:1, v/v) afforded **G9** as a yellow solid (159 mg, 95%), R_f = 0.62 (DCM/MeOH = 10:1, v/v); ^{1}H NMR (400 MHz, $CDCl_3$) δ 8.71 (d, J = 7.6 Hz, 2H), 8.14 (d, J = 7.2 Hz, 1H), 8.08 (d, J = 7.7 Hz, 1H), 7.72 – 7.47 (m, 7H), 7.30 (s, 2H), 7.03 (s, 4H), 6.94 (d, J = 6.8 Hz, 2H), 6.84 – 6.75 (m, 1H), 6.57 (d, J = 7.7 Hz, 2H), 5.39 (d, J = 3.3 Hz, 1H), 5.22 (dd, J = 10.4, 8.0 Hz, 1H), 5.03 – 4.92 (m, 2H), 4.80 (d, J = 12.5 Hz, 1H), 4.64 (d, J = 8.0 Hz, 1H), 4.38 (t, J = 7.1 Hz, 2H), 4.14 (tt, J = 11.2, 5.5 Hz, 2H), 3.94 (t, J = 6.6 Hz, 1H), 3.82 (t, J = 5.2 Hz, 2H), 2.14 (s, 3H), 2.03 (d, J = 11.1 Hz, 5H), 1.98 (s, 3H), 1.96 (s, 3H), 1.74 – 1.65 (m, 2H). ^{13}C NMR (151 MHz, $CDCl_3$) δ 170.5, 170.3, 170.2, 169.6, 154.3, 148.5, 145.8, 143.7, 141.9, 138.5, 136.9, 130.2, 129.73, 129.70, 129.4, 128.9, 127.7, 127.2, 126.9, 126.5, 126.4, 125.9, 125.8, 124.9, 124.63, 124.56, 123.12, 123.06, 122.8, 121.2, 120.8, 116.3, 114.9, 100.6, 71.0, 68.9, 67.18, 67.16, 63.0, 61.4, 50.2, 27.4, 26.3, 20.9, 20.82, 20.79, 20.7. HRMS (ESI, m/z): $[M + Na]^+$ calcd for $C_{53}H_{51}N_5O_{11}Na^+$ 956.3483; found, 956.3491.

G14: From **G10** (77 mg, 0.20 mmol), **DPAC-N$_3$** (100 mg, 0.18 mmol), VcNa (143 mg, 0.72 mmol), and $CuSO_4·5H_2O$ (90 mg, 0.36 mmol), column chromatography over silica gel (DCM/MeOH = 20:1, v/v) afforded **G14** as a yellow solid (142 mg, 85%TLC: R_f = 0.62 (DCM/MeOH = 10:1, v/v); ^{1}H NMR (400 MHz, $CDCl_3$) δ

8.71 (d, *J* = 8.3 Hz, 2H), 8.17 – 8.06 (m, 2H), 7.69 (td, *J* = 7.0, 4.3 Hz, 2H), 7.65 – 7.53 (m, 4H), 7.48 (t, *J* = 7.6 Hz, 1H), 7.32 – 7.27 (m, 2H), 7.07 – 7.00 (m, 4H), 6.95 (d, *J* = 8.2 Hz, 2H), 6.80 (t, *J* = 7.2 Hz, 1H), 6.58 (d, *J* = 9.0 Hz, 2H), 5.32 (s, 1H), 5.28 (s, 1H), 5.24 (s, 1H), 4.95 (s, 1H), 4.83 (d, *J* = 12.3 Hz, 1H), 4.65 (d, *J* = 12.3 Hz, 1H), 4.38 (t, *J* = 7.1 Hz, 2H), 4.31 (dd, *J* = 1 2.1, 5.0 Hz, 1H), 4.14 – 4.05 (m , 2H), 3.81 (t, *J* = 5.8 Hz, 2H), 2.14 (s, 3H), 2.11 (s, 3H), 2.08 – 2.01 (m, 5H), 1.98 (s, 3H), 1.74 – 1.65 (m, 2H). ^{13}C NMR (151 MHz, $CDCl_3$) *δ* 170.8, 170.2, 170.0, 169.8, 154.4, 148.5, 145.9, 143.6, 141.9, 138.5, 136.9, 130.2, 129.7, 129.4, 128.9, 127.7, 127.2, 126.9, 126.5, 126.4, 125.83, 125.76, 124.8, 124.64, 124.55, 123.1, 123.0, 122.9, 121.3, 120.8, 116.3, 114.9, 97.0, 69.6, 69.2, 68.8, 67.2, 66.2, 62.5, 61.2, 50.2, 27.3, 26.3, 21.0, 20.9, 20.82, 20.81. HRMS (ESI, *m/z*): [M + Na]$^+$ calcd for $C_{53}H_{51}N_5O_{11}Na^+$ 956.3483; found, 956.3502.

G15: From **G11** (77 mg, 0.20 mmol), **DPAC-N$_3$** (100 mg, 0.18 mmol), VcNa (143 mg, 0.72 mmol), and $CuSO_4{\cdot}5H_2O$ (90 mg, 0.36 mmol), column chromatography over silica gel (DCM/MeOH = 20:1, v/v) afforded **G15** as a yellow solid (151 mg, 90%), R_f = 0.62 (DCM/MeOH = 10:1, v/v); ^{1}H NMR (400 MHz, $CDCl_3$) δ 8.72 (d, *J* = 8.2 Hz, 2H), 8.19 – 8.06 (m, 2H), 7.72 – 7.48 (m, 7H), 7.34 – 7.27 (m, 2H), 7.07 – 6.98 (m, 4H), 6.94 (d, *J* = 7.9 Hz, 2H), 6.80 (t, *J* = 6.9 Hz, 1H), 6.57 (d, *J* = 8.7 Hz, 2H), 5.19 (t, *J* = 9.4 H z, 1H), 5.09 (t, *J* = 9.6 Hz, 1H), 5.04 – 4.98 (m, 1H), 4.91 (d, *J* = 12.6 Hz, 1H), 4.80 (d, *J* = 12.6 Hz, 1H), 4.66 (d, *J* = 7.9 Hz, 1H), 4.38 (t, *J* = 7.1 Hz, 2H), 4.26 (dd, *J* = 12.3, 4.7 Hz, 1H), 4.14 (dd, *J* = 12.3, 2.2 Hz, 1H), 3.82 (t, *J* = 5.7 Hz, 2H), 3.72 (m, 1H), 2.07 (s, 3H), 2.06 – 2.01 (m, 5H), 1.99 (s, 3H), 1.95 (s, 3H), 1.72 – 1.68 (m, 2H). ^{13}C NMR (151 MHz, $CDCl_3$) *δ* 170.8, 170.3, 169.6, 169.5, 154.3, 148.5, 145.9, 144.2, 143.7, 141.9, 138.5, 136.9, 130.2, 129.74, 129.71, 129.4, 128.9, 127.7, 127.2, 126.9, 126.5, 126.4, 125.9, 125.8, 124.9, 124.64, 124.57, 123.12, 123.06, 122.9, 121.3, 120.8, 116.2, 114.9, 100.0, 72.9, 72.0, 71.3, 68.4, 67.1, 63.1, 61.9, 50.2, 27.4, 26.3, 20.9, 20.8, 20.8. HRMS (ESI, *m/z*): [M + Na]$^+$ calcd for $C_{53}H_{51}N_5O_{11}Na^+$ 956.3483; found, 956.3478.

G16: From **G12** (33 mg, 0.10 mmol), **DPAC-N$_3$** (55mg, 0.10 mmol), VcNa (79 mg, 0.4 mmol), and $CuSO_4{\cdot}5H_2O$ (50 mg, 0.20 mmol), column chromatography over silica gel (DCM/MeOH = 20:1, v/v) afforded **G16** as a yellow solid (80mg, 91%TLC: *Rf* = 0.67 (DCM/MeOH = 10:1, v/v); ^{1}H NMR (400 MHz, $CDCl_3$) *δ* 8.72 (d, *J* = 8.3 Hz, 2H), 8.19 – 8.11 (m, 1H), 8.08 (d, *J* = 8.2 Hz, 1H), 7.69 (td, *J* = 6.3, 3.6 Hz, 2H), 7.67 – 7.53 (m, 3H), 7.53 – 7.46 (m, 2H), 7.35 – 7.28 (m, 2H), 7.08 – 6.99 (m, 4H), 6.94 (d, *J* = 8.2 Hz, 2H), 6.80 (t, *J* = 7.2 Hz, 1H), 6.62 – 6.54 (m, 2H), 5.35 (dd, *J* = 10.7, 3.3 Hz, 1H), 5.31 – 5.26 (m, 1H), 5.20 – 5.10 (m, 2H), 4.81 (d, *J* = 12.5 Hz, 1H), 4.62 (d, *J* = 12.4 Hz, 1H), 4.39 (t, *J* = 7.2 Hz, 2H), 4.19 (q, *J* = 6.5 Hz, 1H), 3.83 (t, *J* = 5.9 Hz, 2H), 2.16 (s, 3H), 2.11 – 2.03 (m, 2H), 2.00 (s, 3H), 1.97 (s, 3H), 1.72 (p, *J* = 6.0 Hz, 2H), 1.13 (d, *J* = 6.6 Hz, 3H). ^{13}C NMR (101 MHz, $CDCl_3$) *δ* 170.6, 170.4, 170.1, 154.3, 148.4, 145.8, 144.0, 143.6, 141.8, 138.4, 136.8, 130.1, 129.6, 129.3, 128.8, 127.6, 127.0, 126.8, 126.4, 126.3, 125.8, 125.6, 124.7, 124.5, 124.4, 123.0, 122.9, 122.5, 121.2, 120.7, 116.1, 114.8, 95.6, 71.2, 68.0, 68.0, 67.1, 64.7, 61.3, 53.4, 50.00, 27.3, 26.2, 20.8, 20.7, 20.7, 15.9. HRMS (ESI, *m/z*): [M + H]$^+$ calcd for $C_{51}H_{50}O_9N_5^+$ 876.3603; found, 876.3609.

DPAC-OH: From propargyl alcohol (5 mg, 0.09 mmol), **DPAC-N$_3$** (49 mg, 0.09 mmol), VcNa (71 mg, 0.36 mmol) and $CuSO_4{\cdot}5H_2O$ (44 mg, 0.18 mmol), column chromatography over silica gel (DCM/MeOH = 20:1, v/v) afforded **DPAC-OH** as a yellow solid (45.6 mg, 84%). R_f = 0.43 (DCM/MeOH = 20:1, v/v); ^{1}H NMR (400 MHz, $CDCl_3$) δ 8.72 (d, *J* = 8.3 Hz, 2H), 8.11 (dd, *J* = 24.6, 8.1 Hz, 2H), 7.74 – 7.43 (m, 7H), 7.30 (d, *J* = 4.9 Hz, 2H),

7.09 – 6.98 (m, 4H), 6.94 (d, *J* = 8.2 Hz, 2H), 6.79 (t, *J* = 7.2 Hz, 1H), 6.62 – 6.53 (m, 2H), 4.75 (s, 2H), 4.38 (t, *J* = 7.1 Hz, 2H), 3.80 (t, *J* = 5.9 Hz, 2H), 2.96 (s, 1H), 2.88 (d, *J* = 0.6 Hz, 1H). ^{13}C NMR (101 MHz, $CDCl_3$) δ 129.29, 128.75, 127.55, 127.03, 126.76, 126.35, 125.76, 125.64, 124.74, 124.43, 122.99, 122.93, 121.08, 120.64, 116.13, 114.78, 67.05, 50.77, 50.14, 27.15, 26.16. HRMS (ESI, *m/z*): [M + H]$^+$ calcd for $C_{39}H_{34}O_2N_5^+$ 604.2707; found, 604.2698.

General procedure for deacetylation. To a solution of **G13**-**G16** in MeOH and freshly prepared MeOH solution of sodium methoxide was added. The resulting mixture was stirred at r.t. under an argon atmosphere for 3 h. then Na^+ exchange resin (Amberlite IR 120 H^+) was added to the mixture for adjusting the pH to 7.0. Then, the ion exchange resin was removed by filtration. The organic solvent in the resulting mixture was evaporated to obtain the deacetylated glycoprobes.

DPAC-Gal: From **G13** (93 mg, 0.10 mmol), **MeONa**(22.4 mg, 0.4 mmol) afforded **DPAC-Gal** as a yellow solid (73 mg, 97%), R_f = 0.34 (DCM: MeOH = 10:1, v/v); ^{1}H NMR (400 MHz, DMSO-*d6*) *δ* 8.90 (d, J = 8.3 Hz, 2H), 8.09 (s, 1H), 8.01 – 7.95 (m, 2H), 7.88 – 7.78 (m, 2H), 7.70 – 7.59 (m, 3H), 7.55 (t, *J* = 7.6 Hz, 1H), 7.38 – 7.32 (m, 2H), 7.09 (dd, *J* = 8.0, 5.4 Hz, 4H), 6.95 (d, *J* = 8.0 Hz, 2H), 6.84 (t, *J* = 7.2 Hz, 1H), 6.68 (d, *J* = 9.0 Hz, 2H), 4.80 (d, *J* = 12.1 Hz, 1H), 4.59 (d, *J* = 12.1 Hz, 1H), 4.35 (t, *J* = 7.0 Hz, 3H), 4.19 (d, *J* = 7.0 Hz, 1H), 3.81 (t, *J* = 6.0 Hz, 2H), 3.63 (s, 1H), 3.57 – 3.49 (m, 2H), 3.36 (t, *J* = 6.0 Hz, 1H), 3.32 – 3.24 (m, 2H), 3.17 (s, 2H), 1.92 – 1.84 (m, 2H), 1.62 – 1.54 (m, 2H). ^{13}C NMR (151 MHz, DMSO-*d6*) *δ* 154.5, 147.9, 145.0, 143.8, 142.7, 140.9, 137.5, 135.6, 129.7, 129.1, 129.0, 128.5, 128.2, 127.3, 127.2, 127.1, 126.7, 126.6, 125.9, 125.3, 124.9, 124.1, 123.9, 123.59, 123.57, 121.7, 121.1, 116.4, 114.9, 102.7, 75.3, 73.4, 70.5, 68.2, 66.8, 61.4, 60.5, 48.9, 26.5, 25.7. HRMS (ESI, *m/z*): [M + Na]$^+$ calcd for $C_{45}H_{43}N_5O_7Na^+$ 788.3060; found, 788.3067.

DPAC-Man: From **G14** (93 mg, 0.10 mmol), **MeONa**(22.4 mg, 0.4 mmol) afforded **DPAC-Man** as a yellow solid (75 mg, 98%), R_f = 0.34 (DCM/MeOH = 10 : 1, v/v); ^{1}H NMR (400 MHz, DMSO-*d6*) *δ* 8.91 (d, *J* = 8.3 Hz, 2H), 8.11 (s, 1H), 8.03 – 7.94 (m, 2H), 7.90 – 7.80 (m, 2H), 7.72 – 7.60 (m, 3H), 7.56 (t, *J* = 7.6 Hz, 1H), 7.42 – 7.34 (m, 2H), 7.10 (dd, *J* = 7.9, 5.7 Hz, 4H), 6.96 (d, *J* = 8.2 Hz, 2H), 6.85 (t, *J* = 7.2 Hz, 1H), 6.69 (d, *J* = 8.9 Hz, 2H), 4.84 (m, 4H), 4.70 (s, 1H), 4.64 (d, *J* = 12.4 Hz, 1H), 4.49 (d, *J* = 12.2 Hz, 1H), 4.36 (t, *J* = 7.0 Hz, 2H), 4.20 (m, 1H), 3.82 (t, *J* = 6.1 Hz, 2H), 3.70 – 3.65 (m, 1H), 3.58 (d, *J* = 16.0 Hz, 2H), 3.48 (s, 1H), 3.31 – 3.20 (m, 1H), 1.93 – 1.85 (m, 2H), 1.62 – 1.53 (m, 2H). ^{13}C NMR (151 MHz, DMSO-*d6*) *δ* 154.5, 147.9, 145.0, 143.4, 142.7, 140.9, 137.5, 135.6, 129.7, 129.2, 129.0, 128.5, 128.2, 127.3, 127.2, 127.1, 126.7, 126.6, 125.9, 125.3, 124.9, 124.0, 123.9, 123.6, 121.7, 121.1, 116.3, 114.9, 99.0, 74.2, 70.9, 70.2, 67.0, 66.8, 61.3, 59.1, 49.0, 26.5, 25.7. HRMS (ESI, *m/z*): [M + Na]$^+$ calcd for $C_{45}H_{43}N_5O_7Na^+$ 788.3060; found, 788.3052.

DPAC-Glc: From **G15** (93 mg, 0.10 mmol), **MeONa**(22.4 mg, 0.4 mmol) afforded **DPAC-Glc** as a yellow solid (75 mg, 98%), R_f = 0.34 (DCM/MeOH = 10 : 1, v/v); ^{1}H NMR (400 MHz, DMSO-*d6*) *δ* 8.90 (d, *J* = 8.2 Hz, 2H), 8.11 (s, 1H), 8.02 – 7.95 (m, 2H), 7.88 – 7.79 (m, 2H), 7.71 – 7.59 (m, 3H), 7.55 (t, *J* = 7.6 Hz, 1H), 7.40 – 7.33 (m, 2H), 7.10 (dd, *J* = 8.1, 6.8 Hz, 4H), 6.96 (d, *J* = 7.9 Hz, 2H), 6.84 (t, *J* = 7.2 Hz, 1H), 6.68 (d, *J* = 9.1 Hz, 2H), 5.03 (m, 2H), 4.87 – 4.81 (m, 1H), 4.62 (d, *J* = 12.1 Hz, 1H), 4.36 (t, *J* = 7.0 Hz, 2H), 4.25 (d, *J* = 7.8 Hz, 1H), 3.81 (t, *J* = 6.2 Hz, 2H), 3.72 (d, *J* = 10.7 Hz, 1H), 3.50 – 3.43 (m, 2H), 3.19 – 3.03 (m, 4H), 2.98 (t, *J* = 8.4

Hz, 1H), 1.94 – 1.83 (m, 2H), 1.63 – 1.53 (m, 2H). ^{13}C NMR (151 MHz, DMSO-*d6*) *δ* 154.6, 148.0, 145.1, 143.9, 142.8, 141.0, 137.6, 135.7, 129.8, 129.3, 129.1, 128.6, 128.3, 127.5, 127.3, 127.2, 126.9, 126.8, 126.1, 125.4, 125.1, 124.3, 124.0, 123.70, 123.68, 121.8, 121.2, 116.5, 115.0, 102.2, 77.0, 76.8, 73.5, 70.2, 67.0, 61.7, 61.3, 49.1, 26.6, 25.8.HRMS (ESI, *m/z*): [M + Na]$^+$ calcd for $C_{45}H_{43}N_5O_7Na^+$ 788.3060; found, 788.3045.

DPAC-Fuc: From **G16** (88 mg, 0.10 mmol), **MeONa**(16.8 mg, 0.3 mmol ）afforded **DPAC-Fuc** as a yellow solid 67mg, 89%), R_f = 0.39 (DCM/MeOH = 10 : 1, v/v)；^{1}H NMR (400 MHz, DMSO-*d6*) *δ* 8.91 (d, *J* = 8.3 Hz, 2H), 8.09 (s, 1H), 7.98 (dd, *J* = 10.6, 8.4 Hz, 2H), 7.84 (ddd, *J* = 15.8, 6.3, 3.7 Hz, 2H), 7.74 – 7.60 (m, 3H), 7.56 (t, *J* = 7.6 Hz, 1H), 7.37 (dd, *J* = 6.0, 3.4 Hz, 2H), 7.11 (dt, *J* = 9.1, 3.5 Hz, 4H), 6.96 (d, *J* = 8.2 Hz, 2H), 6.84 (t, *J* = 7.2 Hz, 1H), 6.71 – 6.63 (m, 2H), 5.76 (s, 1H), 4.71 (d, *J* = 3.2 Hz, 1H), 4.60 (d, *J* = 12.2 Hz, 1H), 4.52 – 4.44 (m, 2H), 4.41 (t, *J* = 5.1 Hz, 2H), 4.36 (t, *J* = 7.1 Hz, 2H), 3.80 (m, 3H), 3.53 (m, 2H), 3.47 (m, 3.9 Hz, 1H), 1.89 (m, 2H), 1.59 (t, *J* = 7.5 Hz, 2H), 1.06 (d, *J* = 6.5 Hz, 3H).^{13}C NMR (101 MHz, DMSO-d_6) *δ* 155.0, 148.4, 145.5, 144.4, 143.2, 141.4, 136.0, 130.2, 129.6, 129.4, 129.0, 128.7, 127.8, 127.7, 127.5, 127.2, 127.1, 126.4, 125.8, 125.4, 124.4, 124.2, 124.1, 122.2, 121.6, 116.8, 115.3, 99.1, 72.0, 70.0, 68.4, 67.3, 66.5, 60.7, 55.4, 49.4, 27.0, 26.2, 16.9. HRMS (ESI, *m/z*): [M + Na]$^+$ calcd for $C_{45}H_{43}N_5O_6Na_+$772.3111; found, 772.3109

Fluorescence spectroscopy. A DMSO solution of the glycoprobes (1 mM) was diluted into phosphate-buffered saline (PBS) containing 0.02% (v/v) Tween 20 to afford a final concentration of 10 μM. The fluorescence spectra of the resuting solutions were recorded on a Cary Eclipse Fluorescence spectrophotometer with excitation at 360 nm, using gratings of 1200 lines/mm and a PMT voltage of 700 V.

UV-Vis spectroscopy. A DMSO solution of the glycoprobes (1 mM) was diluted into PBS containing 0.02% (v/v) Tween 20 to afford a final concentration of 10 μM. The UV-Vis spectra of the resulting solutions were recorded on a Varian Cary 500 UV-Vis spectrophotometer.

Bacterial strains and culture. *Pseudomonas aeruginosa* (*P. aeruginosa*, ATCC 27853), *Acinetobacter baumannii* (*A. baumannii*, ATCC 19606), *Escherichia coli* (*E. coli*, ATCC 35218), *Escherichia coli* (*E. coli*, ATCC 25922), *Klebsiella pneumoniae* (*K. pneumonia*, ATCC 13883), Methicillin-resistant *Staphylococcus aureus* (MRSA, ATCC 43300), methicillin-sensitive *Staphylococcus aureus* (MSSA, ATCC 29213), *Staphylococcus epidermidis* (*S. epidermidis*, CMCC 26069), *Streptococcus* (CMCC 32206) were obtained from ATCC (American Type Culture Collection). *P. aeruginosa*-3887 and *P. aeruginosa*-A258 were obtained from the Renji Hospital affiliated with the Jiaotong University in Shanghai. All isolates on Luria-Bertani (LB) agar plates were transferred to LB culture medium, and then grown at 37 ℃ with shaking for 12 h.

Minimum inhibitory concentration (MIC). MIC values were measured according to the Clinical and Laboratory Standards Institute (CLSI) standard using Luria-Bertani (LB) broth. Antibiotics including ceftazidime (CAZ), gentamicin (GEN), levofloxacin (LEV), polymyxin B (PMB) and tetracycline hydrochloride (TCH) were added to a 96-well microtitre plate. Each antibiotic was diluted using a two-fold dilution pattern and wells containing different concentrations (0.0625, 0.125, 0.25, 0.5, 1.0, 2.0, 4.0, 8.0, 16.0, 32.0, 64.0 and 128 μg mL^{-1}) were

prepared. The inoculum was prepared by suspending several colonies of bacteria in LB broth to a final bacterial concentration of 1 × 10^6 CFU mL^{-1} per well. The plates were covered and incubated at 37 ℃ for 18-20 h. The lowest drug concentration that prevents the appearance of turbidity was defined as the MIC.[1]

Induction of drug resistance. The drug-resistance of *P. aeruginosa* (ATCC 27853) was induced according to previous literature methods.[2] Bacteria were inoculated into the LB broth with serum, and incubated at 37 ℃ until logarithmic growth phase. A half-MIC was used as the initial inducing concentration, and then the antibiotic concentration was gradually increased by two times until the MIC of the test strain rose to the resistance range. Before moving to the next concentration, bacterial strains at each concentration were cultured for four passages. The last-passage isolates from each concentration were cryopreserved in glycerol containing (40 %) LB at -80 ℃.

Lü's alkaline methylene blue staining. *P. aeruginosa* (ATCC 27853) treated with **DPAC-PEG$_3$-Gal**, **PAC-PEG$_3$-Glc** or an equal volume of DMSO was added dropwise to the center of a pretreated slide, and a uniform smear was prepared with a sterile coating rod. The slide was placed at 3–5 cm above the alcohol lamp, and then fixed with a fire-baked slide. After the slide was cooled, an appropriate amount of Lü's basic methylene blue staining solution was added to cover the smear area, standing at r.t. for 3 min, and then rinsed slowly with deionized water along the back edge of the slide until the washing solution was colorless. After the edge water was absorbed by the absorbent paper, it was dried in a 55 ℃ incubator for 10 min. The bacterial morphology was imaged using an Olympus BX53 optical microscope with a 100× oil immersion objective.

Fluorescent sensor array analysis. The glycoprobes including **DPAC-Gal**, **DPAC-PEG$_3$-Gal**, **DPAC-Fuc**, **DPAC-PEG$_3$-Fuc**, **DPAC-Man**, **DPAC-PEG$_3$-Man**, **DPAC-Glc** and **DPAC-PEG$_3$-Glc** were diluted in PBS (0.01 M, pH 7.4) containing 0.02% (v/v) Tween 20 and centrifuged (220 rpm) at 37 ℃ for 30 min. Then, 190 μL of each glycoprobe solution was added to a black 96-well plate. Bacteria harvested from the liquid culture were thoroughly washed and subsequently diluted to prepare a suspension with an optical density (OD_{600} = 1). For analysis of bacteria, 10 μL suspension of each bacterium was added to the solution of glycoprobes in the black 96-well plates (6 replicates each). After centrifugation (400 rpm, 37 ℃), the fluorescence of each well was recorded in a high-throughput manner by a multifunctional microplate reader at 470 nm (blue channel) and 600 nm (red channel) with an excitation wavelength of 360 nm every 10 min for a total of 60 min.

Bacterial imaging by confocal fluorescence microscopy. *P. aeruginosa* (ATCC 27853) was centrifuged at 8000 rpm for 5 min, and then the supernatant was removed. Bacteria were re-suspended in PBS (0.01 M, pH 7.4) containing 0.02% (v/v) Tween 20. Prior to imaging, a bacterial suspension was incubated with a probe (**DPAC-Gal**, **DPAC-PEG$_3$-Gal**, **DPAC-OH** or **DPAC-PEG$_3$-OH**; 20 μM) for 60 min, and then with the commercial nucleic acid staining agent **Syto-9** (2.5 μM) for another 15 min in the dark. After incubation, the bacteria were washed three times with PBS (0.01 M, pH 7.4) by repeated centrifugation (8000 rpm, 5 min) and resuspension cycles to remove any unbound dyes. Finally, the bacteria were re-suspended in PBS (0.01 M, pH 7.4) containing 0.02% (v/v) Tween 20, and 2 μL of the suspension was placed onto a clean microscope slide. STED images were recorded on a Leica TCS-SP8 STED 3x super-resolution microscope equipped with a 100× oil-immersion

objective lens. The excitation and emission channels used for **DPAC** were 405 nm and 420-500/570-650 nm (blue/red emission), respectively.

Quantitative real-time polymerase chain reaction (PCR). Total RNA was isolated from biological replicates using the Trizol method according to the manufacturer's instructions. The samples were treated with Trizol (Accurate Biotechnology), followed by phenol-chloroform-isoamyl alcohol extraction and ethanol precipitation. RNA was first reverse transcribed to cDNA using the ABScript III RT Master Mix (ABdonal). Then, PCR was performed on a CFX384 qPCR cycler (Bio-Rad) using a SYBR Green Fast qPCR Mix (ABdonal) and the following primers:

Nucleotide sequence (5' to 3')

lecA-F: GGGTTGCACCCAATAATGTC

lecA-R: CCAATATTGACACTGAACGA

lecB-F: GCTGACCTGGACCTGTACCT

lecB-R: GCACCAATAACGCCGTCATC

rpoS-F: CTCCCCGGGCAACTCCAAAAG

rpoS-R: CGATCATCCGCTTCCGACCAG

Melting curve analyses were carried out immediately after amplification to verify the specificity of the PCR amplification products. Fluorescence was measured at the end of the annealing extension phase of each cycle. A threshold value for the fluorescence of all samples was set manually. The reaction cycle at which the PCR product exceeds this fluorescence threshold was identified as the threshold cycle (CT). The relative mRNA expression level was determined by the $2^{-\Delta\Delta Ct}$ method.

Machine learning methods and data analysis

Software Implementation and Computational Environment. All machine learning analyses and performance evaluations were conducted in Python 3.13.5 within the VSCode environment. The following packages and their respective functions were employed.

sklearn.feature_selection: Recursive Feature Elimination (RFE) for feature selection; VarianceThreshold for filtering low-variance features

sklearn.linear_model: ElasticNet, Lasso, and Ridge classifiers

sklearn.neighbors: K-nearest neighbors (KNN) non-parametric classification via KNeighborsClassifier

sklearn.ensemble: RandomForestClassifier for classification based on ensemble decision trees

sklearn.svm: Support Vector Machine (SVM) classifier

lightgbm and xgboost: LightGBM and XGBoost gradient boosting frameworks

sklearn.model_selection: train_test_split for training/test set partitioning; StratifiedKFold for stratified cross-validation; cross_val_predict for generating out-of-fold (OOF) probabilities from base learners

sklearn.base: clone function for independent estimator replication, ensuring cross-validation isolation and independent model fitting

matplotlib.pyplot: Comprehensive visualization of classification error rates, feature importance rankings, and model performance metrics

sklearn.metrics: Calculation of key performance metrics, including macro-average F1 score, accuracy, and

full classification report

statsmodels: variance_inflation_factor function (from statsmodels.stats.outliers_influence) for quantitative assessment of multicollinearity via variance inflation factor analysis

collections: Counter for majority voting in soft-voting ensemble

sklearn.pipeline: Building feature selector-classifier integrated workflow pipelines for reproducible preprocessing-modeling chains

sklearn.preprocessing: StandardScaler for data standardization; combined with sklearn.compose.ColumnTransformer for selective column processing and transformation

numpy: Meta-feature array operations and concatenation (np.hstack, np.concatenate) for implementing stacking ensemble learning

sklearn.decomposition: Principal Component Analysis (PCA) for dimensionality reduction and visualization of meta-learner probability outputs in low-dimensional feature spaces

Implementation of Multi-Bacteria Discrimination Models. Principal component analysis (PCA) was initially employed as an unsupervised screening method to cluster the 11 bacterial strains based on the extracted features. For machine learning modeling, the dataset was partitioned into 80% training and 20% test sets using stratified sampling, with five random repetitions of train_test_split to ensure robustness. All features were standardized using StandardScaler prior to model training. Training set performances were evaluated via 5-fold cross-validation.

A three-level data fusion strategy was implemented to enhance classification performance as follows.

Low-level fusion: Temporal data from all eight probes were concatenated, transforming dual-signal time-course measurements into a comprehensive 96-feature dataset.

Mid-level fusion: Feature refinement was achieved through recursive feature elimination (RFE) combined with variance inflation factor (VIF) analysis, selecting optimal feature subsets from different temporal segments.

High-level fusion: Decision-level integration was performed using ensemble methods (soft-voting and stacking) to combine predictions from multiple optimized models.

Eight multi-class classification algorithms were systematically compared: ElasticNet, KNN, Lasso, LightGBM, Random Forest, Ridge, SVM, and XGBoost. Hyperparameter optimization was conducted *via* grid search with 5-fold stratified cross-validation, tuning parameters including: regularization strength (C) for Lasso, Ridge, and SVM; C and l1_ratio for ElasticNet; number of estimators for tree-based methods; and number of neighbors for KNN.

In soft-voting ensemble, class probabilities from individual models were aggregated and the final prediction was determined by argmax of the averaged probabilities. For stacking ensemble, OOF prediction probabilities from base learners were horizontally concatenated to form meta-features, which were then used to train multiple meta-learners. The final model performance at each fusion level was comprehensively evaluated using macro F1 scores and classification error rates on both training and test sets.

S2. Additional figures

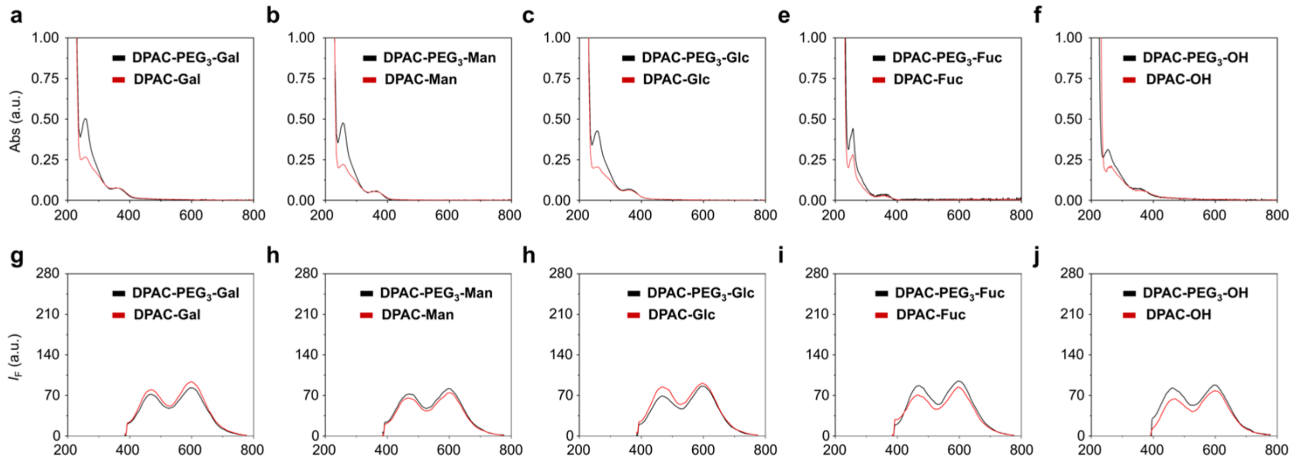

Figure S1. UV-Vis absorption spectra (a-e) and fluorescence emission spectra (f-j) of glycoprobes and aglycone control probes (10 μM) in PBS (0.01 M, pH 7.4) containing 0.02% (v/v) Tween 20

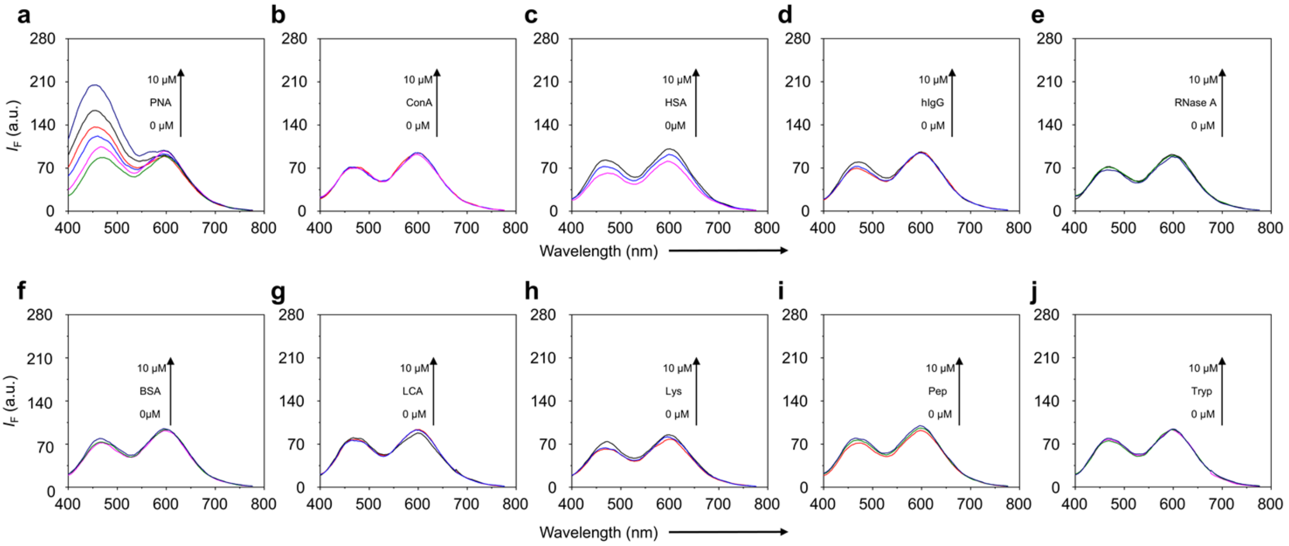

Figure. S2. Concentration-dependent fluorescence changes of **DPAC-Gal** (10 μM) in the presence of different proteins (as labeled, 0-10 μM, interval 2 μM) in PBS (0.01 M, pH 7.4) containing 0.02% (v/v) Tween 20; λ_{ex} = 360 nm.

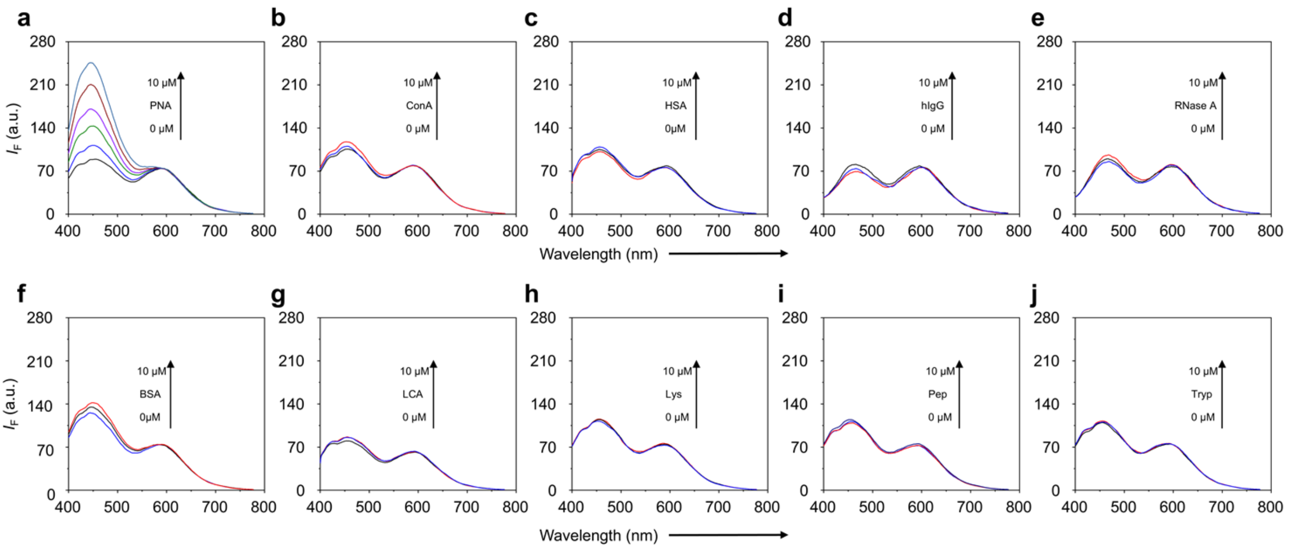

Figure. S3. Concentration-dependent fluorescence changes of **DPAC-PEG$_3$-Gal** (10 μM) in the presence of different proteins (as labeled, 0-10 μM, interval 2 μM) in PBS (0.01 M, pH 7.4) containing 0.02% (v/v) Tween 20; λ_{ex} = 360 nm.

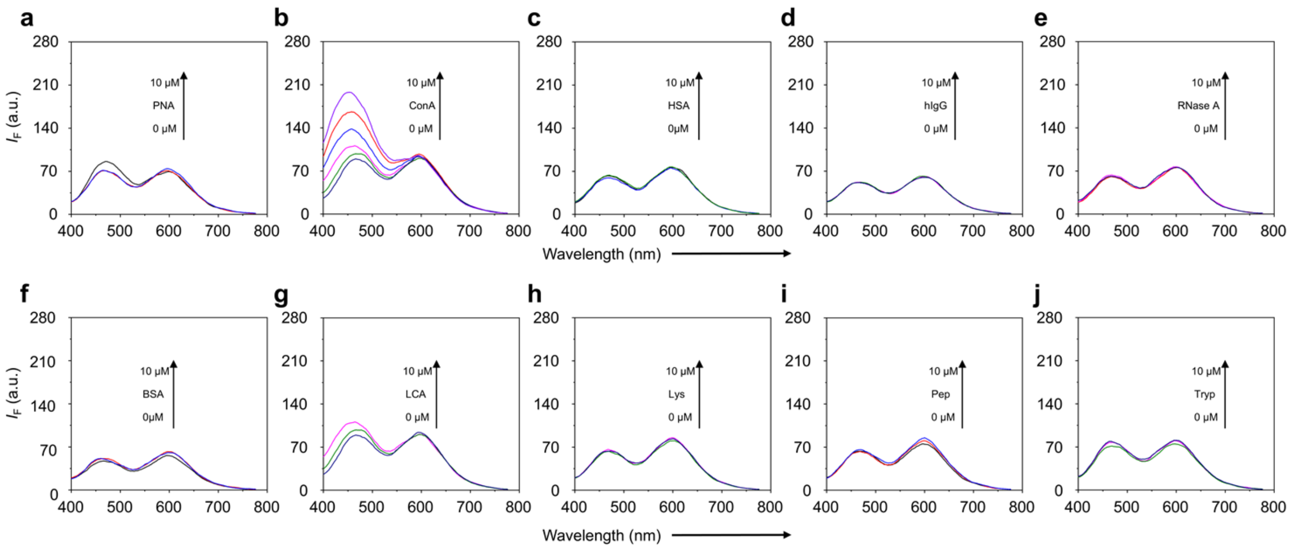

Figure. S4. Concentration-dependent fluorescence changes of **DPAC-Man** in the presence of different proteins (as labeled, 0-10 μM, interval 2 μM) in PBS (0.01 M, pH 7.4) containing 0.02% (v/v) Tween 20; λ_{ex} = 360 nm.

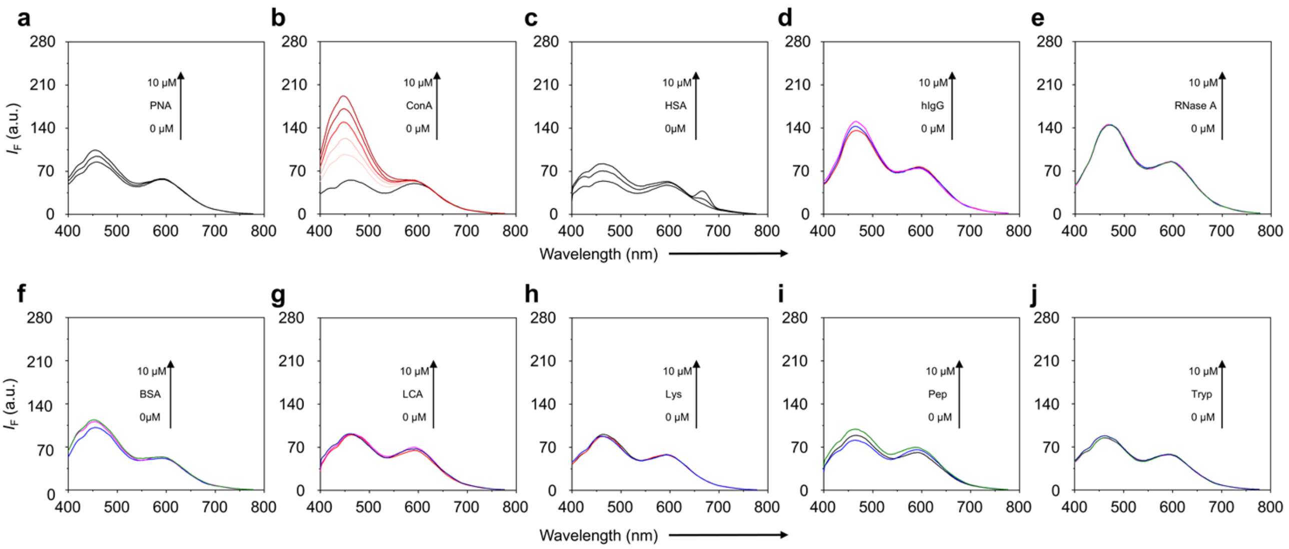

Figure. S5. Concentration-dependent fluorescence response of **DPAC-PEG$_3$-Man** in the presence of different proteins (as labeled, 0-10 μM, interval 2 μM) in PBS (0.01 M, pH 7.4) containing 0.02% (v/v) Tween 20; λ_{ex} = 360 nm.

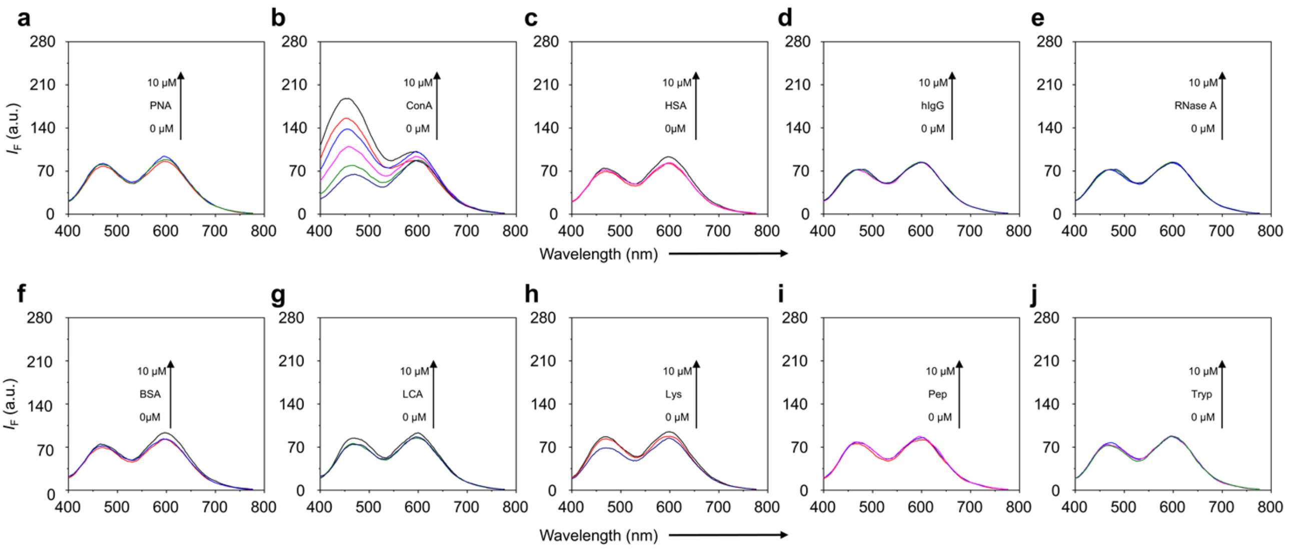

Figure. S6. Concentration-dependent fluorescence response of **DPAC-Glc** in the presence of different proteins (as labeled, 0-10 μM, interval 2 μM) in PBS (0.01 M, pH 7.4) containing 0.02% (v/v) Tween 20; λ_{ex} = 360 nm.

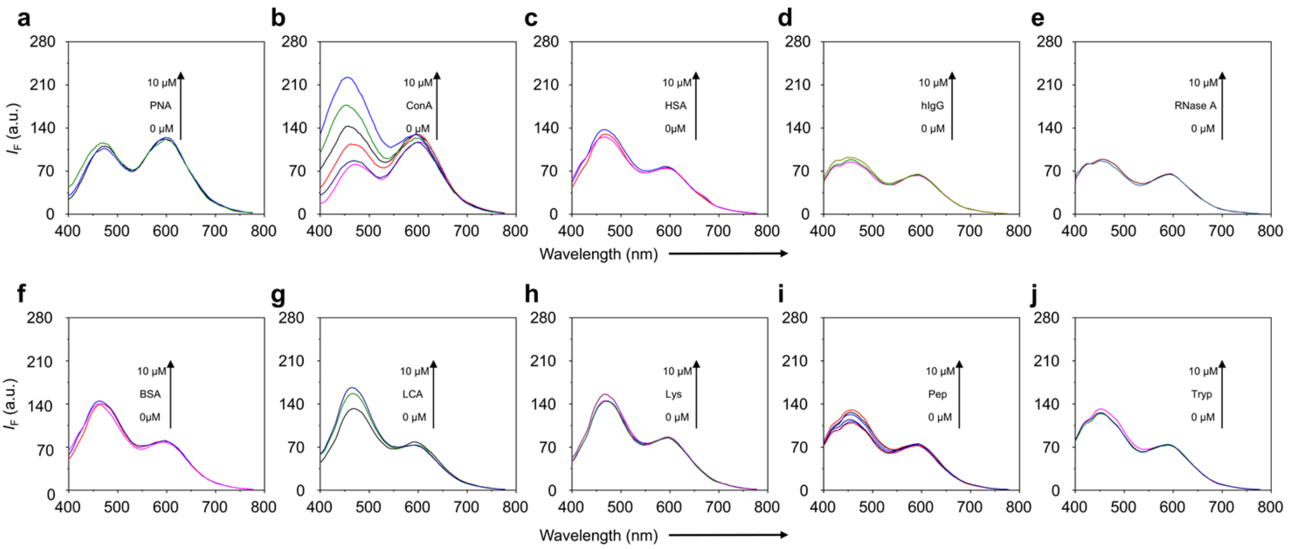

Figure. S7. Concentration-dependent fluorescence response of **DPAC-PEG$_3$-Glc** in the presence of different proteins (as labeled, 0-10 μM, interval 2 μM) in PBS (0.01 M, pH 7.4) containing 0.02% (v/v) Tween 20; λ_{ex} = 360 nm.

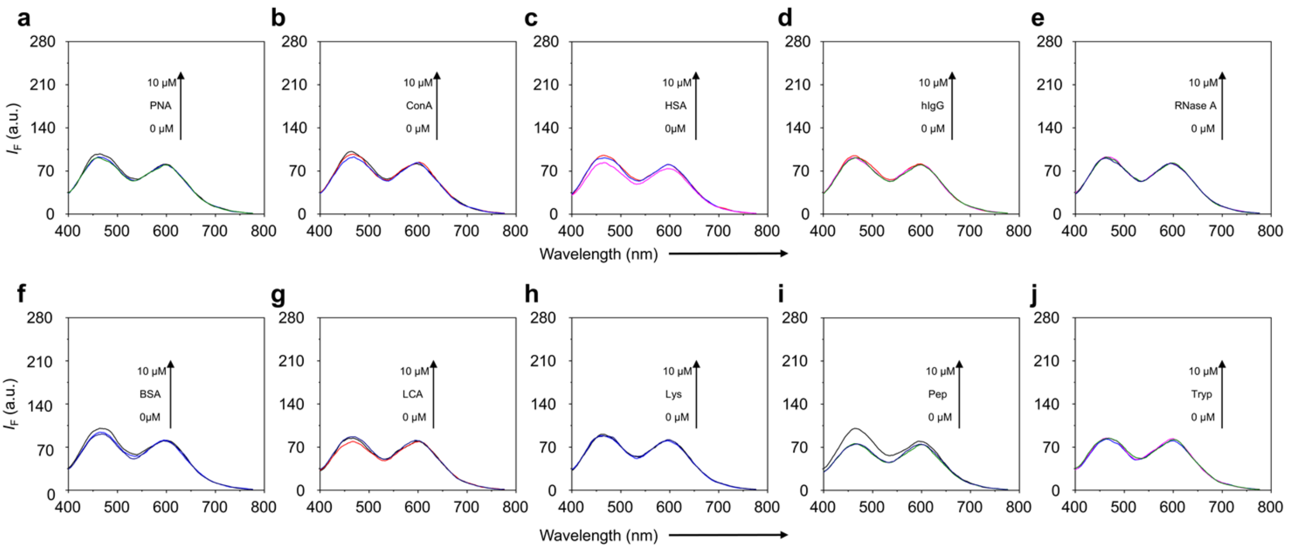

Figure. S8. Concentration-dependent fluorescence response of **DPAC-Fuc** in the presence of different proteins (as labeled, 0-10 μM, interval 2 μM) in PBS (0.01 M, pH 7.4) containing 0.02% (v/v) Tween 20; λ_{ex} = 360 nm.

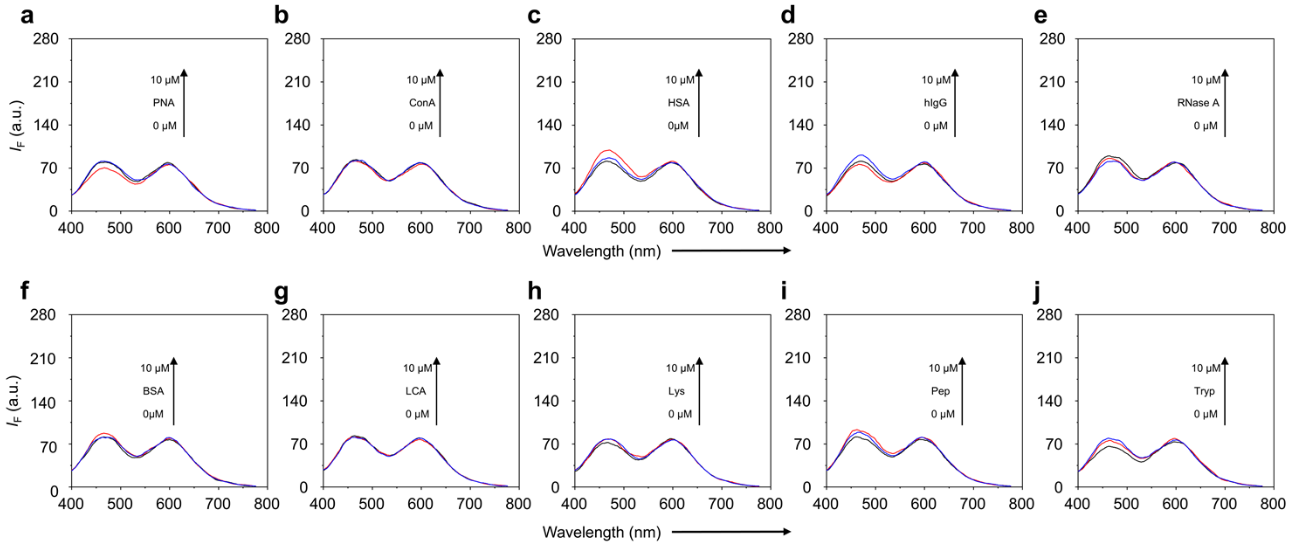

Figure. S9. Concentration-dependent fluorescence response of **DPAC-PEG$_3$-Fuc** in the presence of different proteins (as labeled, 0-10 μM, interval 2 μM) in PBS (0.01 M, pH 7.4) containing 0.02% (v/v) Tween 20; λ_{ex} = 360 nm.

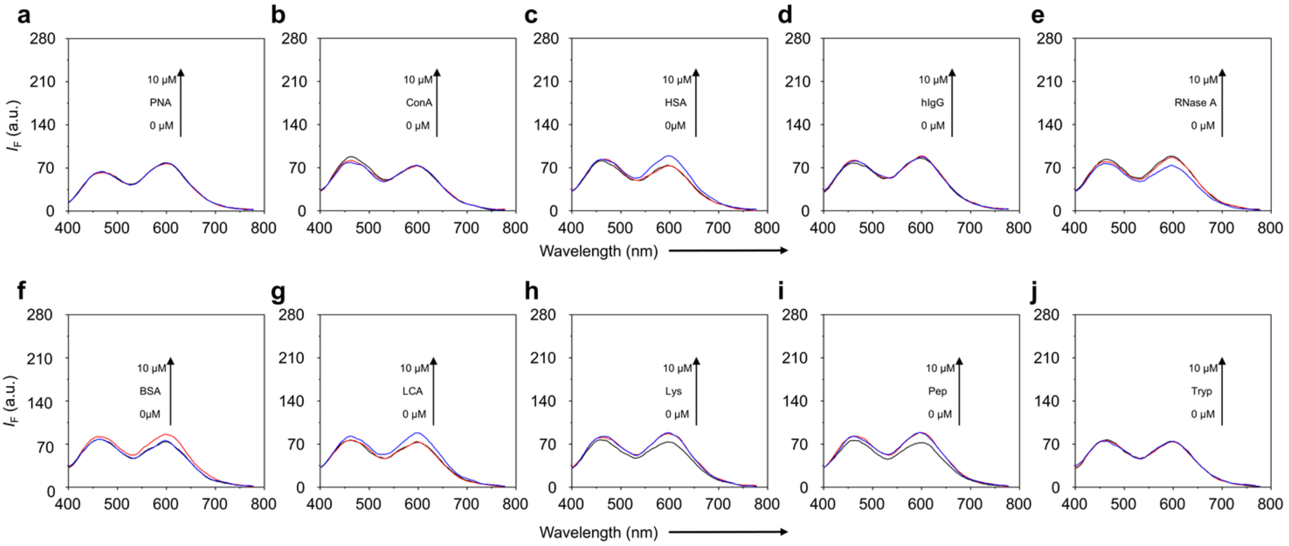

Figure. S10. Concentration-dependent fluorescence response of **DPAC-OH** in the presence of different proteins (as labeled, 0-10 μM, interval 2 μM) in PBS (0.01 M, pH 7.4) containing 0.02% (v/v) Tween 20; λ_{ex} = 360 nm.

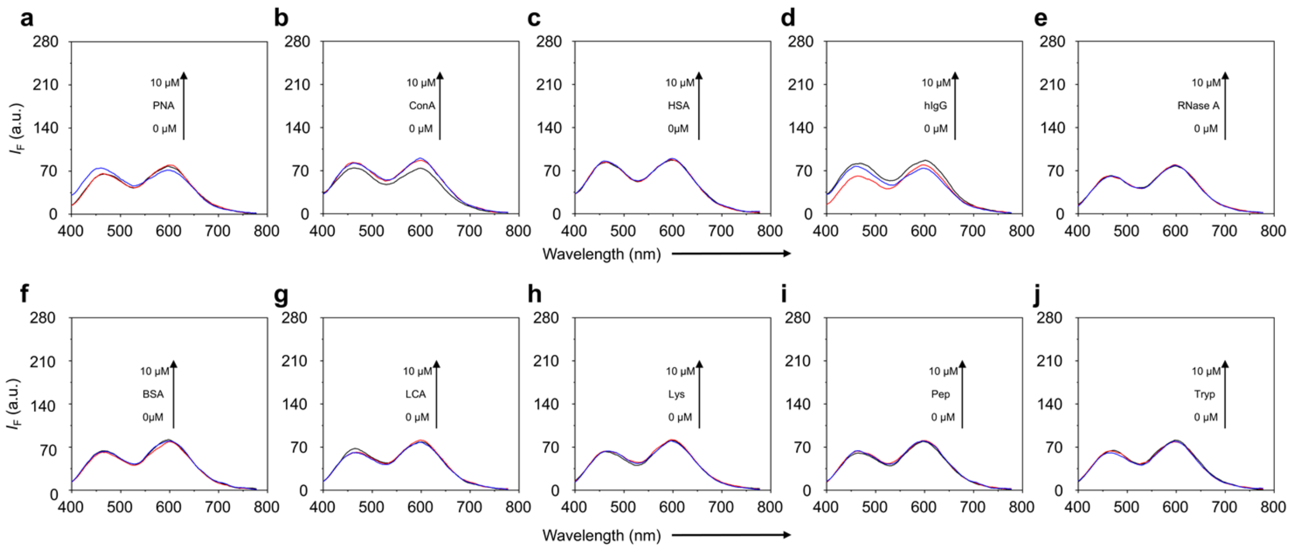

Figure. S11 Concentration-dependent fluorescence response of **DPAC-PEG$_3$-OH** in the presence of different proteins (as labeled, 0-10 μM, interval 2 μM) in PBS (0.01 M, pH 7.4) containing 0.02% (v/v) Tween 20; λ_{ex} = 360 nm.

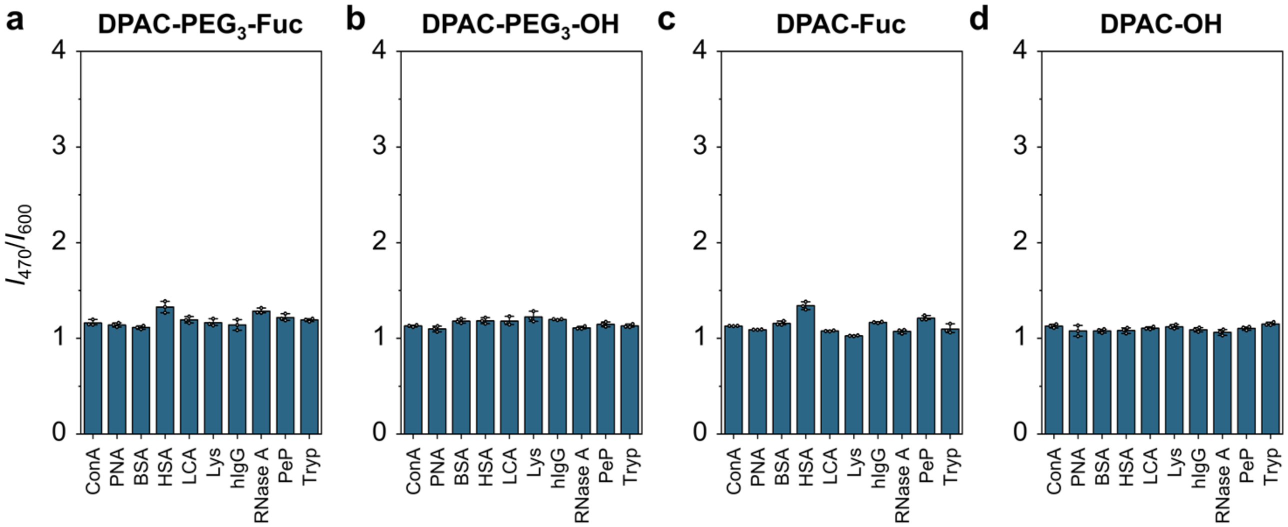

Figure. S12. Fluorescence ratiometric changes (I_{470}/I_{600} emission ratio) of (a) **DPAC-PEG$_3$-Fuc**, (b) **DPAC-PEG$_3$-OH**, (c) **DPAC-Fuc**, and (d) **DPAC-OH** (10 μM) after incubation with different proteins in PBS (0.01 M, pH 7.4) containing 0.02% (v/v) Tween 20. Data were collected at λ_{ex}= 360 nm; error bars represent the standard deviation of three independent measurements.

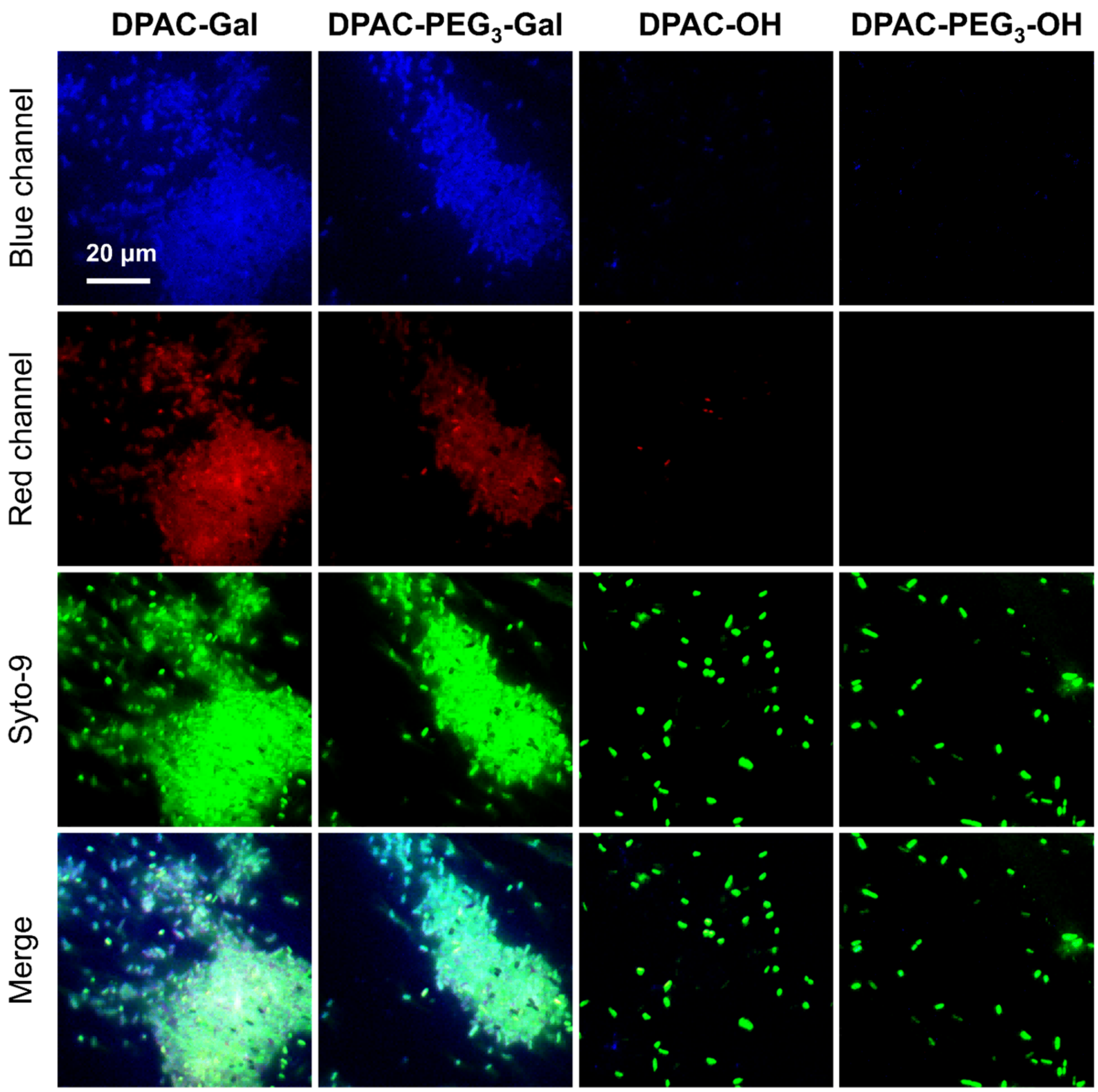

Figure S13. Confocal laser-scanning fluorescence microscopy images of *P. aeruginosa* (ATCC 27853) (10^8 CFU mL^{-1}) after incubation with **DPAC-Gal**, **DPAC-PEG$_3$-Gal**, **DPAC-OH**, or **DPAC-PEG$_3$-OH** (20 μM) for 60 min, and co-staining with Syto-9 (2.5 μM) for another 15 min. The blue channel (420-500 nm) and red channel (570-650 nm) were excited by a 405 nm laser diode. The Syto-9 channel (green) was used to indicate the location of the bacteria.

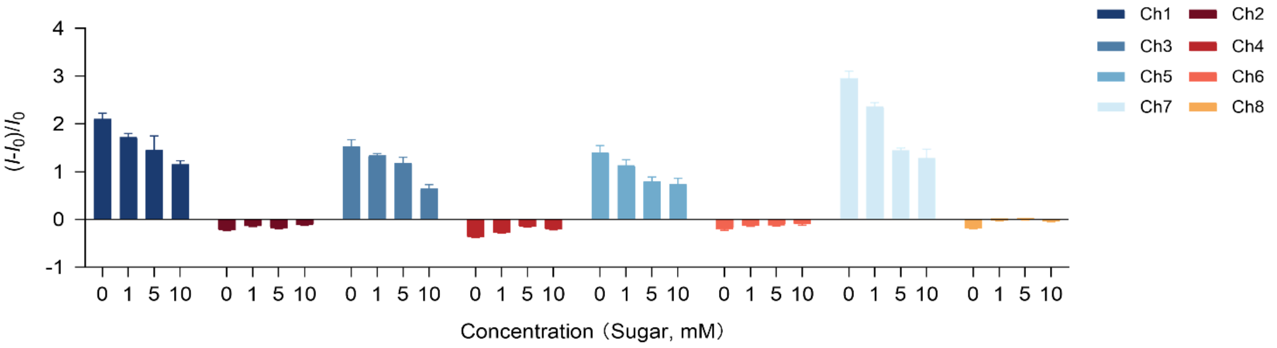

Figure S14. Competitive binding assay. Fluorescence responses $(I\text{-}I_0)/I_0$ of **DPAC-Gal** (Ch1/2), **DPAC-Fuc** (Ch3/4), **DPAC-Man** (Ch5/6), and **DPAC-Glc** (Ch7/8) (10 μM) incubated with P. aeruginosa (ATCC 27853, OD_{600} = 1.0) for 60 min, following the pre-incubation of the bacteria with the corresponding free monosaccharides at varied concentrations (0, 1, 5, and 10 mM) for 1 h. Odd and even channels correspond to the blue-emission (470 nm) and red-emission (600 nm), respectively; λ_{ex} = 360 nm. Error bars represent the standard deviation of three independent measurements.

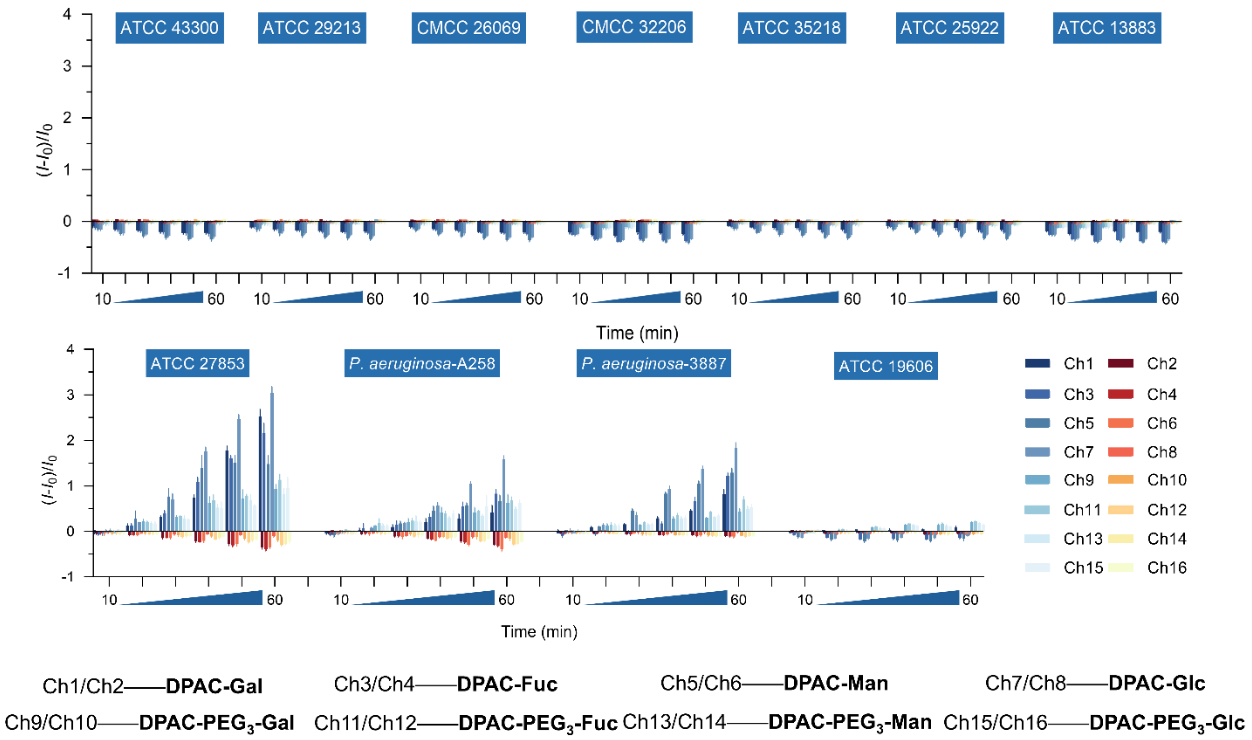

Figure. S15. Fluorescence response [(I–I_0)/I_0] of glycoprobe-based sensor array to different indicated bacteria. Odd (Ch1, 3, 5, 7, 9, 11, 13, 15) and even channels (Ch2, 4, 6, 8, 10, 12, 14, 16) correspond to the blue-emission (470 nm) and red-emission (600 nm) of the probes, respectively, with excitation of 360 nm.

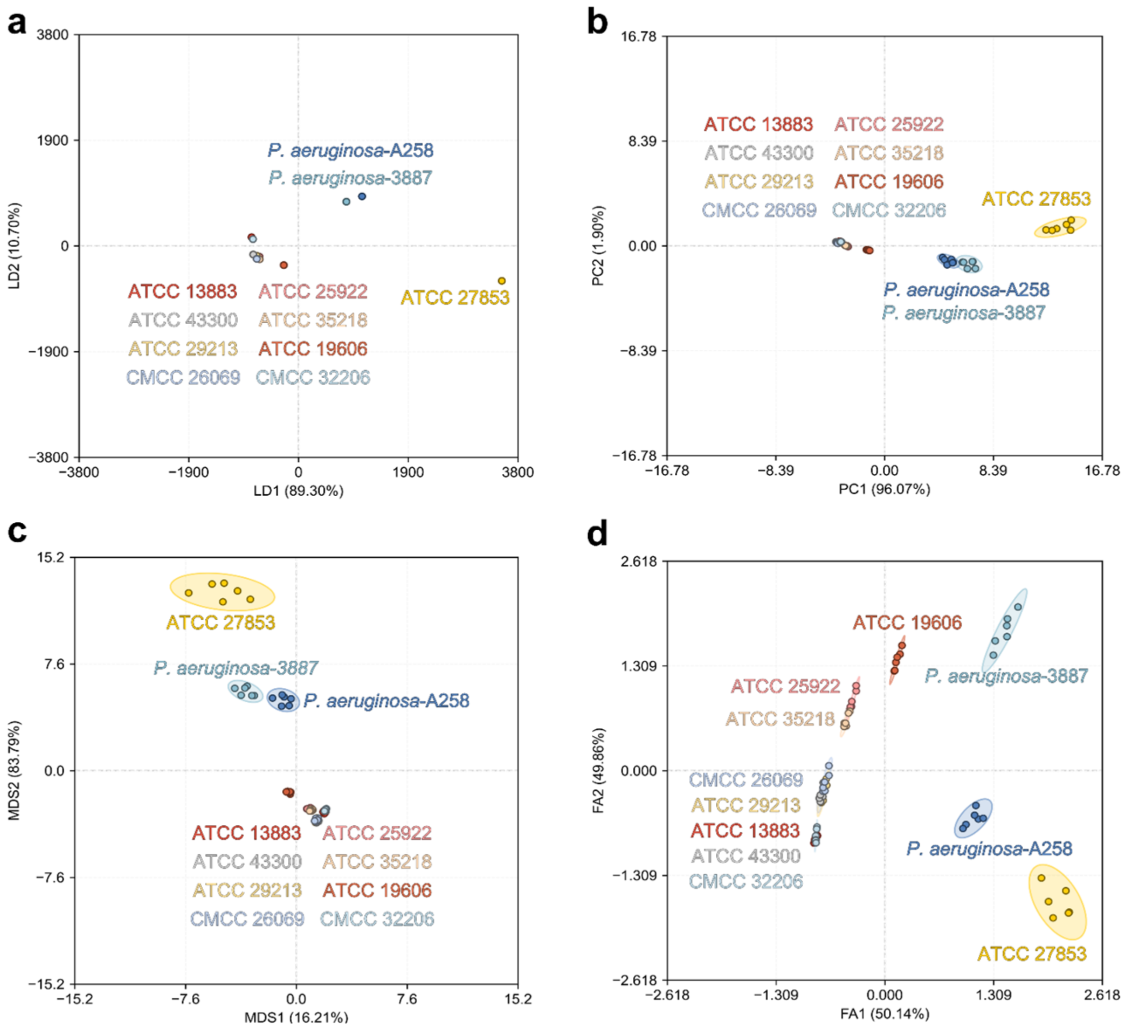

Figure S16. (a) LDA, (b) PCA, (c) MDS and (d) FA score plots generated from the ratiometric changes in fluorescence intensity (I_{470}/I_{600}) shown in Figure 3 for bacterial classification.

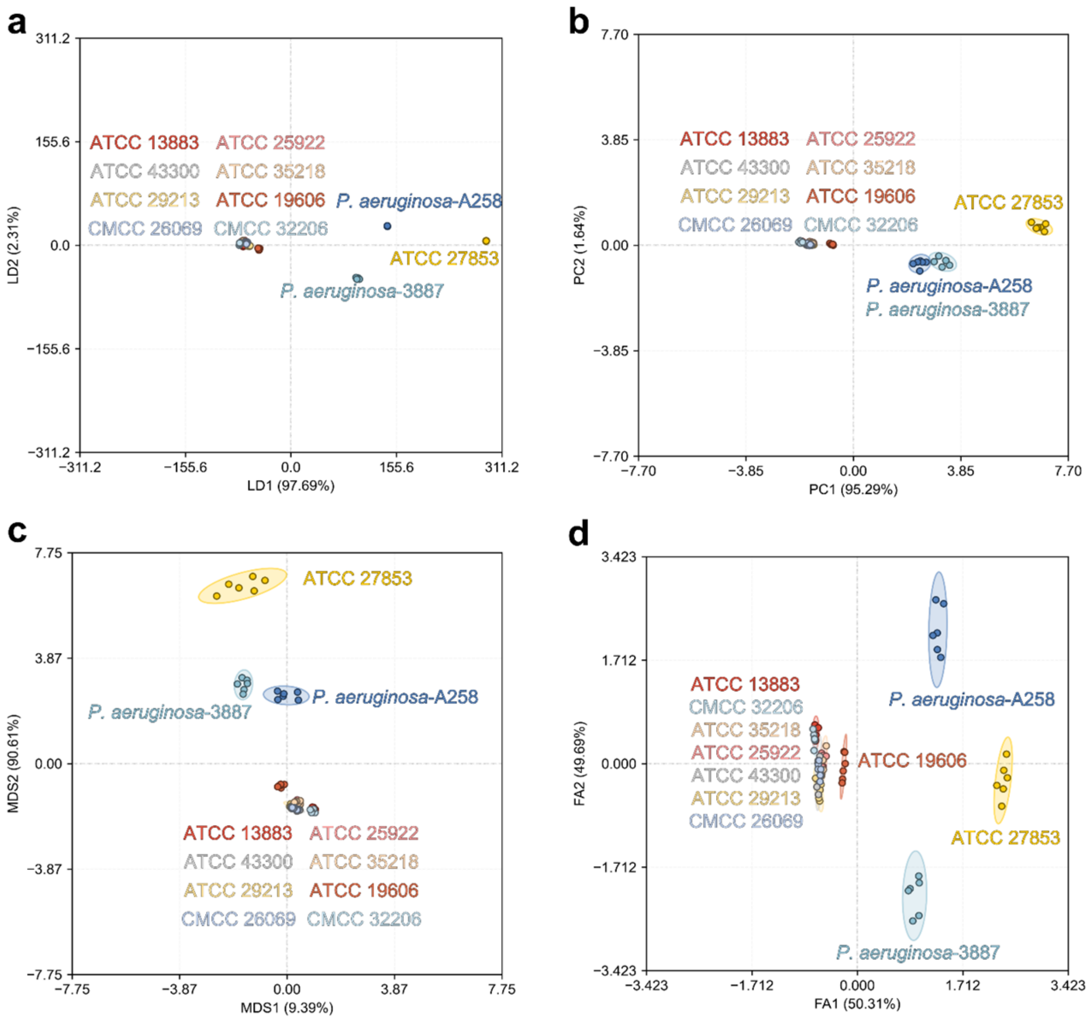

Figure S17. (a) LDA, (b) PCA, (c) MDS and (d) FA score plots obtained from the independent fluorescence intensities I_{470} and I_{600} shown in Figure 3 for bacterial classification.

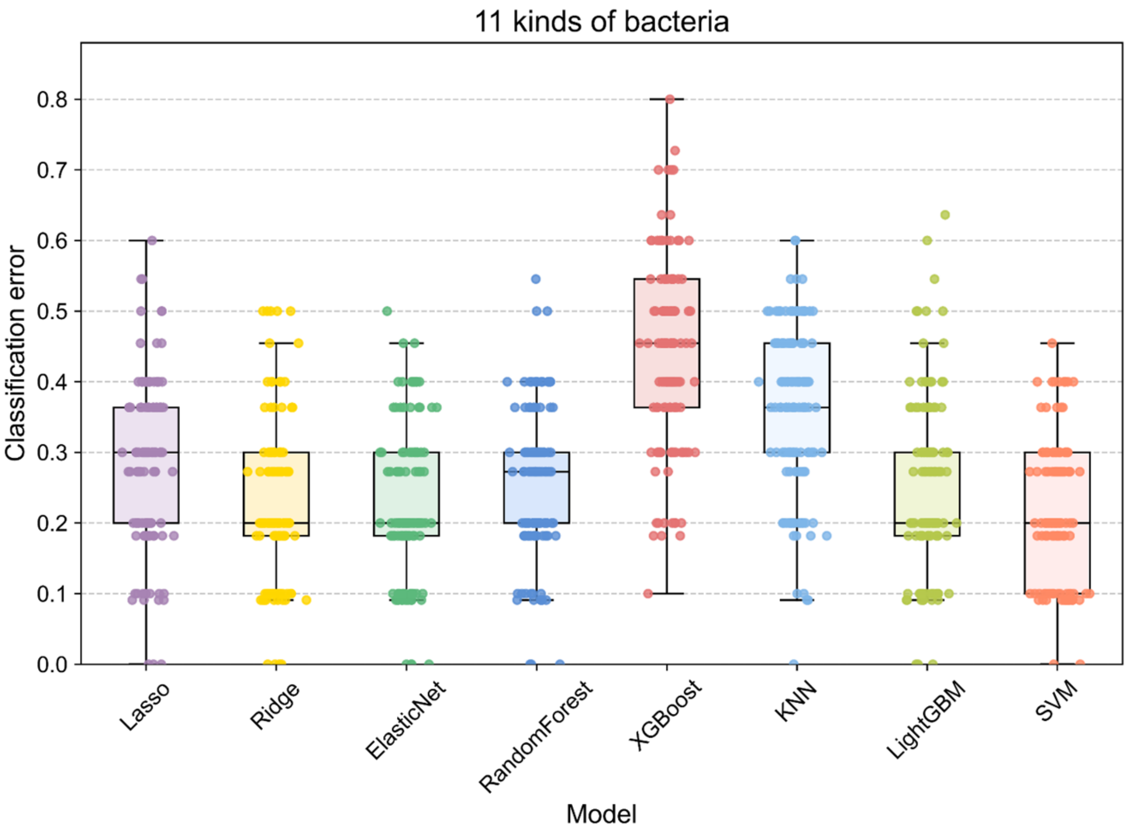

Figure S18. Boxplot of bacterial classification error rates (test set) across eight algorithms using the fluorescence signals from Figure S13.

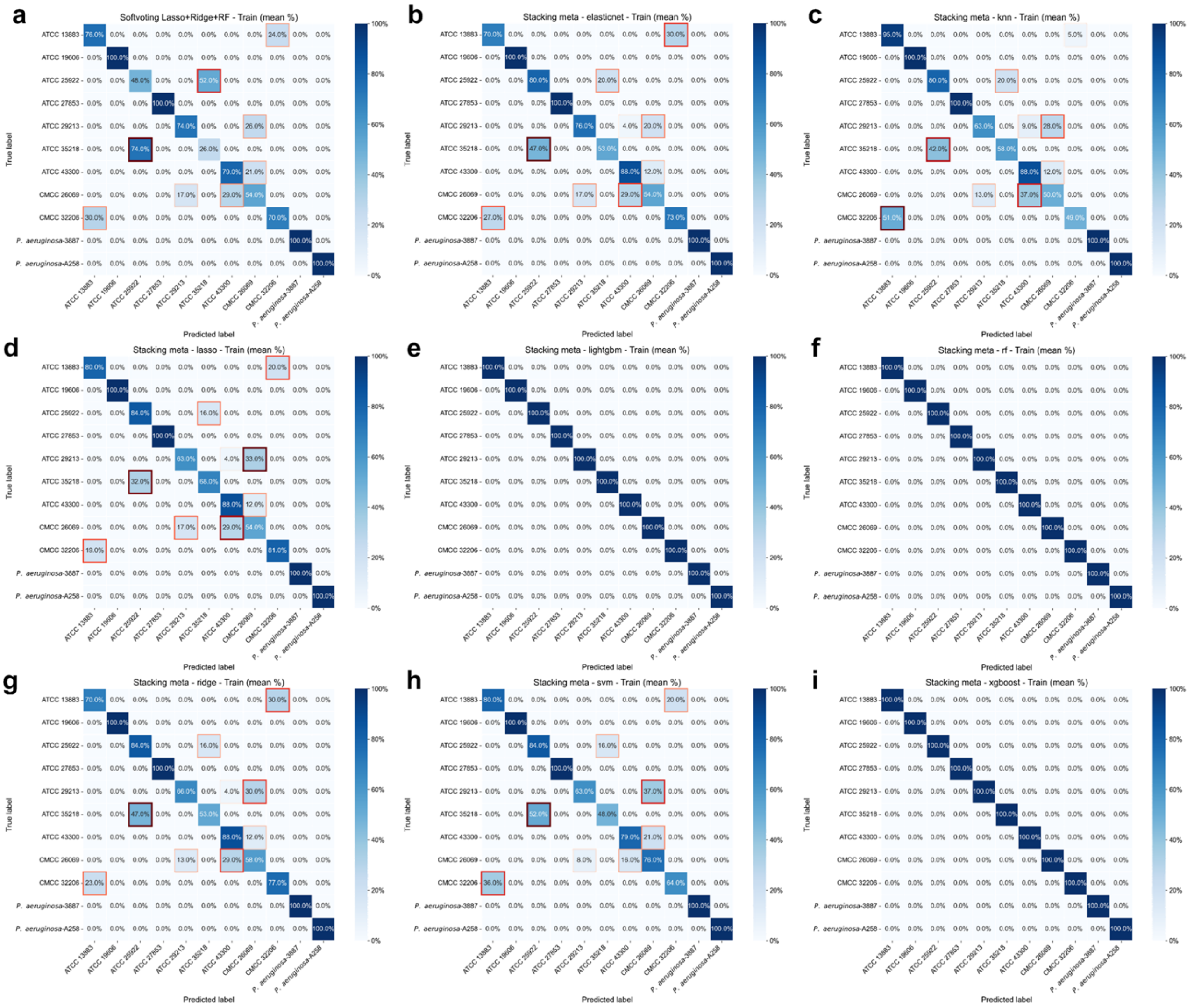

Figure S19. Training set confusion matrices for the nine ML algorithms applied to dual-channel fluorescence intensities from the glycoprobe-based sensor array. (a) Three-tier data-fusion with soft-voting ensemble. (b–i) Stacking ensemble variants: (b) ElasticNet, (c) K-nearest neighbors (KNN), (d) Lasso, (e) LightGBM, (f) Random Forest (RF), (g) Ridge, (h) support vector machine (SVM), and (i) XGBoost.

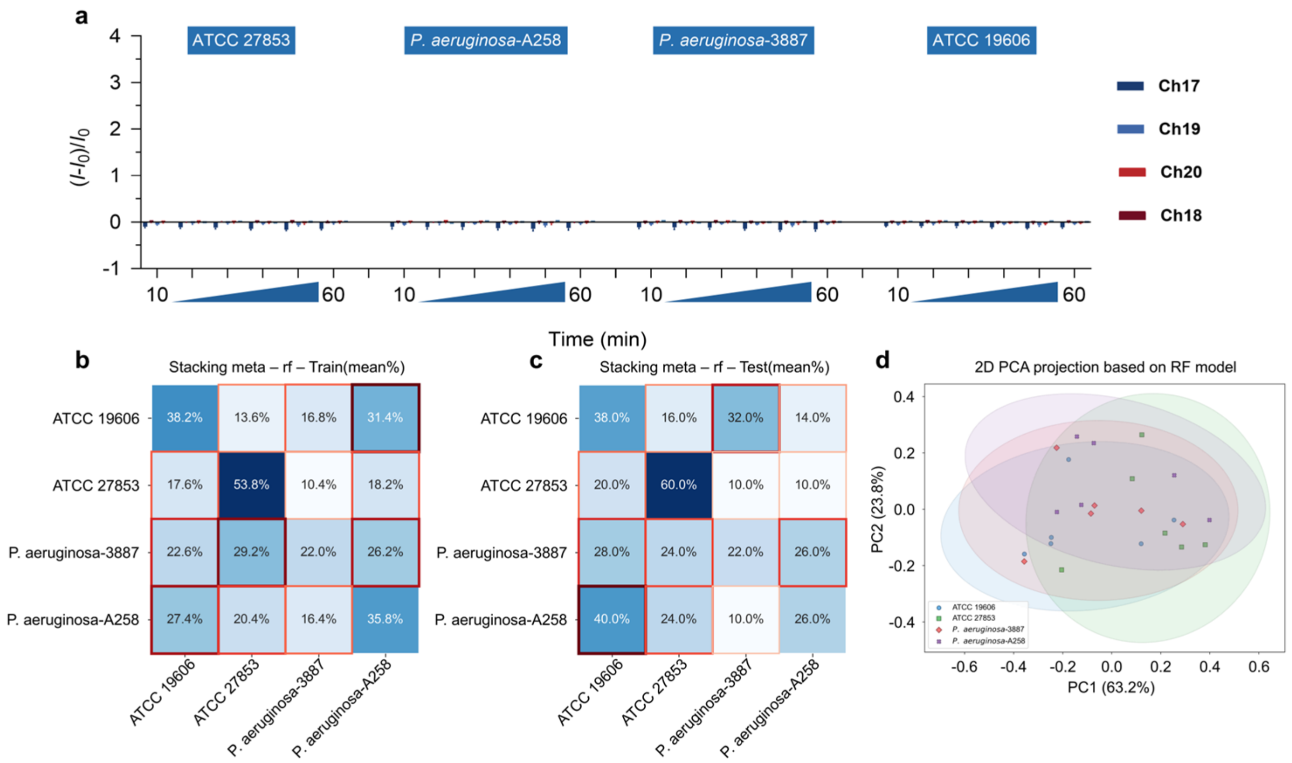

Figure S20. Sensing and classification performance of control probes. (a) Time-dependent fluorescence responses [$(I\text{-}I_0)/I_0$] of **DPAC-OH** (Ch17/18) and **DPAC-PEG$_3$-OH** (Ch19/20) to the indicated lectin-expressing bacterial strains. Confusion matrices for the (b) training set and (c) test set using the stacking ensemble model based solely on the data from the control probes. (d) 2D PCA projection based on the RF model.

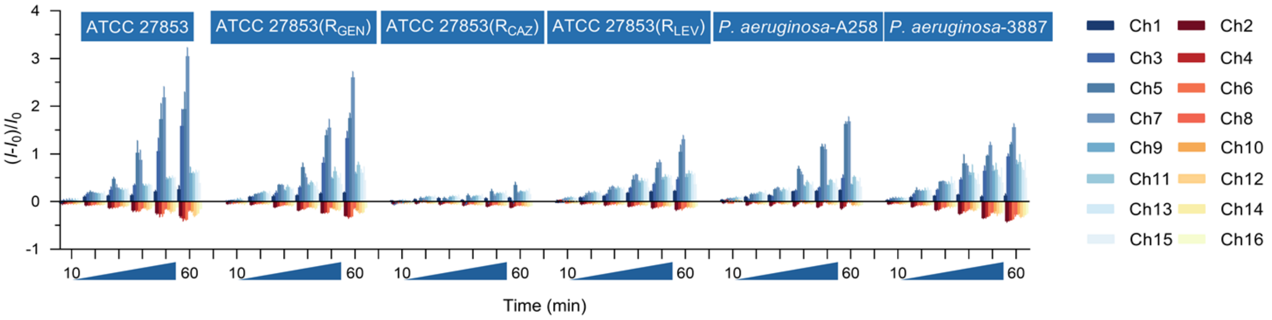

Figure S21. Fluorescence response [$(I–I_0)/I_0$] of glycoprobe-based sensor array to antibiotic-sensitive *vs.* resistant *P. aeruginosa*. Odd (Ch1, 3, 5, 7, 9, 11, 13, 15) and even channels (Ch2, 4, 6, 8, 10, 12, 14, 16) correspond to the blue-emission (470 nm) and red-emission (600 nm) of the probes, respectively, with excitation of 360 nm.

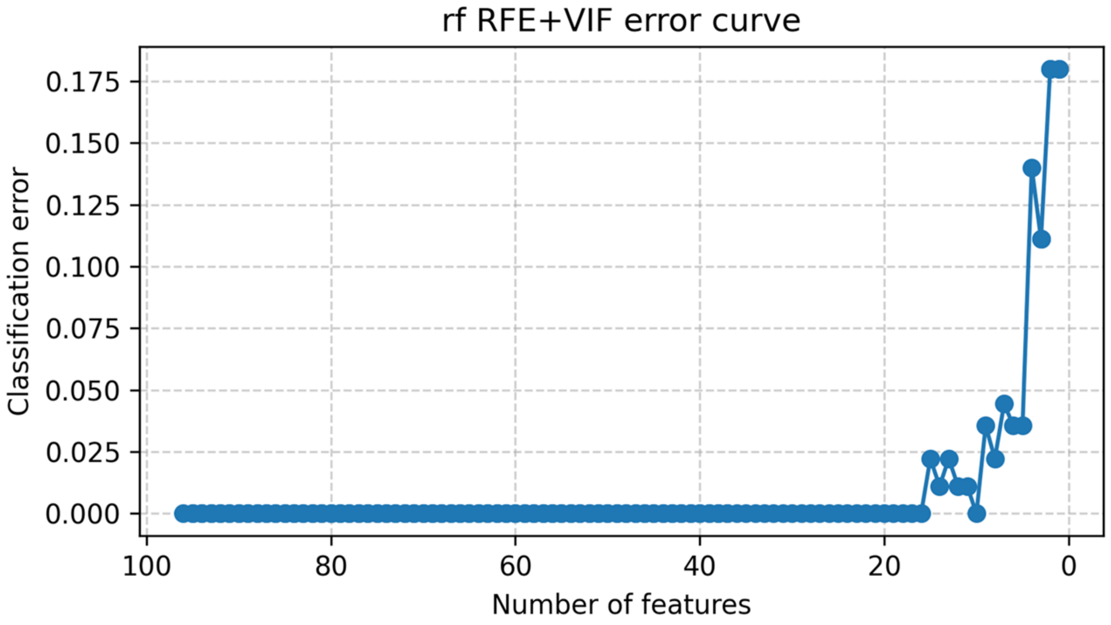

Figure S22. Classification error rate versus number of retained variables during feature extraction.

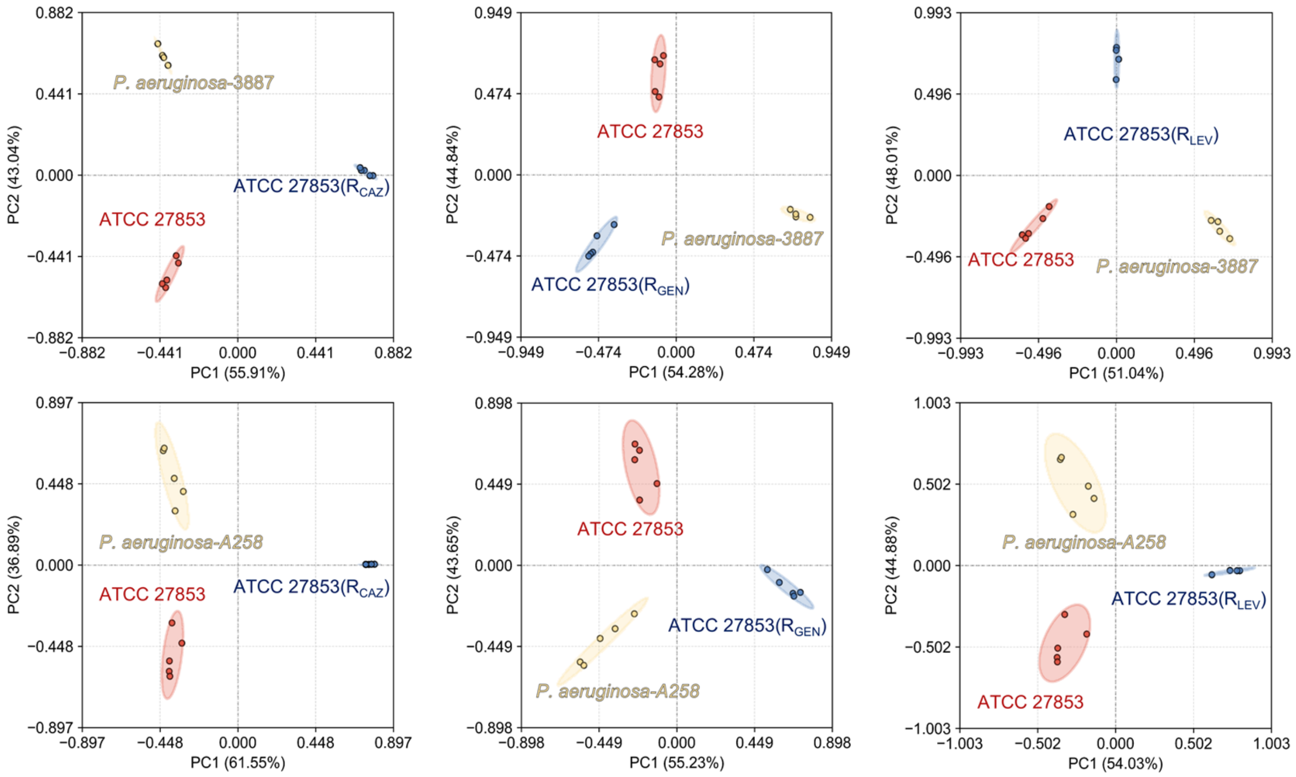

Figure S23. 2D Random-Forest projection of different *P. aeruginosa* strains obtained with the top 16 selected variables.

S3. Original spectra of new compounds

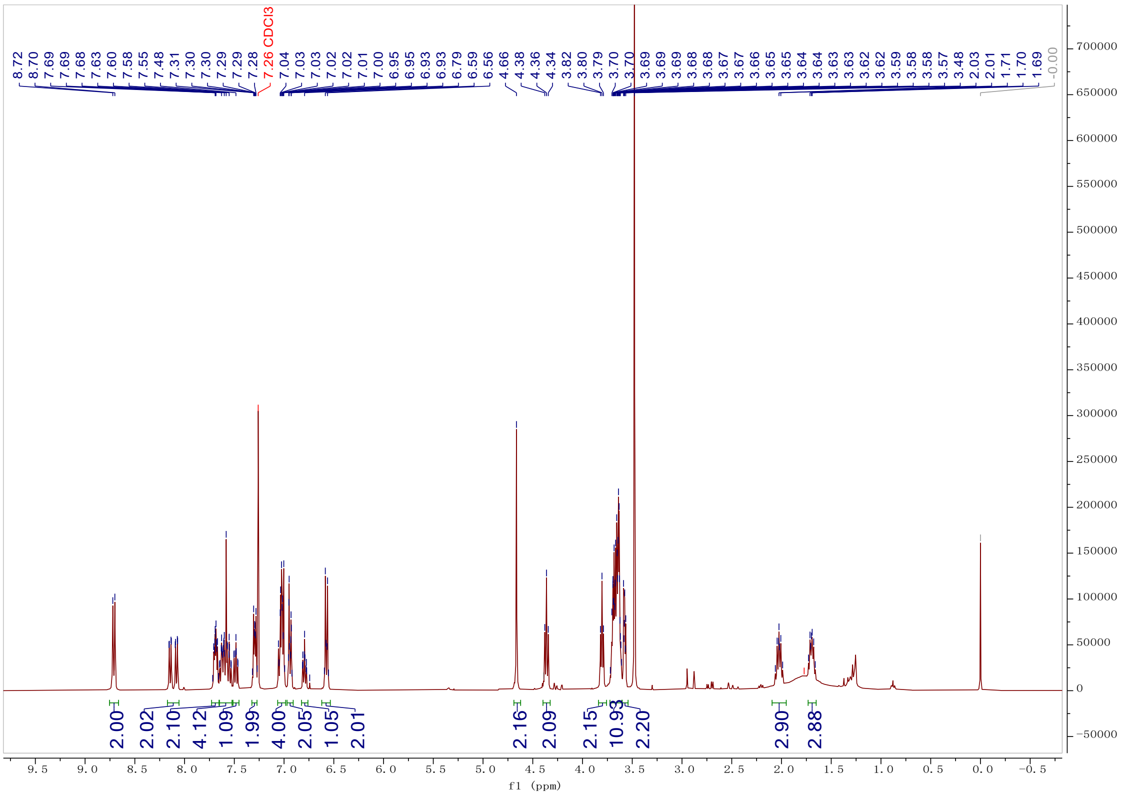

^{1}H NMR of **DPAC-PEG$_3$-OH**

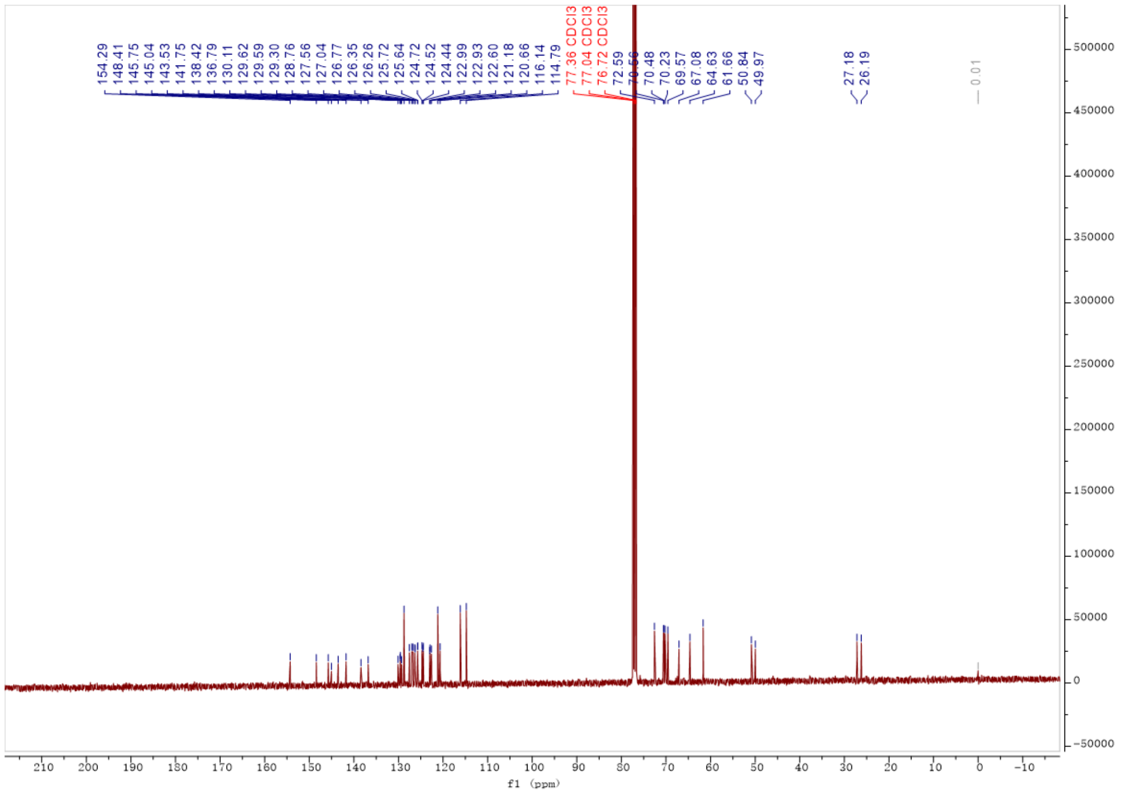

^{13}C NMR of **DPAC-PEG$_3$-OH**

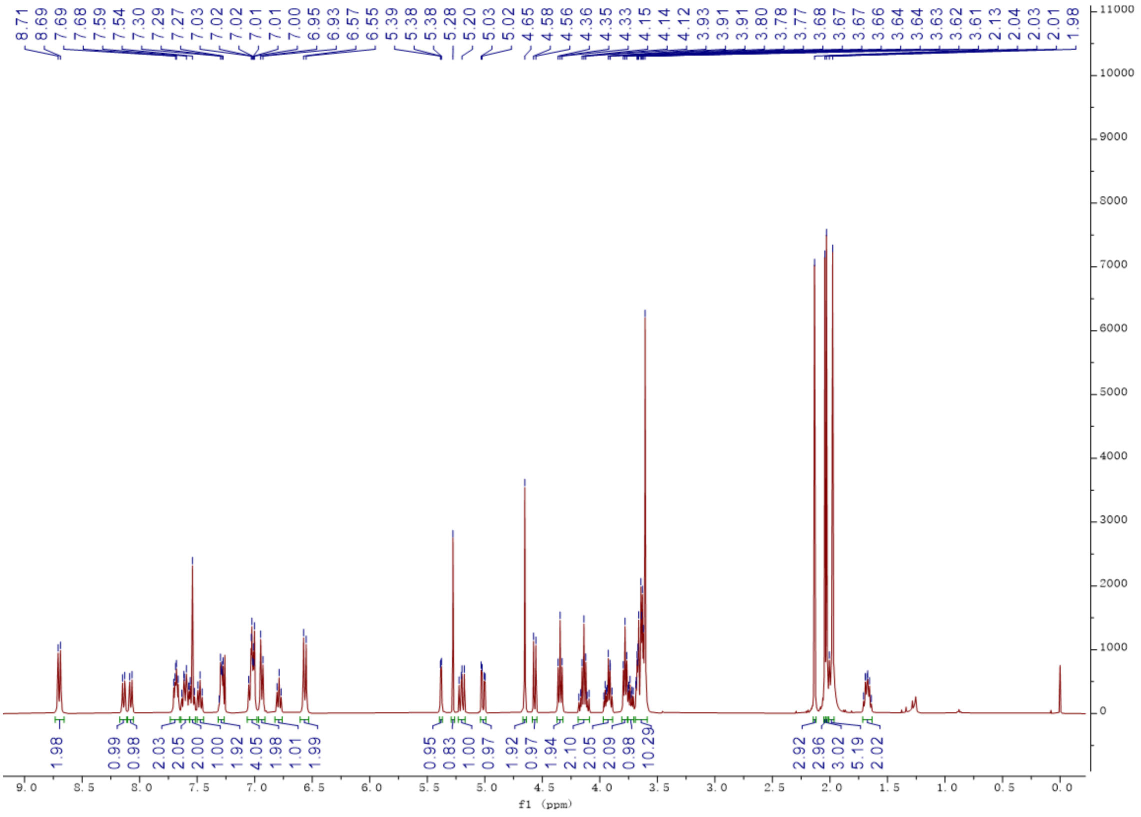

^{1}H NMR of **G9**

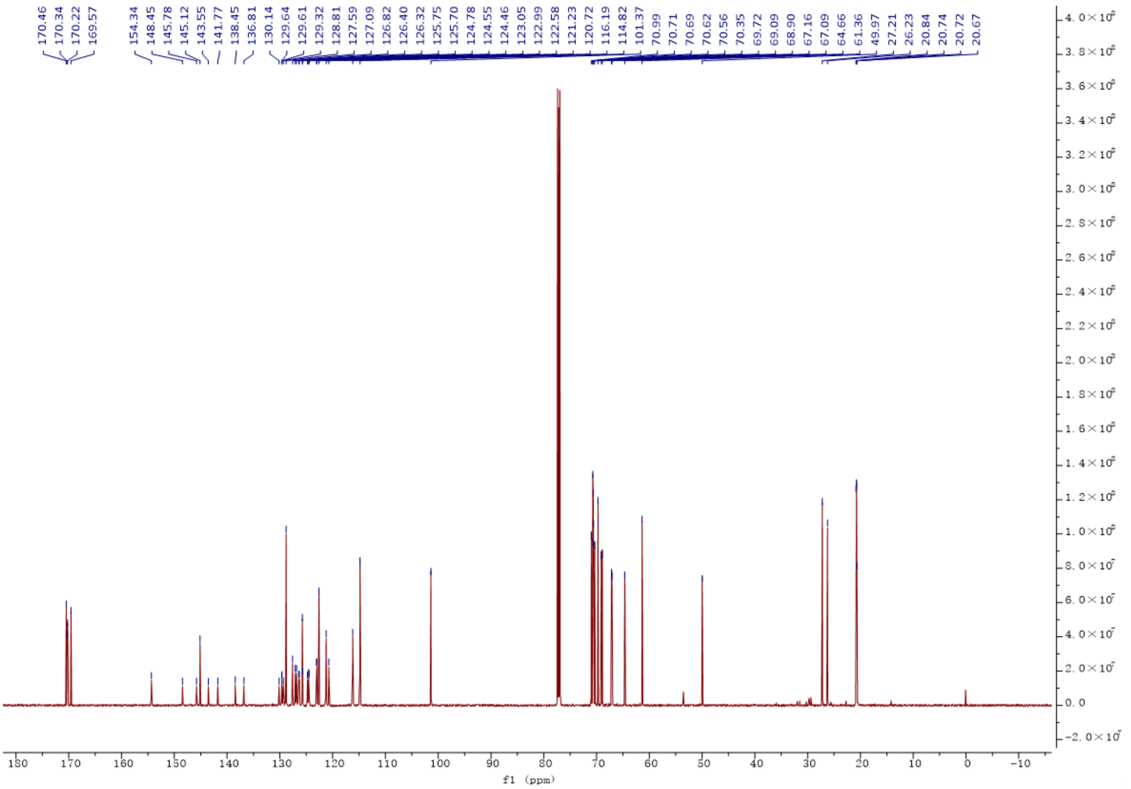

^{13}C NMR of **G9.**

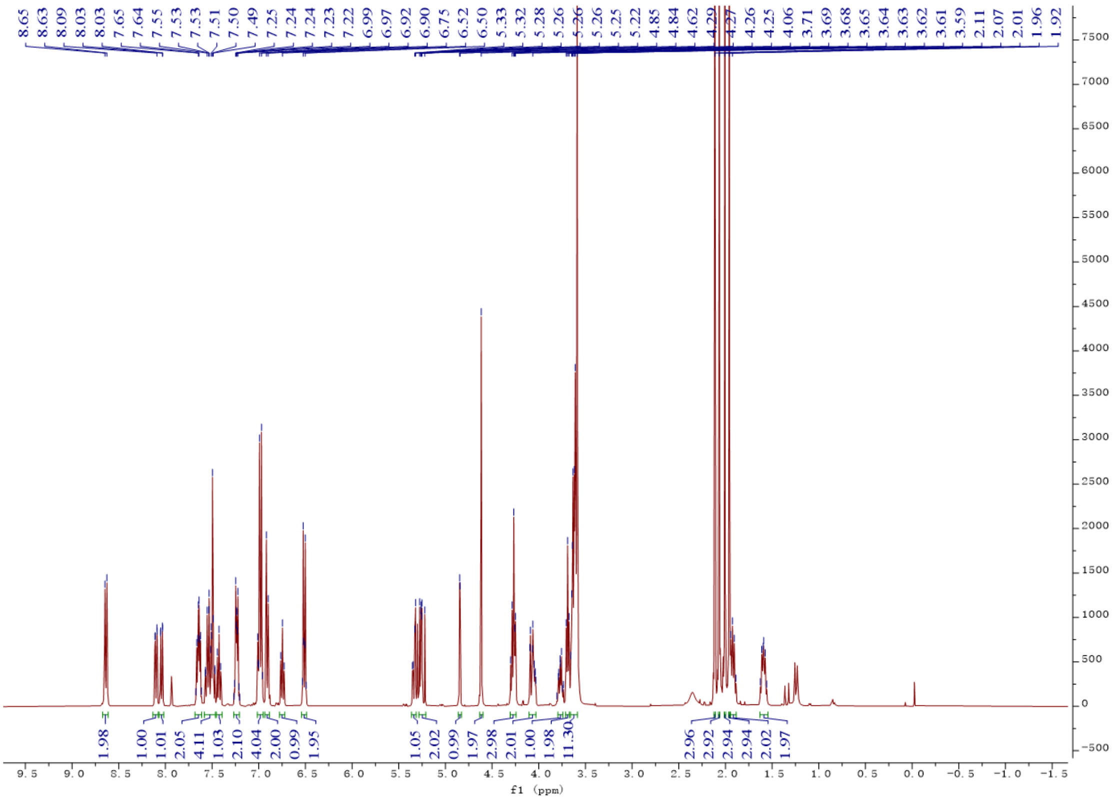

^{1}H NMR of **G10.**

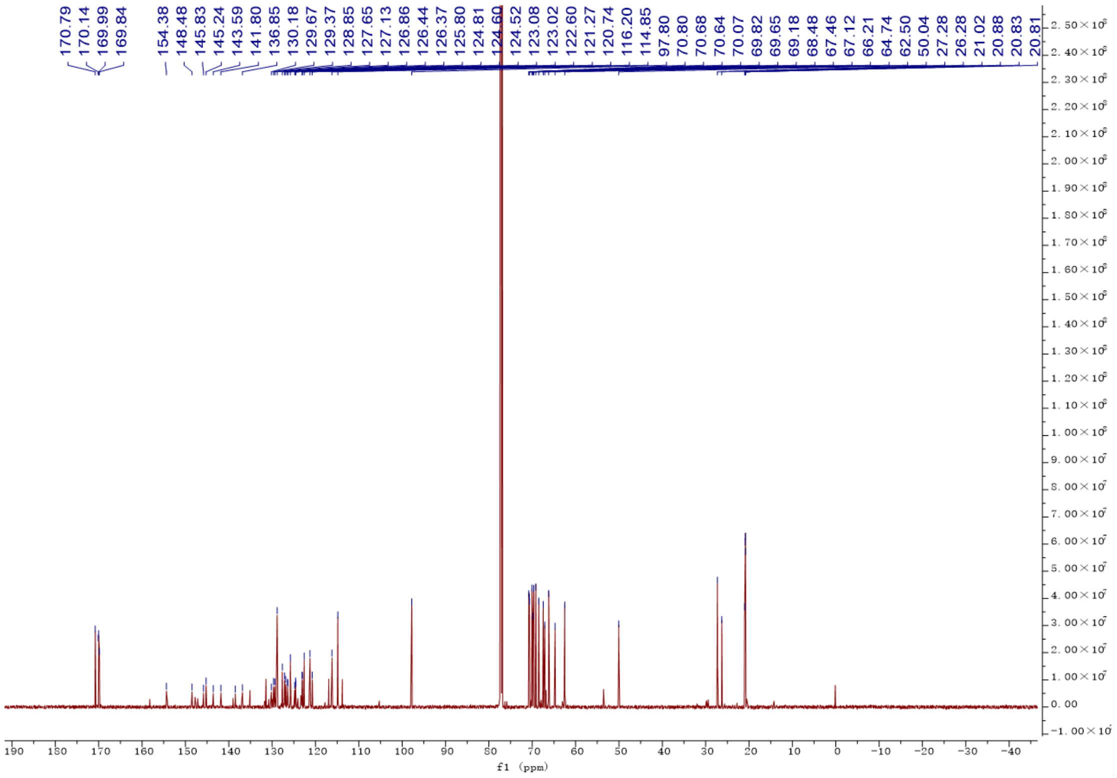

^{13}C NMR of **G10.**

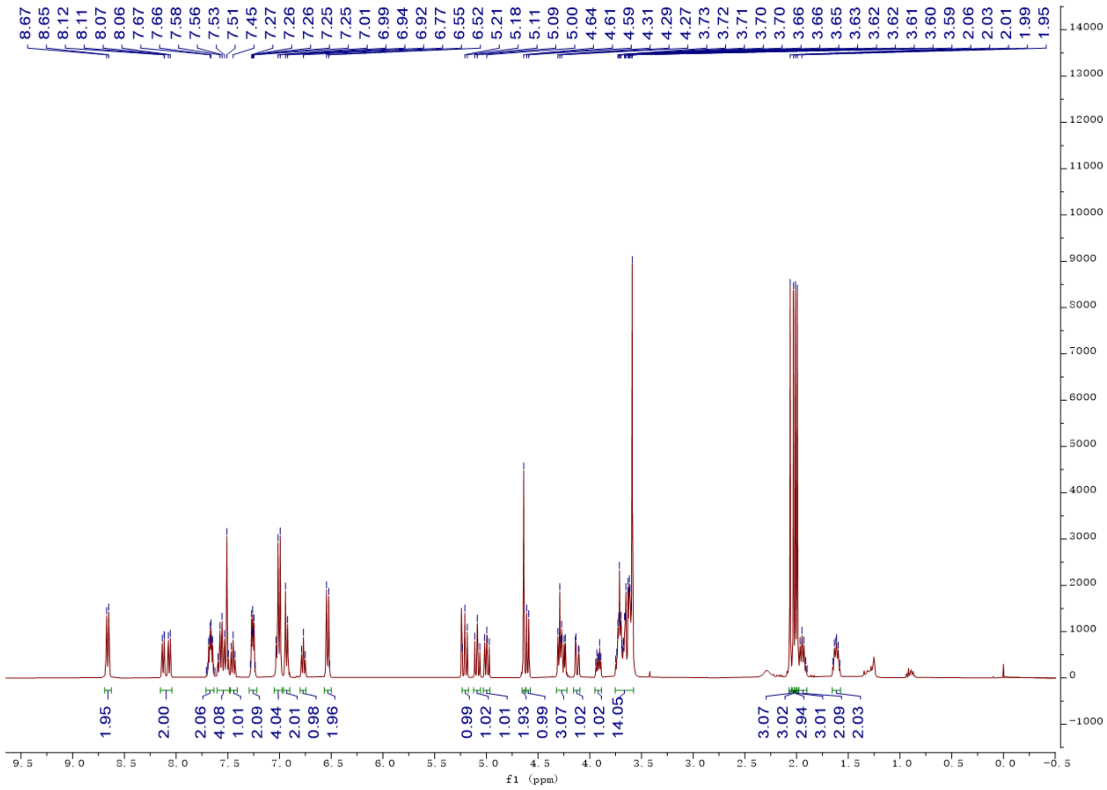

^{1}H NMR of **G11.**

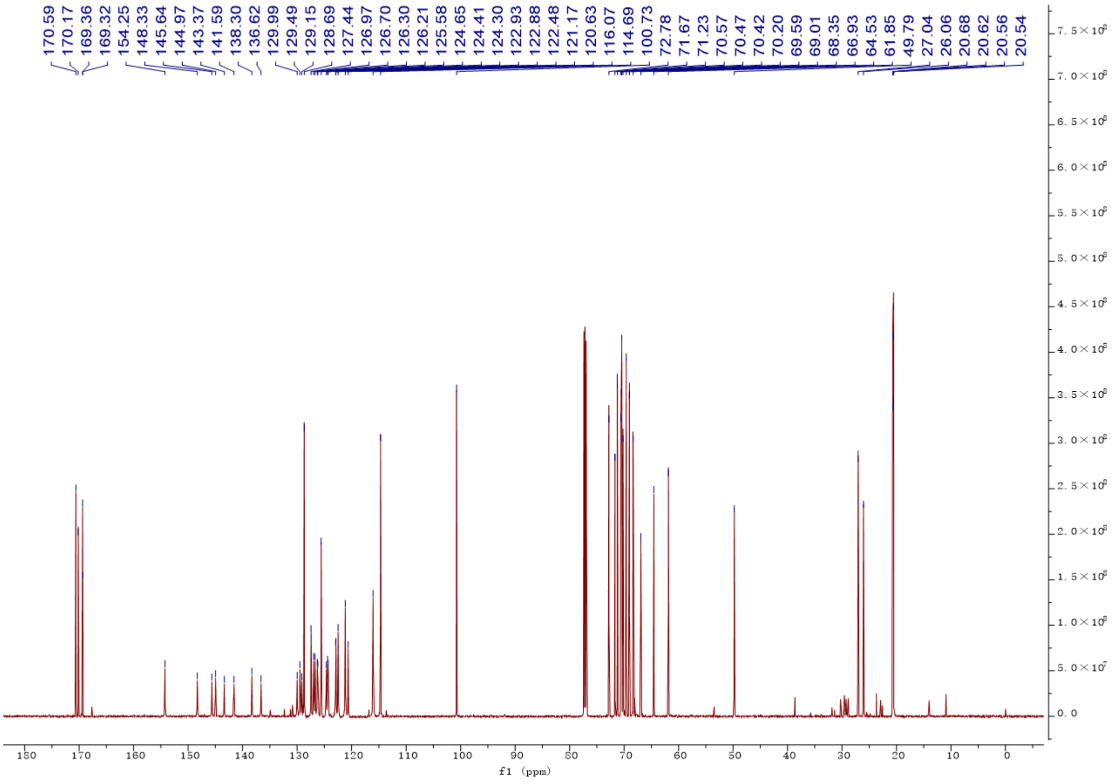

^{13}C NMR of **G11.**

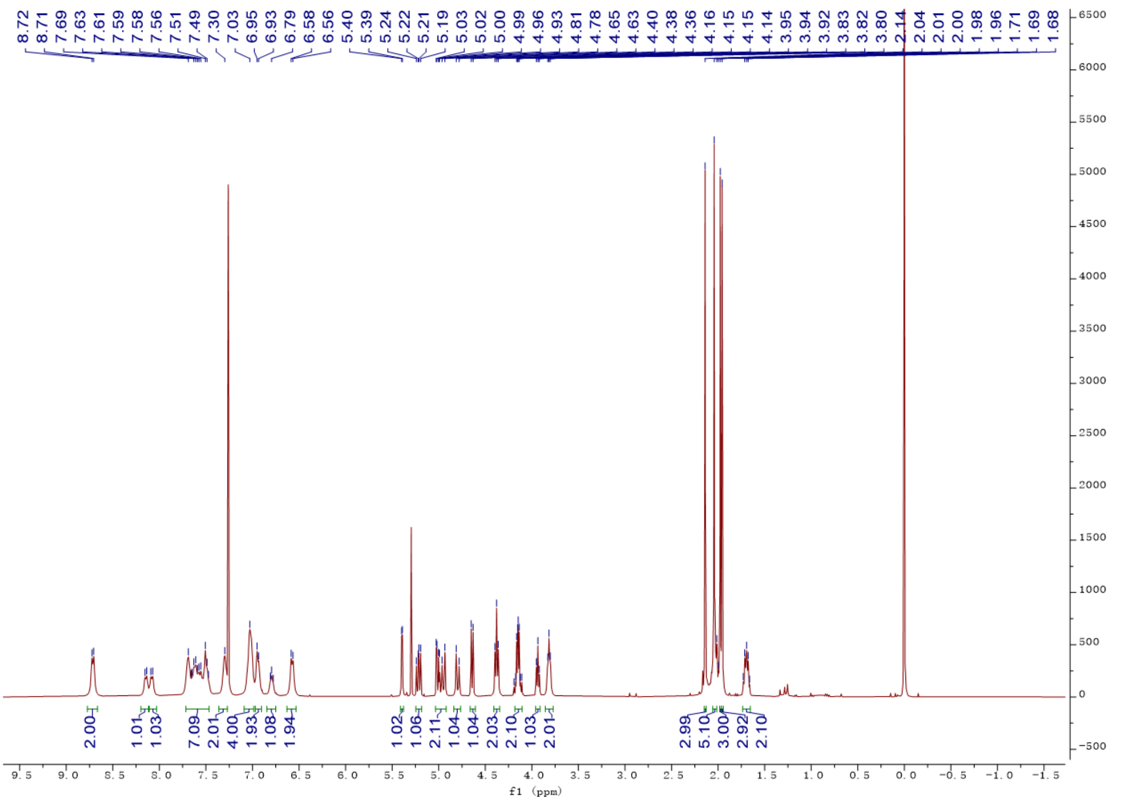

^{1}H NMR of **G13.**

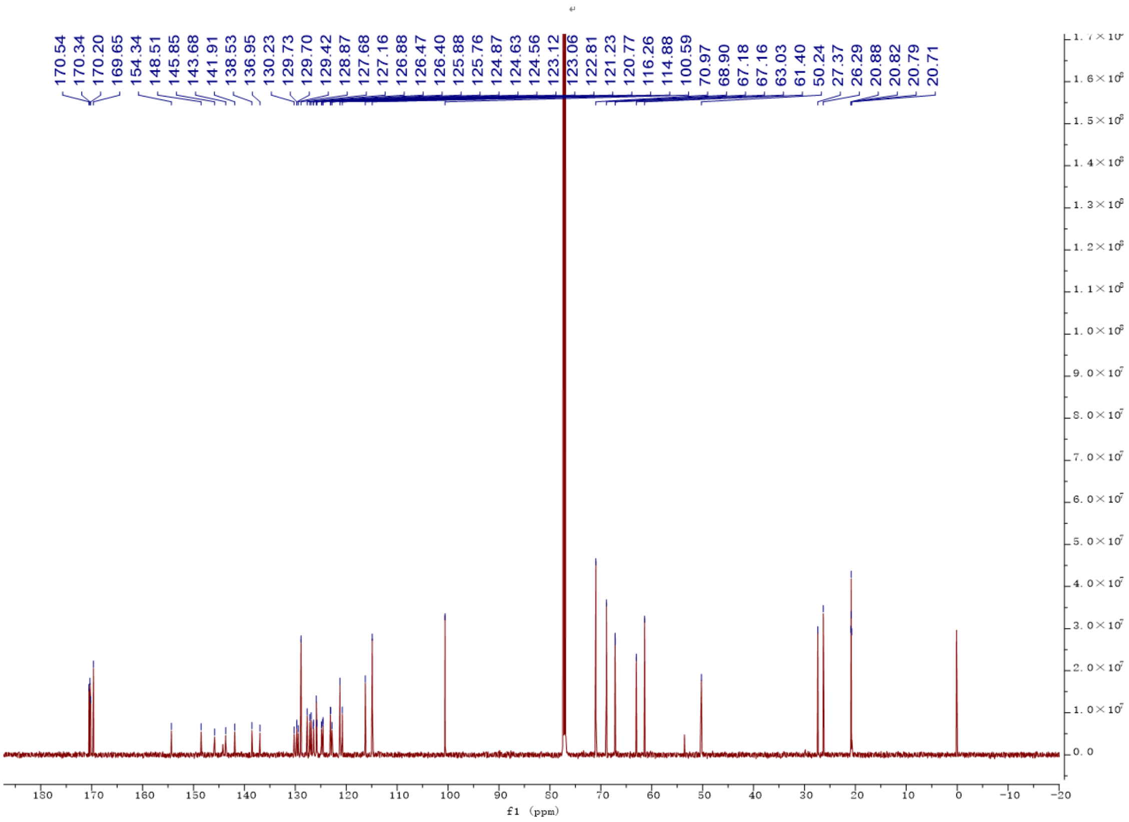

^{13}C NMR of **G13.**

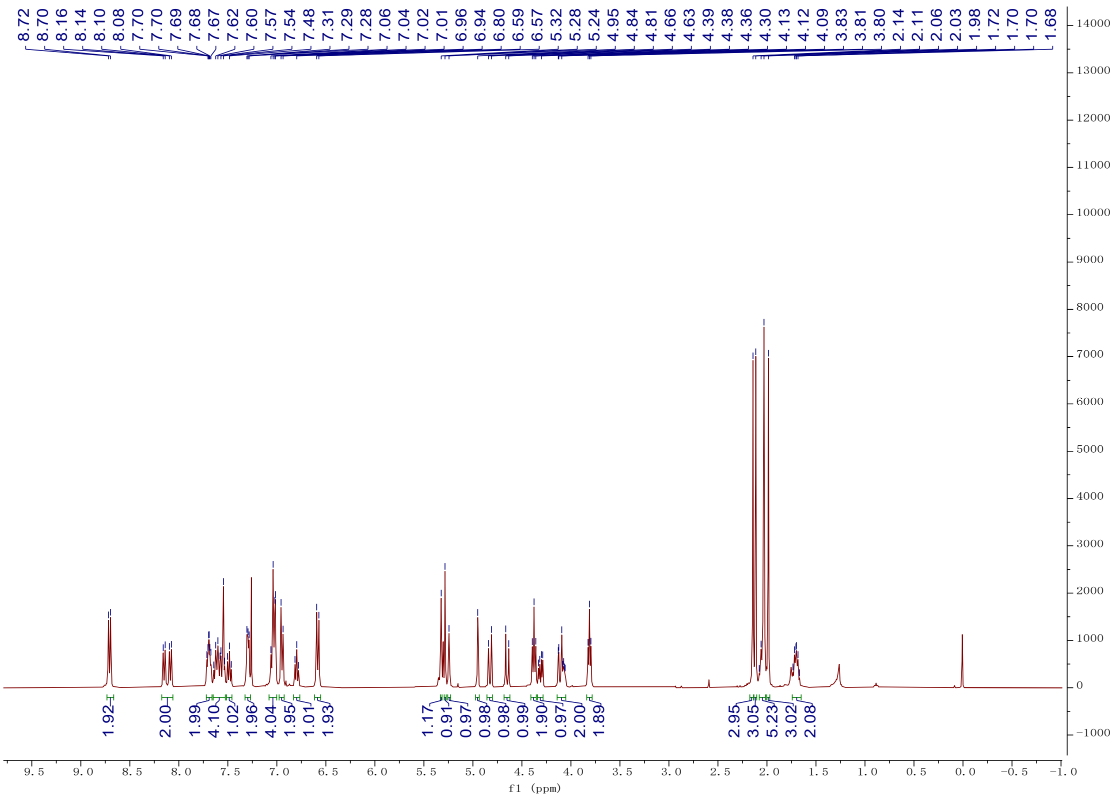

^{1}H NMR of **G14.**

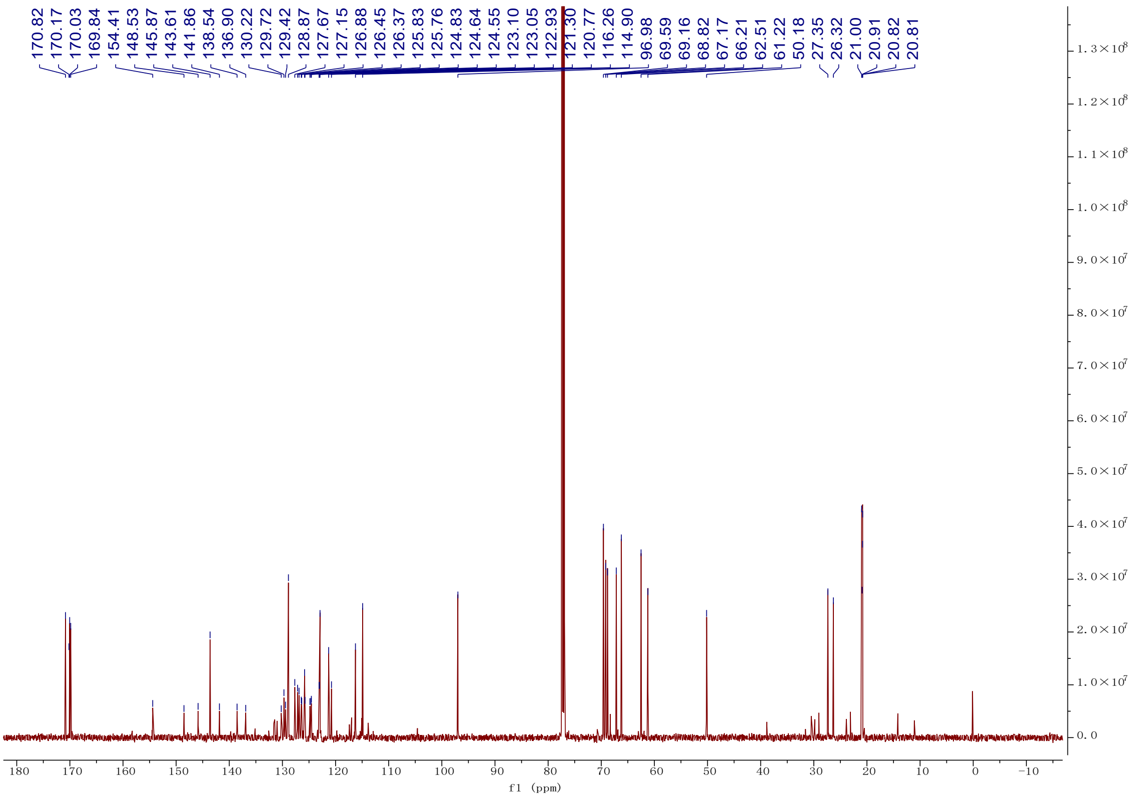

^{13}C NMR of **G14.**

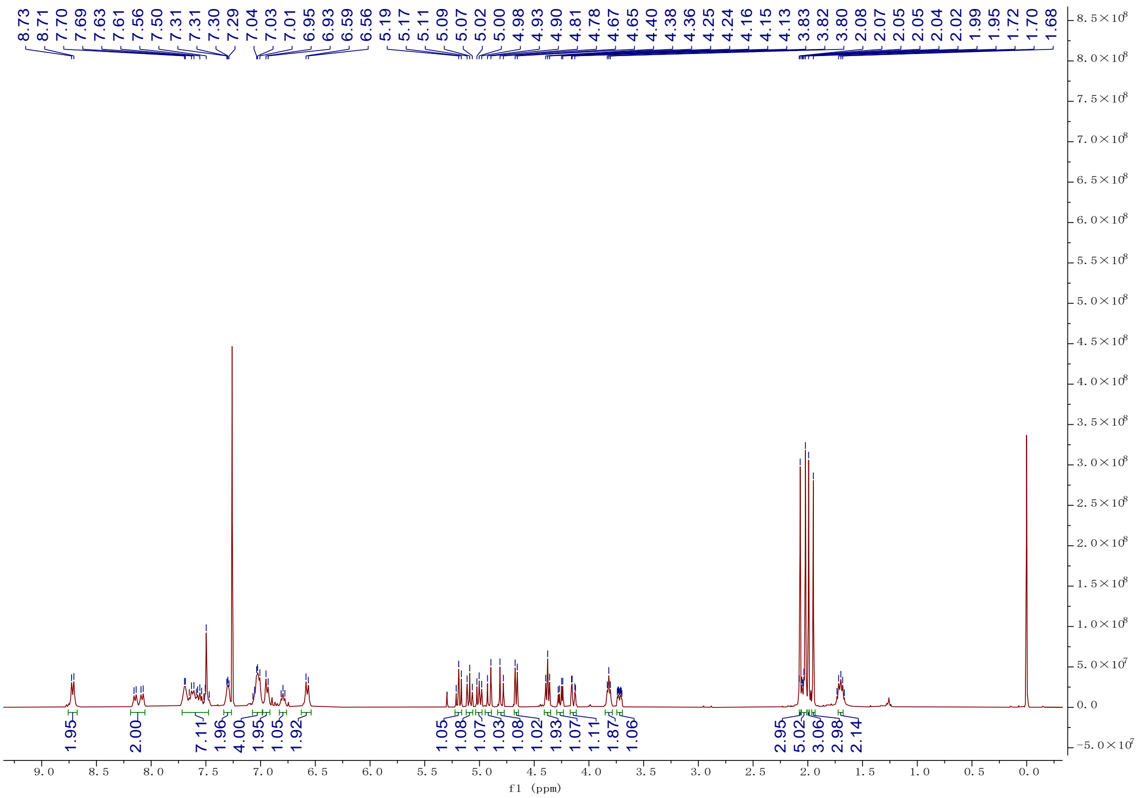

^{1}H NMR of **G15.**

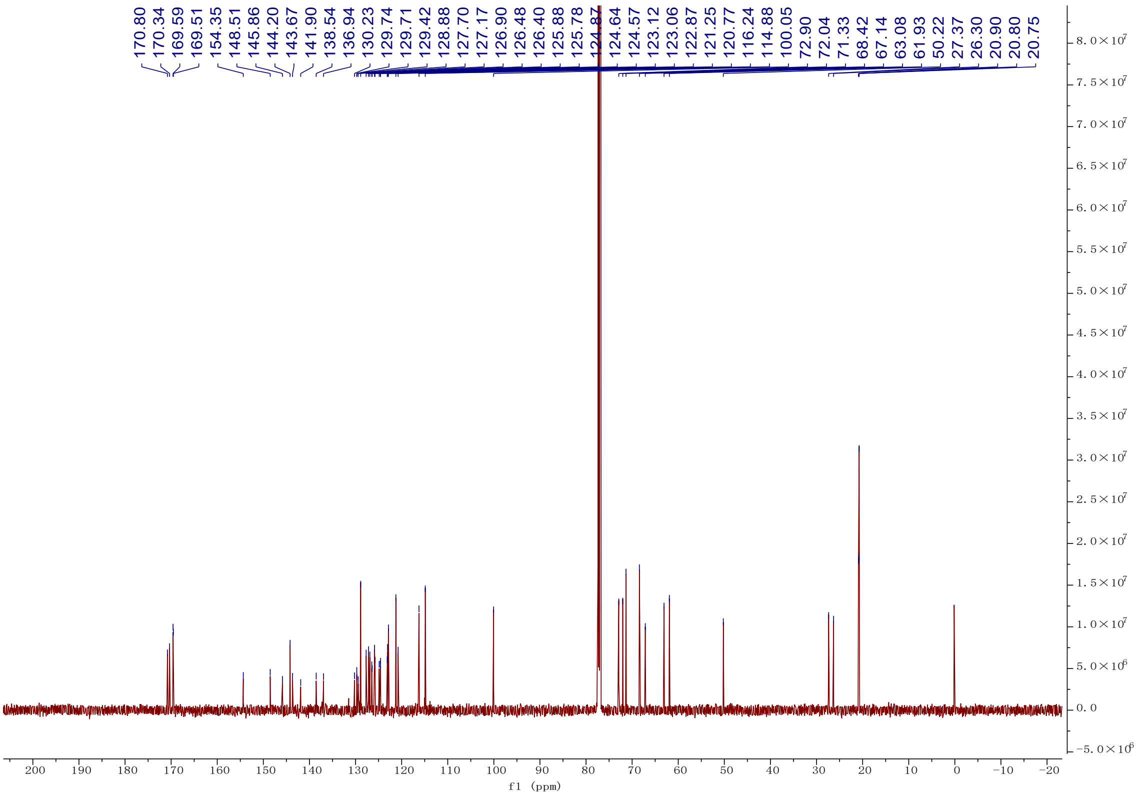

^{13}C NMR of **G15.**

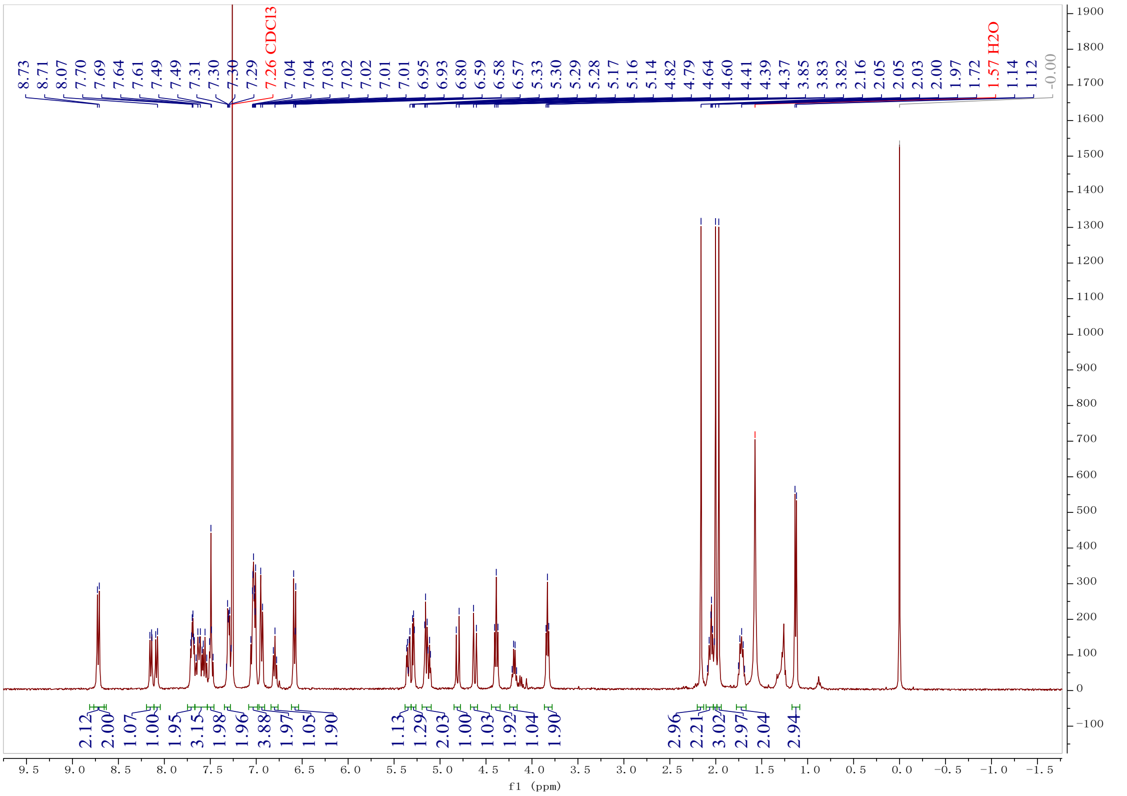

^{1}H NMR of **G16.**

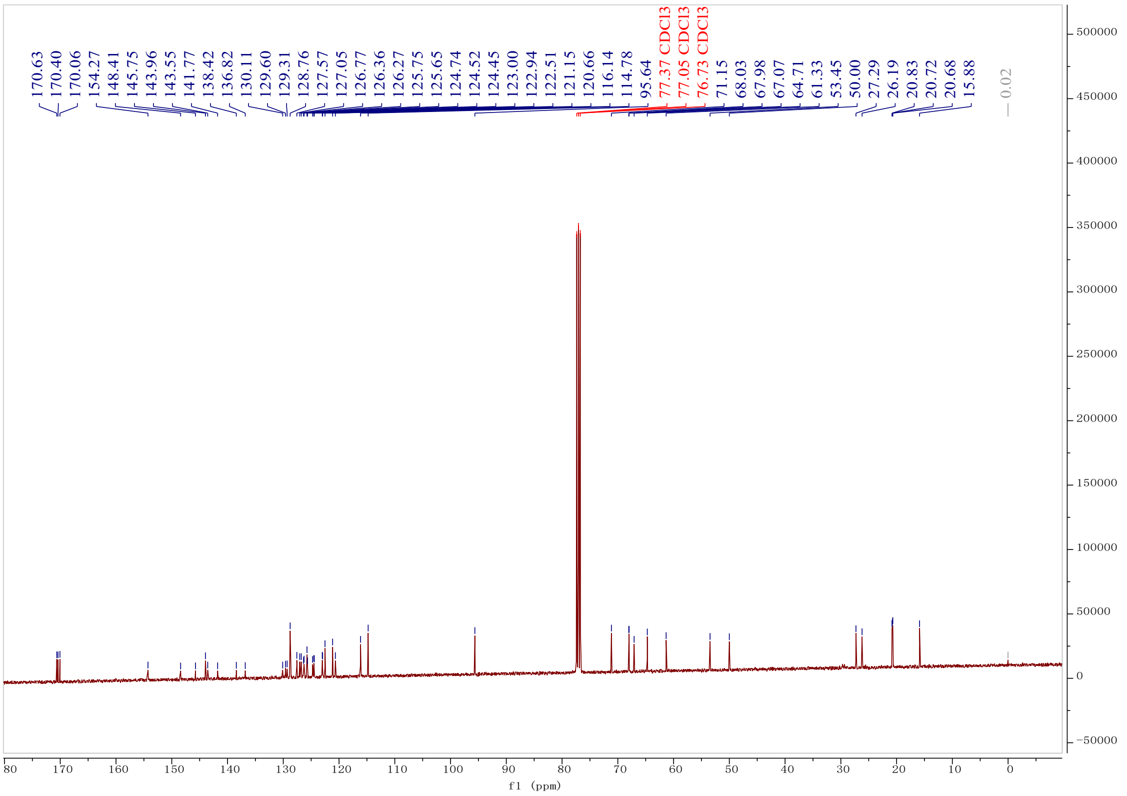

^{13}C NMR of **G16.**

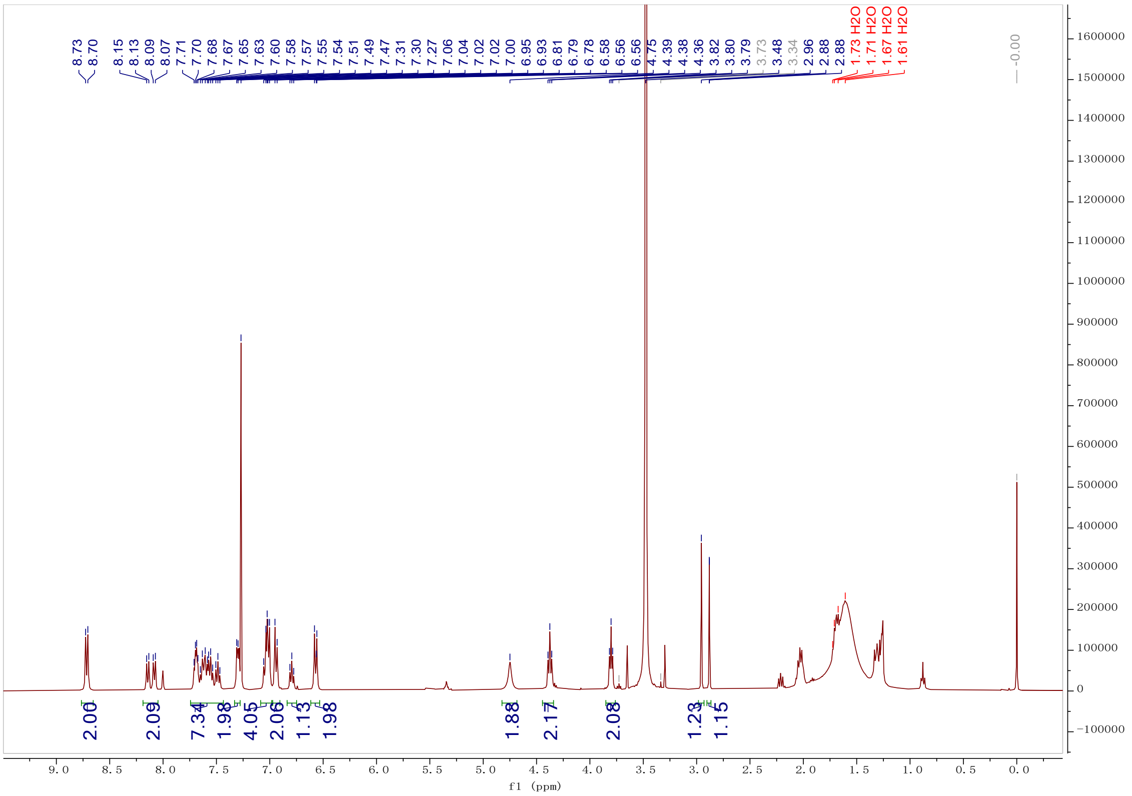

^{1}H NMR of **DPAC-OH**

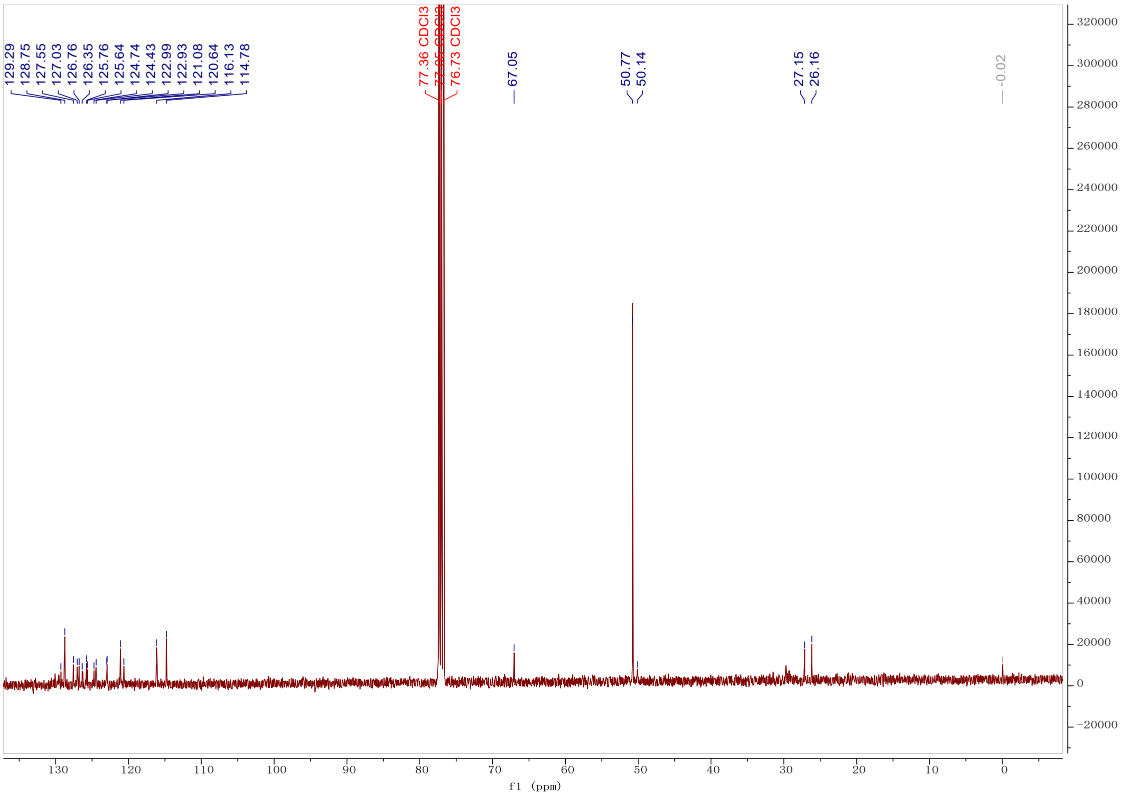

^{13}C NMR of **DPAC-OH**

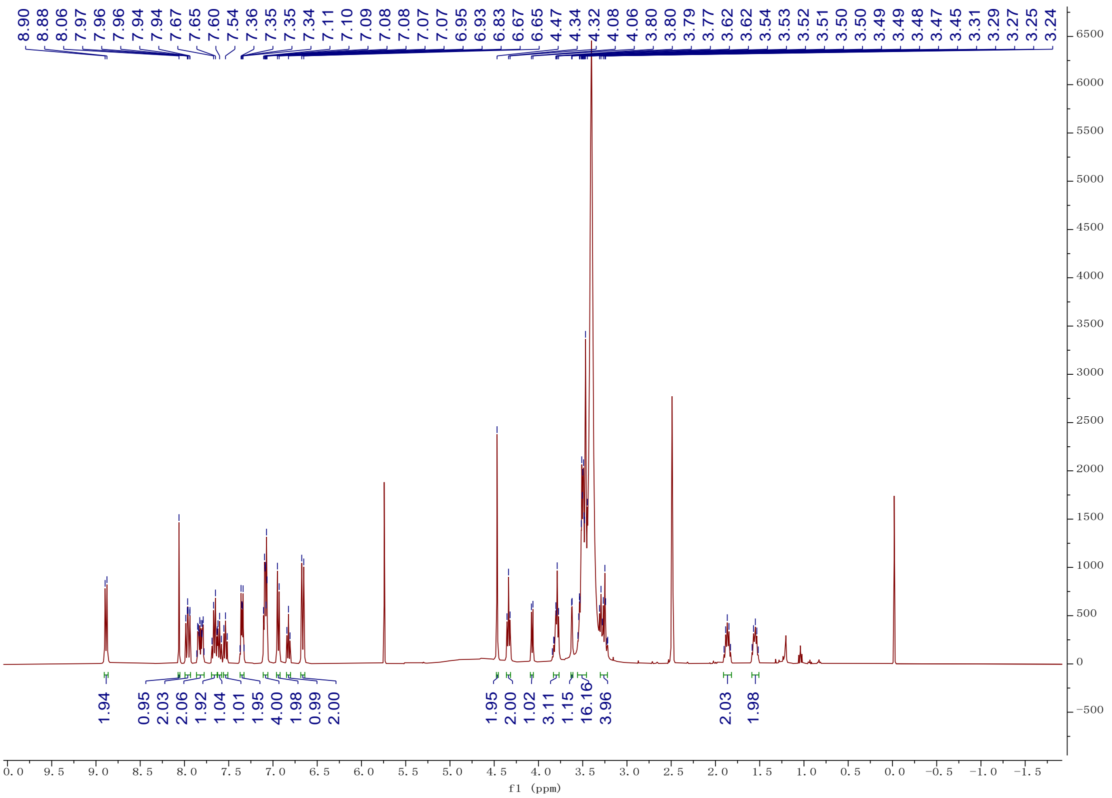

^{1}H NMR of **DPAC-PEG$_3$-Gal.**

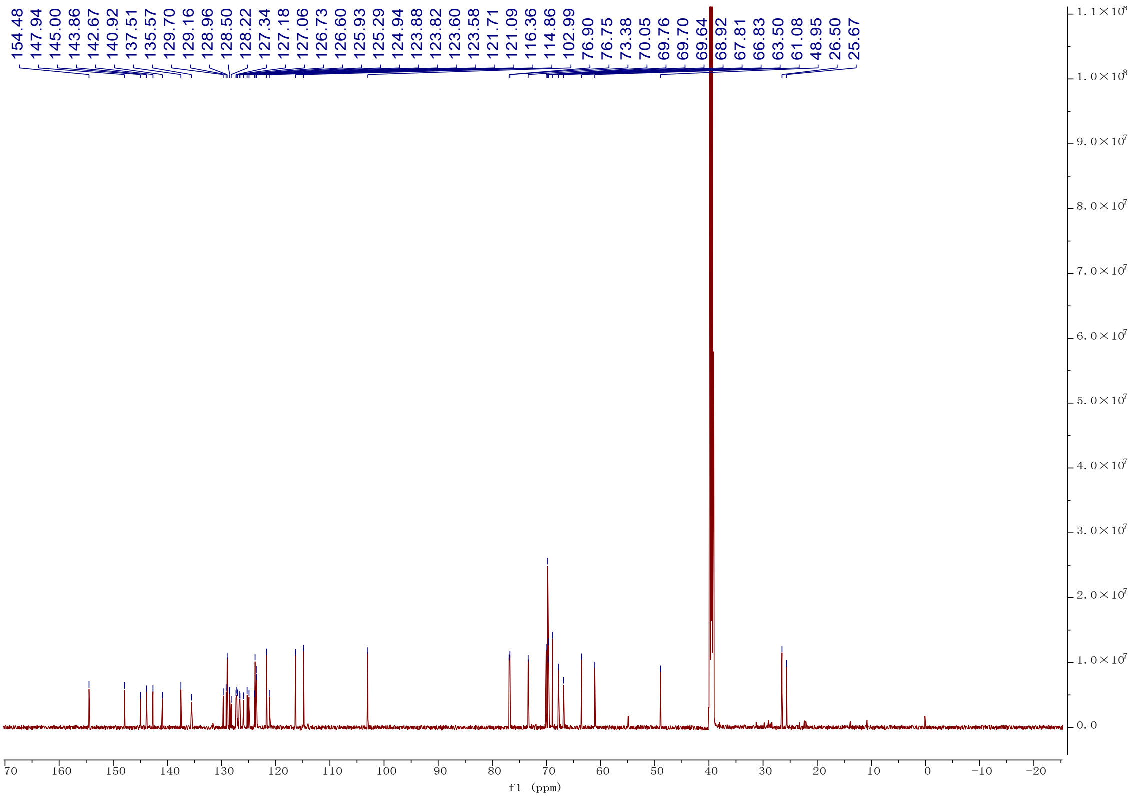

^{13}C NMR of **DPAC-PEG$_3$-Gal.**

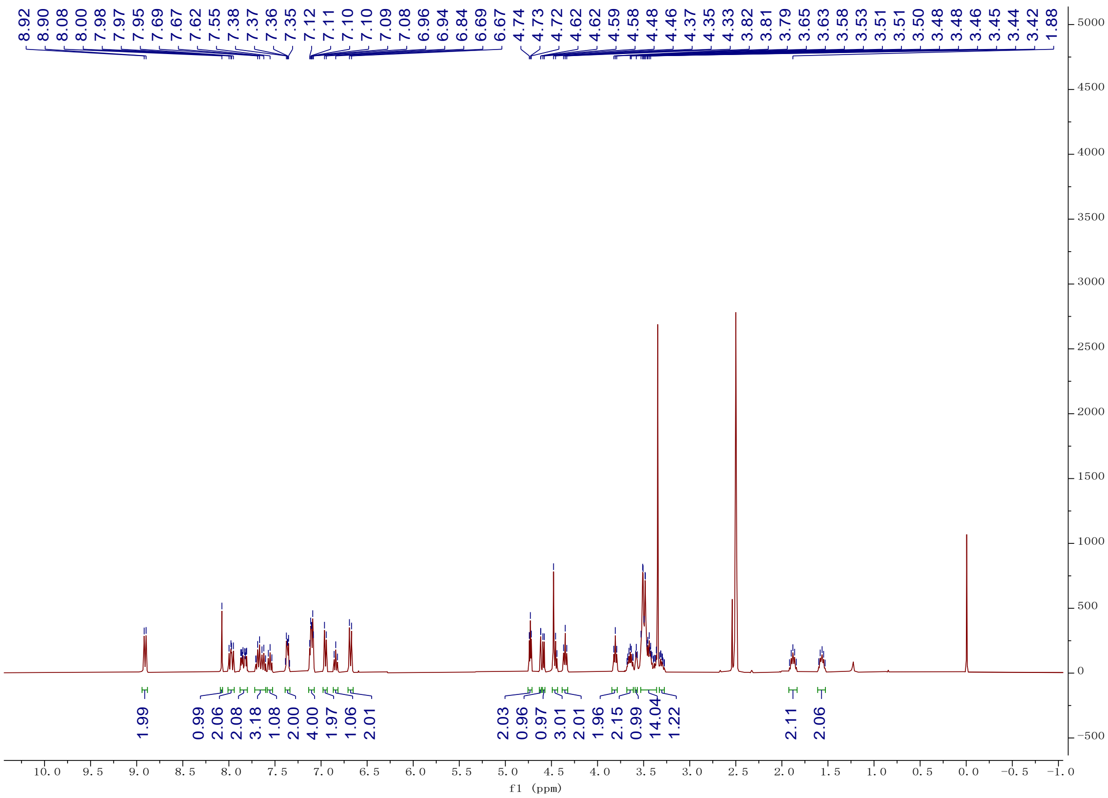

^{1}H NMR of **DPAC-PEG$_3$-Man.**

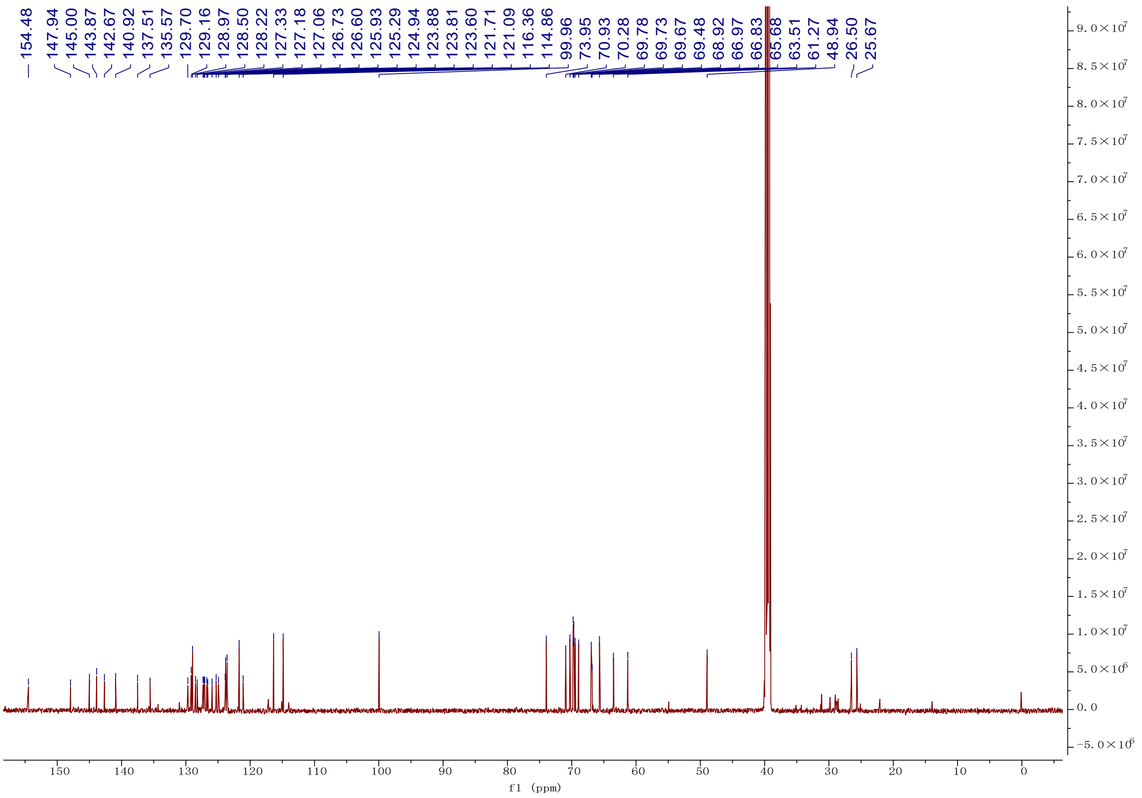

^{13}C NMR of **DPAC-PEG$_3$-Man.**

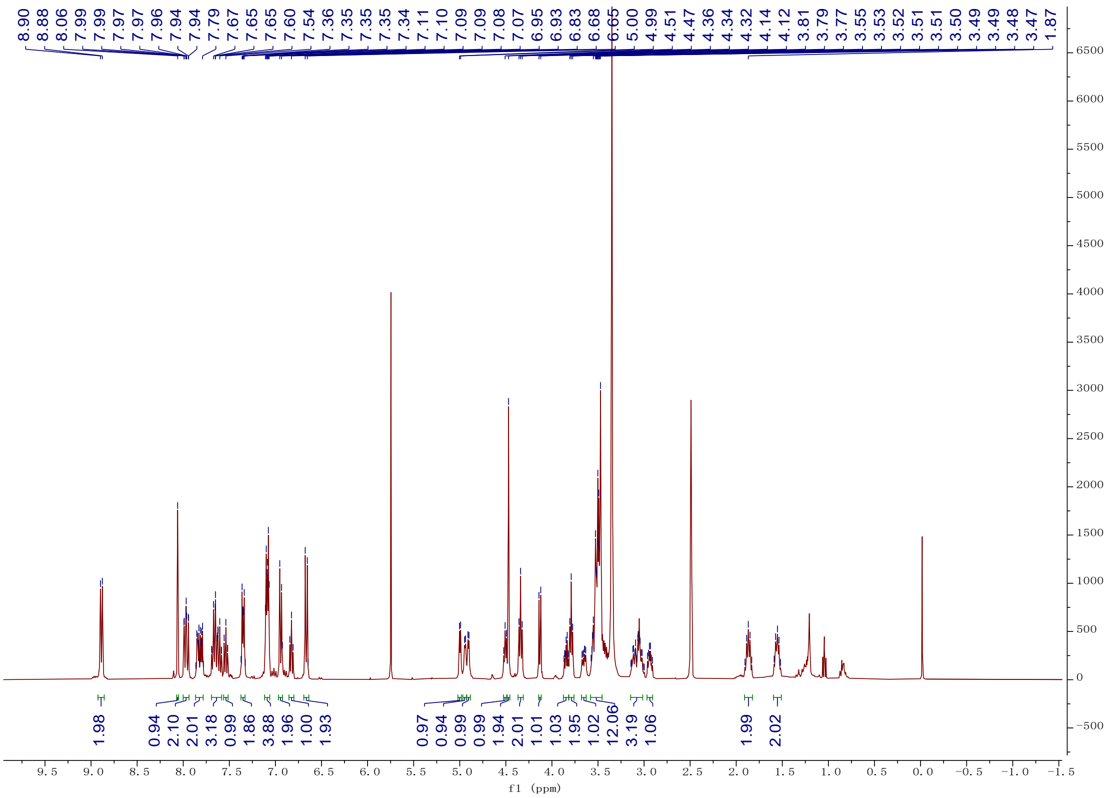

^1H NMR of **DPAC-PEG$_3$-Glc.**

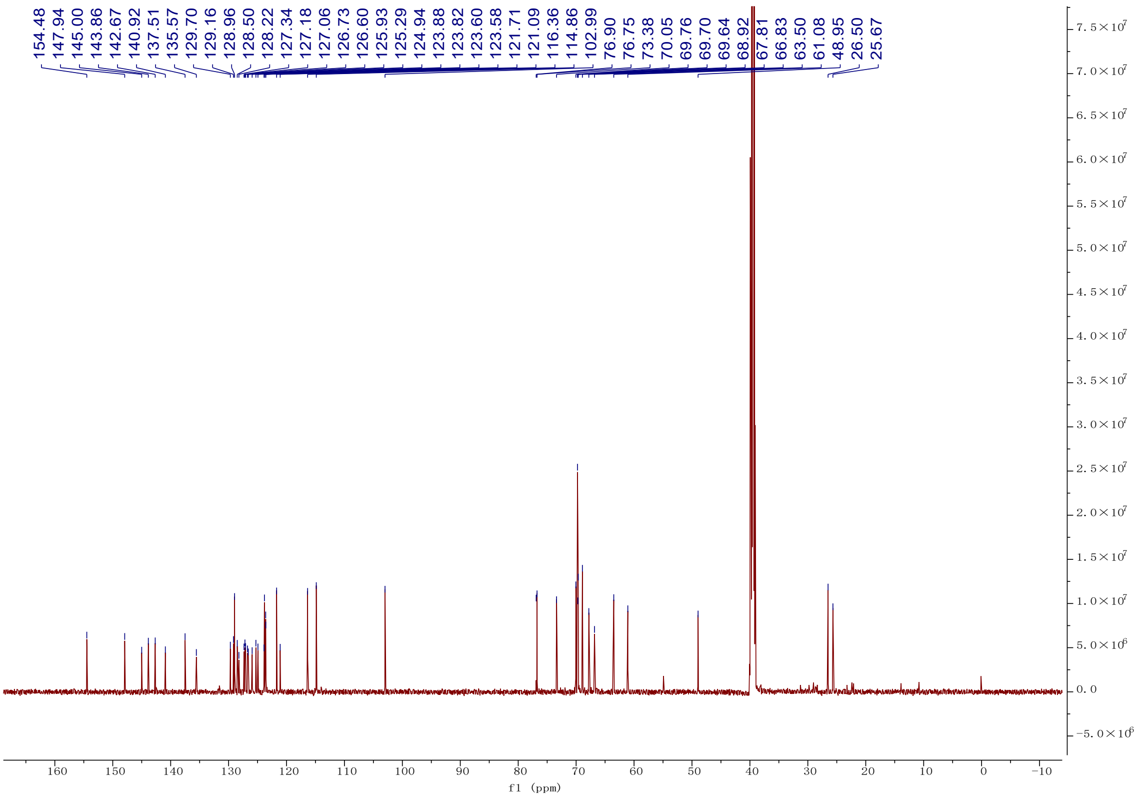

^{13}C NMR of **DPAC-PEG$_3$-Glc.**

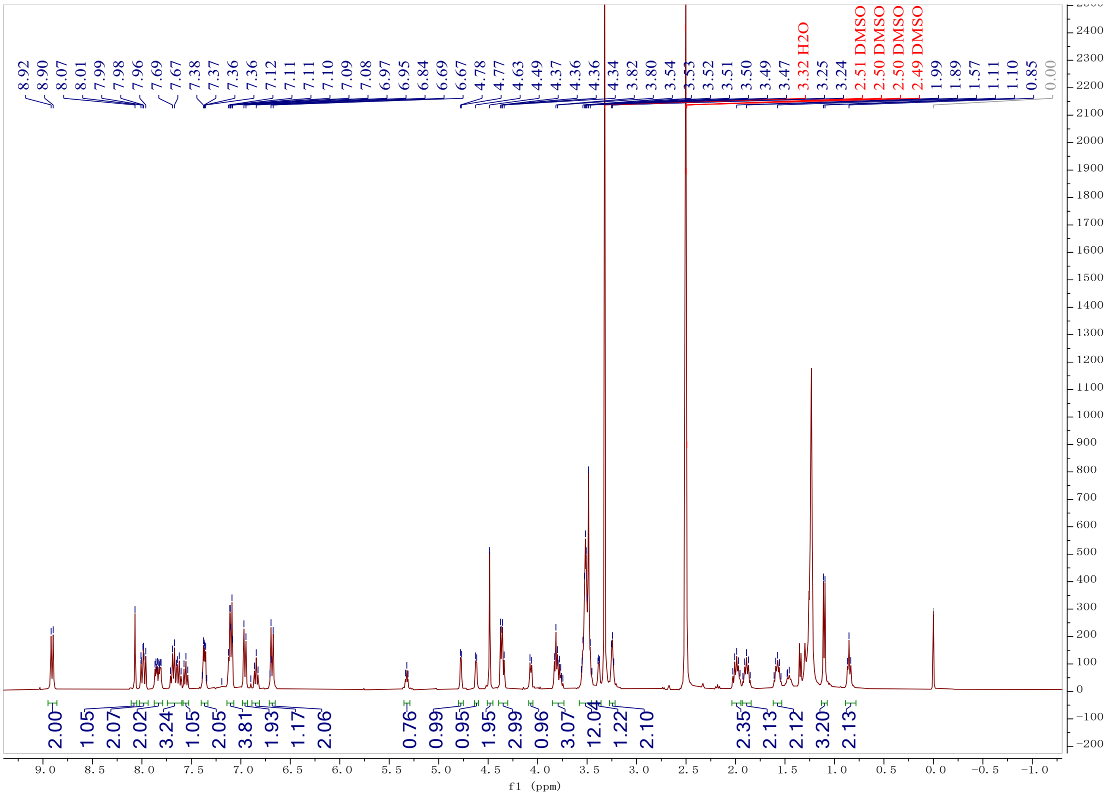

^{1}H NMR of **DPAC-PEG$_3$-Fuc.**

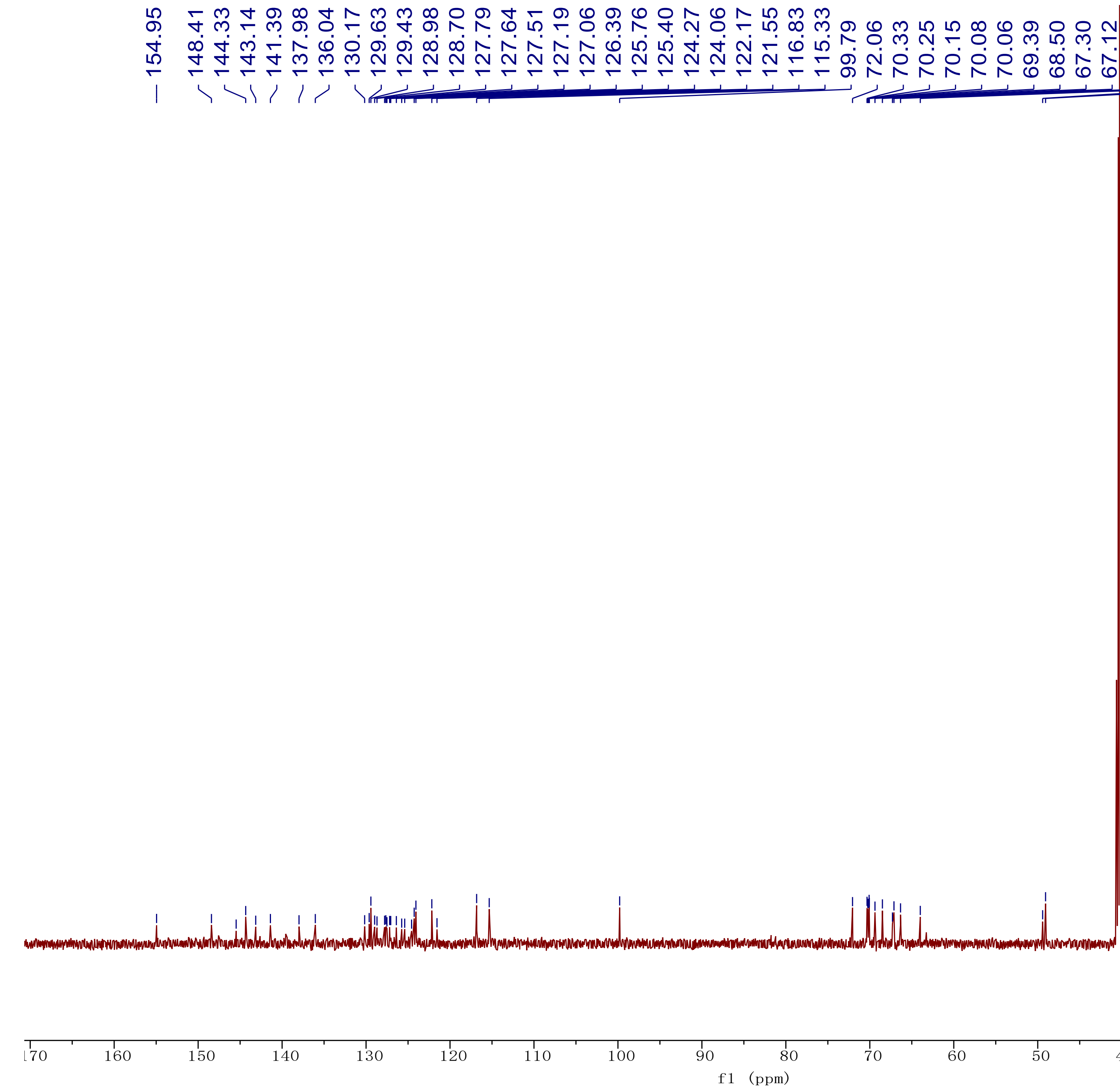

^{13}C NMR of **DPAC-PEG$_3$-Fuc.**

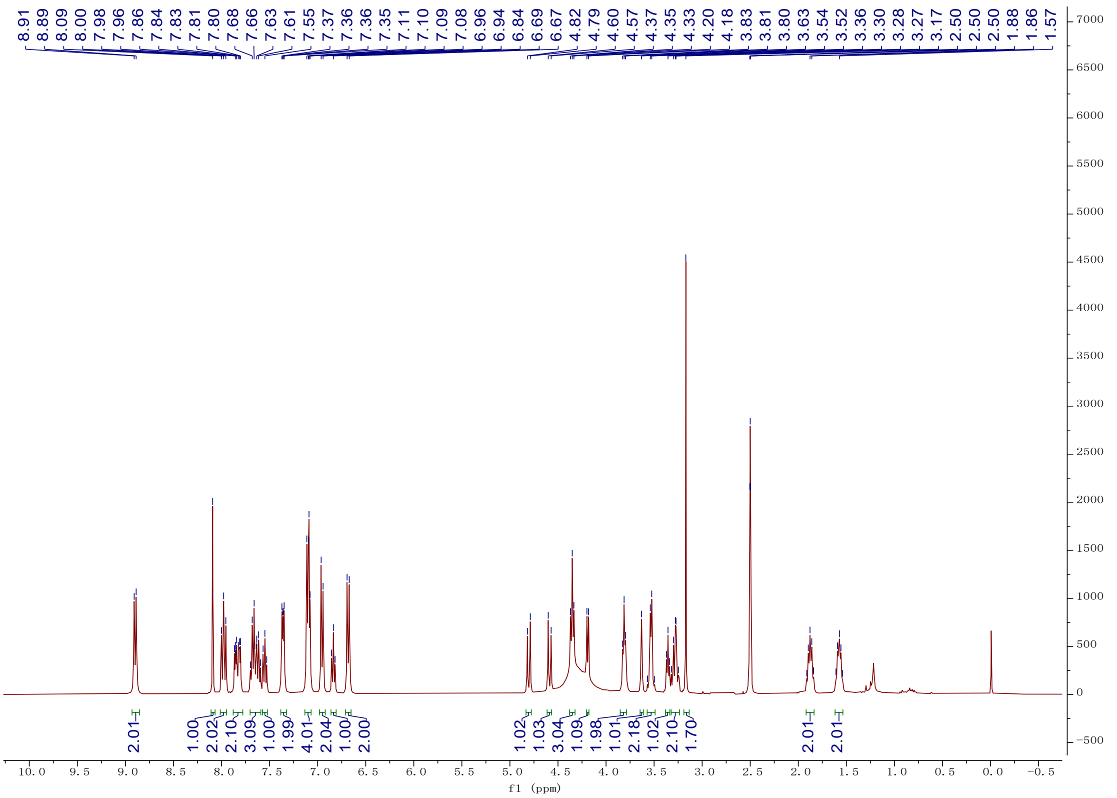

^{1}H NMR of **DPAC-Gal.**

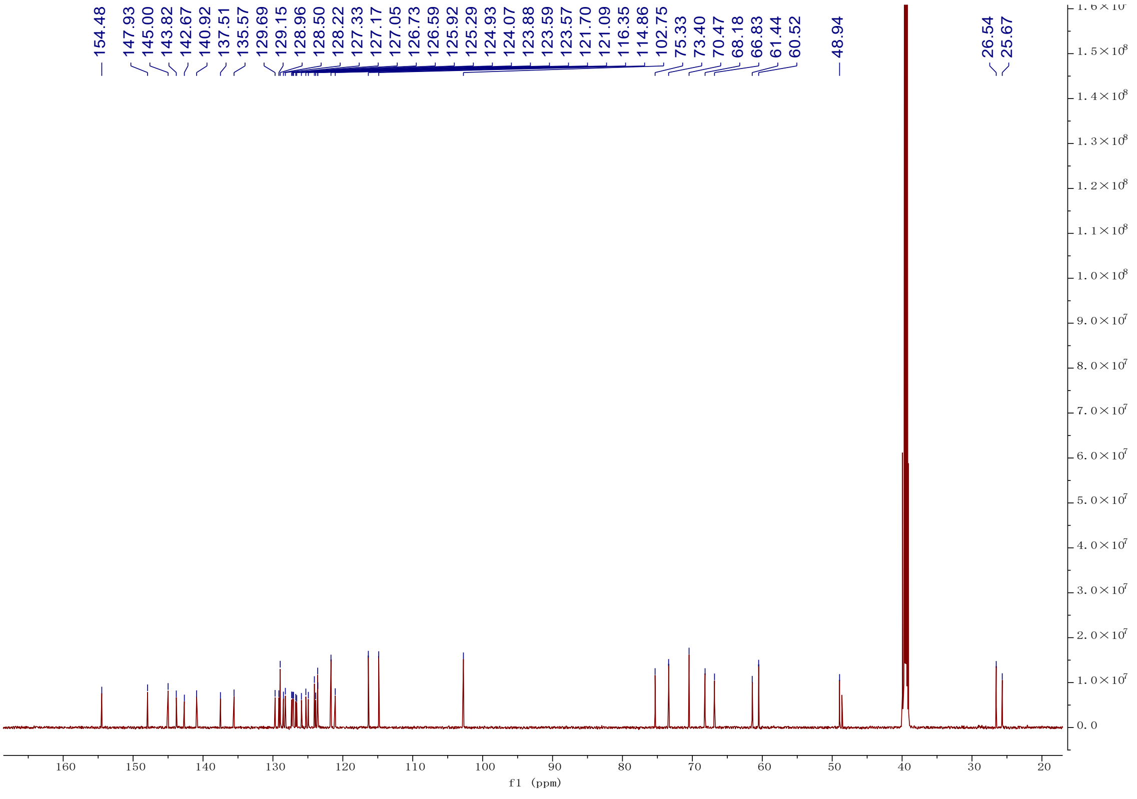

^{13}C NMR of **DPAC-Gal**

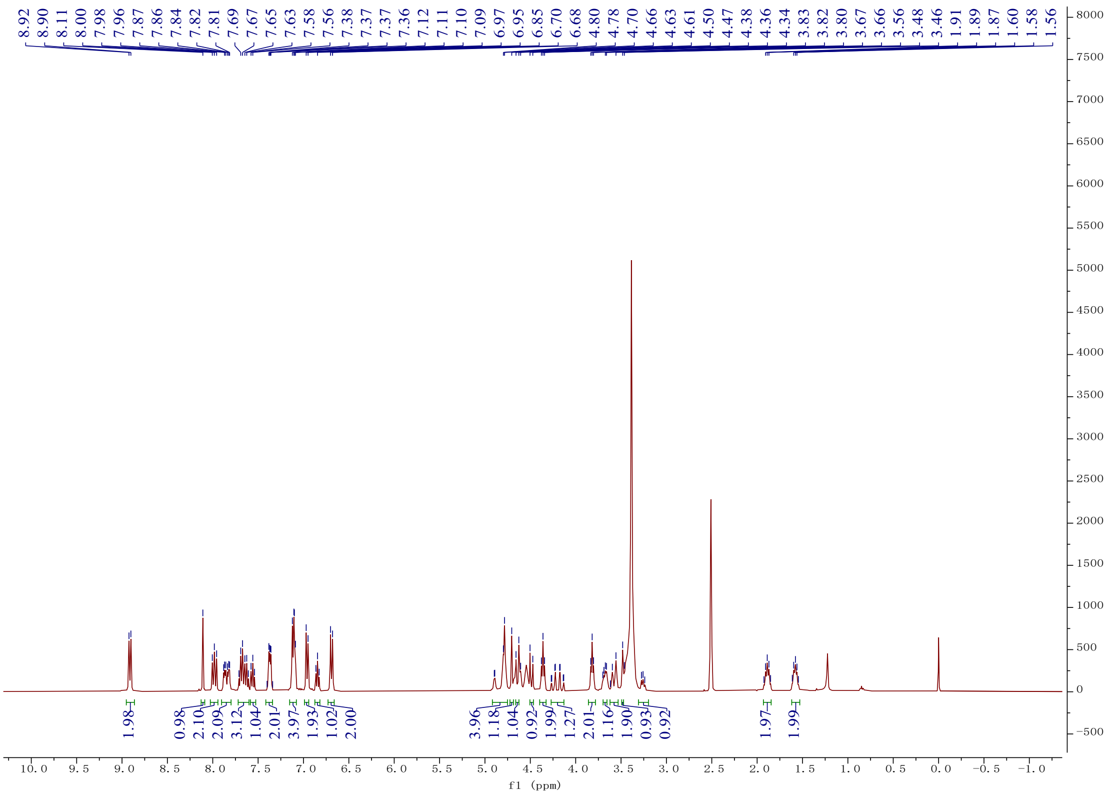

^{1}H NMR of **DPAC-Man.**

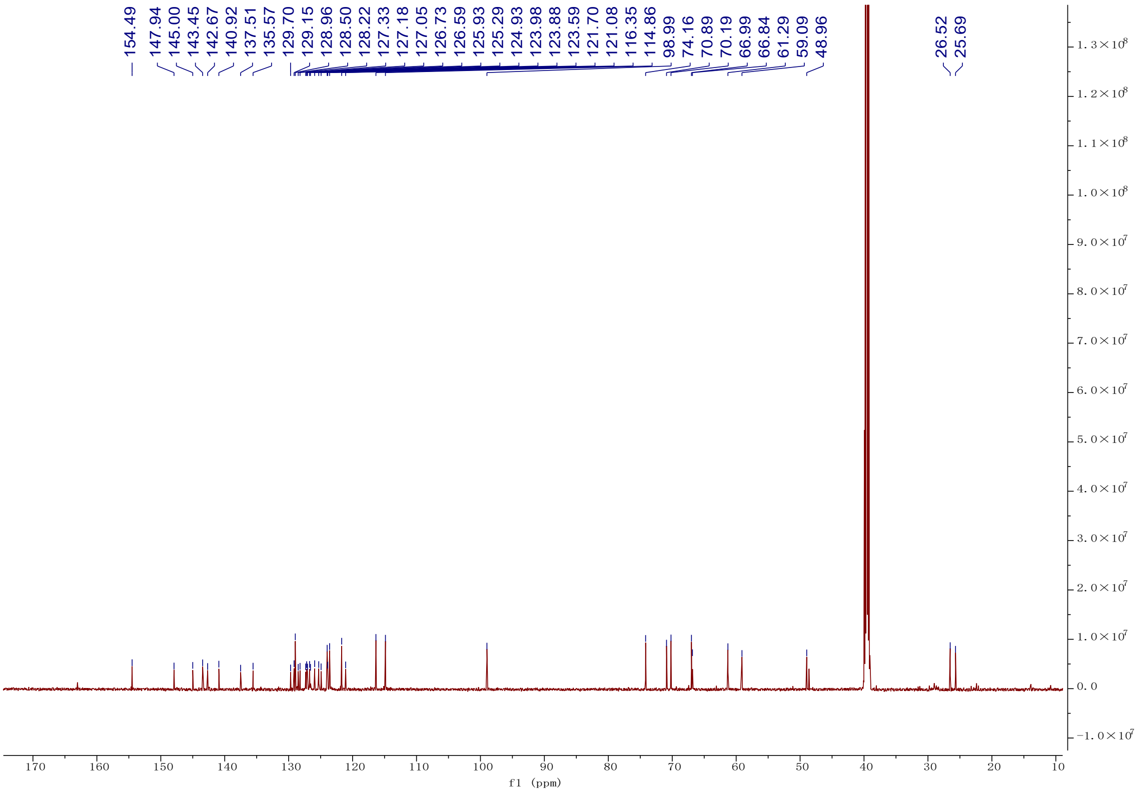

^{13}C NMR of **DPAC-Man.**

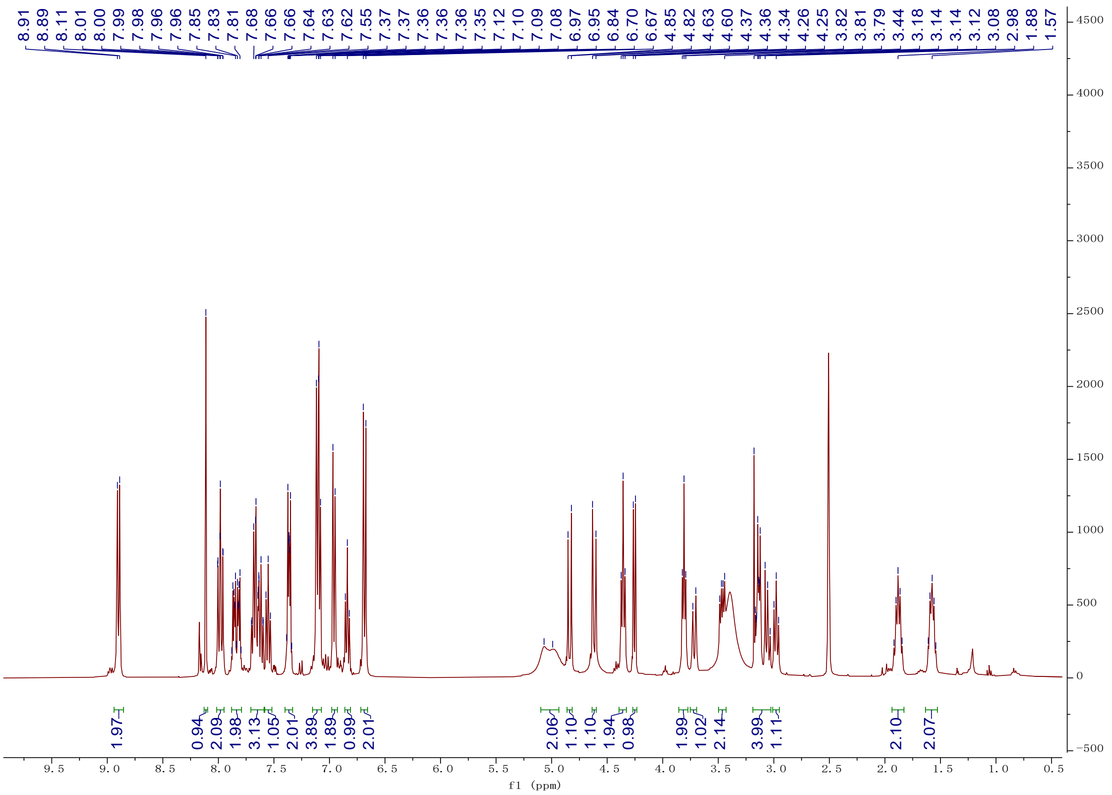

^{1}H NMR of **DPAC-Glc.**

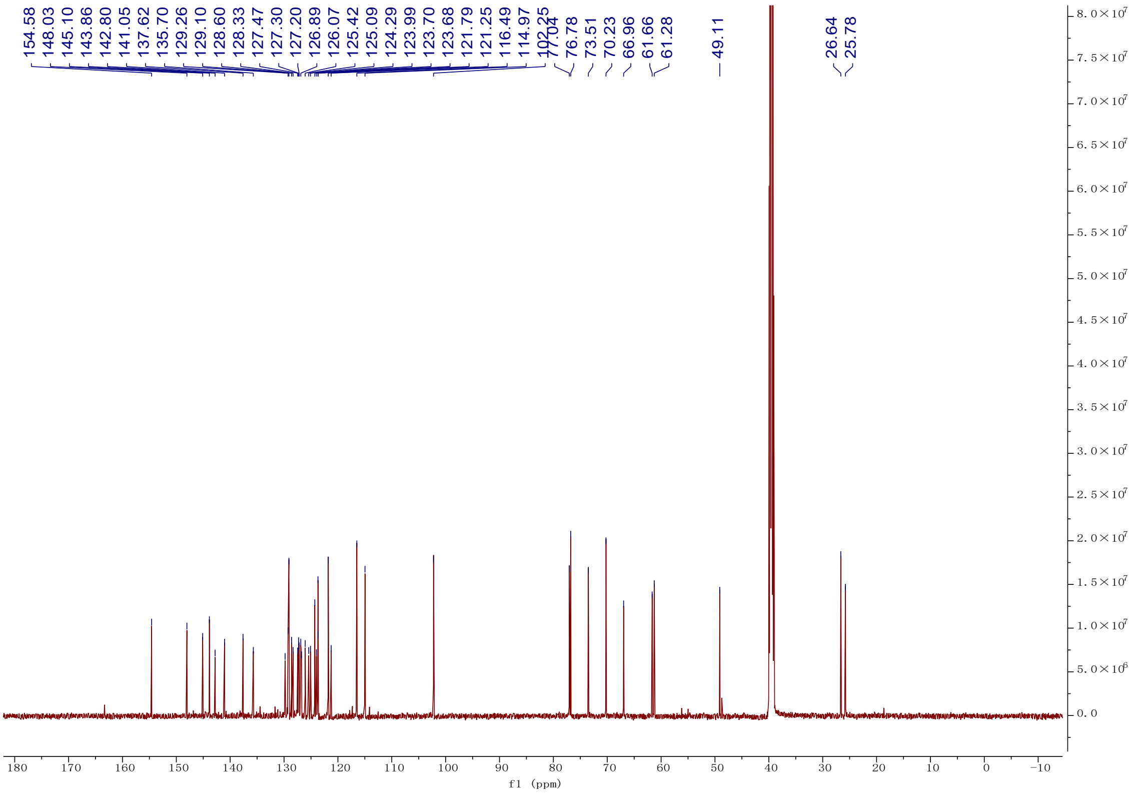

^{13}C NMR of **DPAC-Glc.**

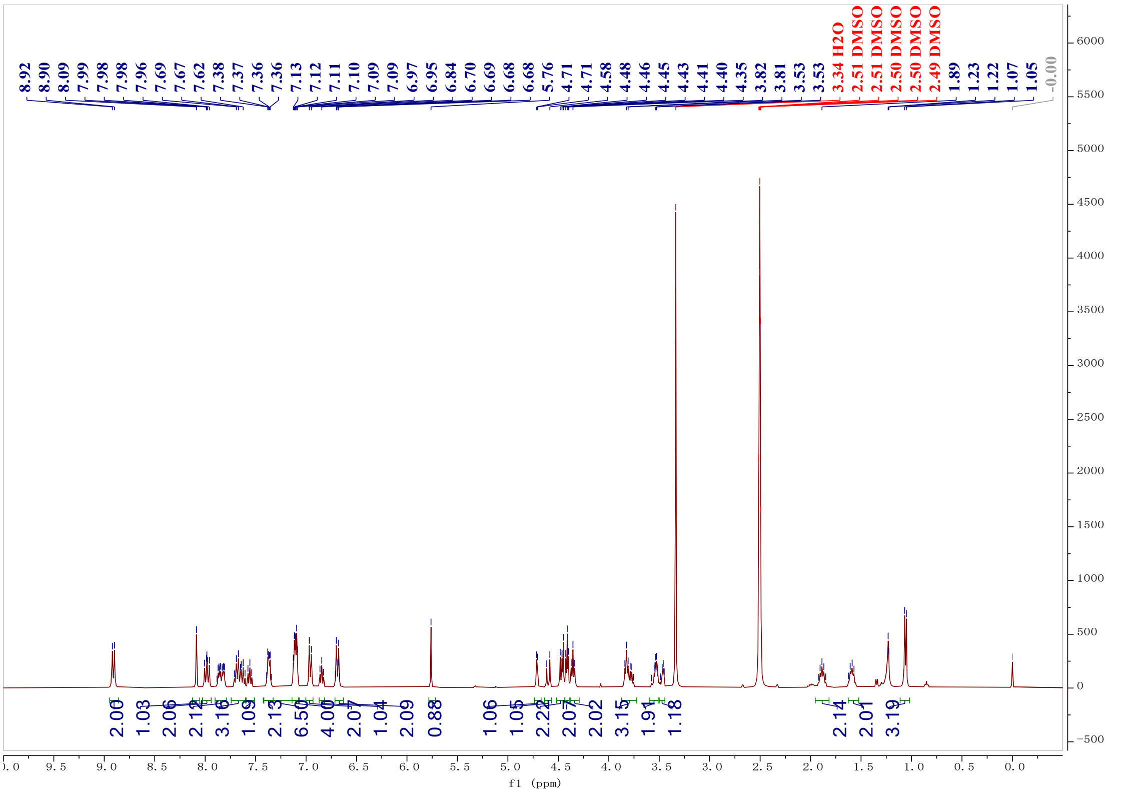

^{1}H NMR of **DPAC-Fuc**

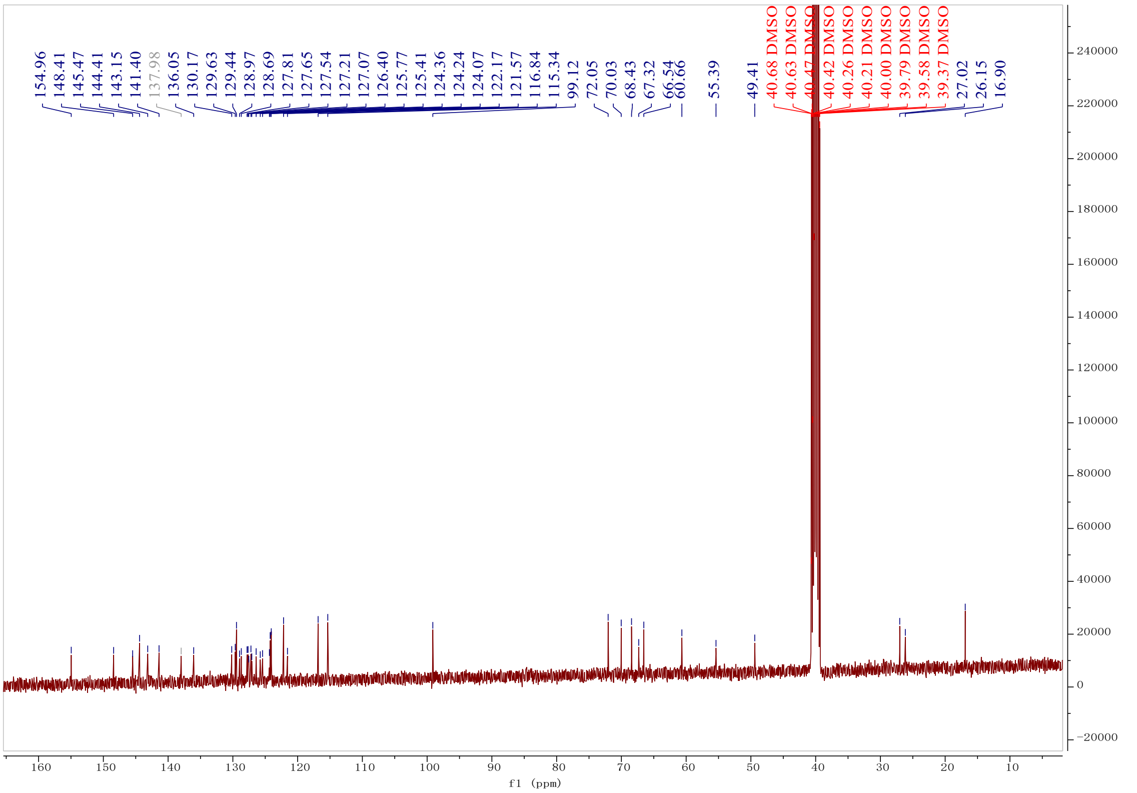

^{13}C NMR of **DPAC-Fuc.**

S4. Additional references

1. Y. X. Ding, Q. Wu, Y. Guo, M. Li, P. F. Li, Y. Ma and W. C. Liu, Effects of in vitro-induced drug resistance on the virulence of *Streptococcus*, *Vet. Med. Sci.*, 2021, **7**, 935-943.

2. J. E. Pollard, J. Snarr, V. Chaudhary, J. D. Jennings, H. Shaw, B. Christiansen, J. Wright, W. Jia, R. E. Bishop and P. B. Savage, In vitro evaluation of the potential for resistance development to ceragenin CSA-13, *J. Antimicrob. Chemother.*, 2012, **67**, 2665-2672.
